# Transcriptomes of a xylose-utilizing industrial flocculating *Saccharomyces cerevisiae* strain cultured in media containing different sugar sources

**DOI:** 10.1186/s13568-016-0223-y

**Published:** 2016-08-02

**Authors:** Wei-Yi Zeng, Yue-Qin Tang, Min Gou, Zi-Yuan Xia, Kenji Kida

**Affiliations:** College of Architecture and Environment, Sichuan University, No. 24, South Section 1, First Ring Road, Chengdu, 610065 Sichuan China

**Keywords:** Transcriptome, *Saccharomyces cerevisiae*, Xylose fermentation, Bioethanol, Glucose and xylose cofermentation

## Abstract

Lignocellulosic hydrolysates used for bioethanol production contain a mixture of sugars, with xylose being the second most abundant after glucose. Since xylose is not a natural substrate for *Saccharomyces cerevisiae*, recombinant *S. cerevisiae* strongly prefers glucose over xylose, and the fermentation rate and ethanol yield with xylose are both lower than those with glucose. To determine the molecular basis for glucose and xylose fermentation, we used microarrays to investigate the transcriptional difference of a xylose-utilizing industrial strain cultured in both single sugar media and a mixed sugar medium of glucose and xylose. The transcriptomes were nearly identical between glucose metabolizing cells in the glucose alone medium and those in the glucose fermentation phase in the mixed-sugar medium. Whereas the transcriptomes highly differed between the xylose metabolizing cells in the xylose alone medium and those in the xylose fermentation phase in the mixed sugar medium, and the differences mainly involved sulfur metabolism. When the transcriptional profiles were compared between glucose fermentation state and xylose fermentation state, we found the expression patterns of hexose transporters and glucose signaling pathway differed in response to different sugar sources, and the expression levels of the genes involved in gluconeogenesis, the glyoxylate and tricarboxylic acid cycles and respiration increased with xylose, indicating that the xylose-metabolizing cells had high requirements for maintenance energy and lacked the carbon catabolite repression capability. The effect of carbon catabolite repression by glucose lasted after glucose depletion for specific genes to different extents.

**Electronic supplementary material:**

The online version of this article (doi:10.1186/s13568-016-0223-y) contains supplementary material, which is available to authorized users.

## Introduction

Lignocellulosic biomass has been recognized as a sustainable source for fuel ethanol production without affecting the food and feed markets. It is of economic interest to convert all lignocellulosic sugar fractions, predominantly glucose and xylose, into ethanol at sufficiently high rates and yields. However, *Saccharomyces cerevisiae,* which is widely used in bioethanol plants due to its high fermentation efficiency and process robustness, cannot ferment xylose (Batt et al. 1986).

In the past two decades, fermentation of xylose to ethanol has been achieved in *S. cerevisiae* by genetic engineering. Through expression of the heterogeneous xylose metabolic pathway—either xylose reductase-xylitol dehydrogenase (XR-XDH) or xylose isomerase (XI)—*S. cerevisiae* can convert xylose to xylulose, which can then be natively catabolized (Matsushika et al. 2009a). The xylose-utilizing capacity of the recombinant strains can be further optimized by enhancing the downstream metabolic pathway rationally or through evolutionary engineering (Peng et al. 2012). However, recombinant strains strongly prefer glucose over xylose, and therefore the co-consumption remains a challenge. What’s more, the specific ethanol productivity from xylose was an order of magnitude lower than that from glucose, despite tremendous efforts, and the ethanol yield from xylose was lower than that from glucose as well (Matsushika et al. 2009b).

To reveal the major reasons for the suboptimal fermentation of xylose by recombinant *S. cerevisiae* strains, the difference in transcriptional response between xylose and glucose fermentation has been examined in the past decade. It has been recognized that *S. cerevisiae* does not sense xylose as a fermentable carbon source (Jin et al. 2004). Early transcriptional analysis on xylose was conducted during aerobic growth, and revealed that xylose was neither recognized as a fermentable carbon source nor as a respirative carbon source (Salusjärvi et al. 2006). Using transcriptome and proteome, the difference in carbon source signaling and catabolite repression was studied in the aerobic batch fermentation of either glucose or xylose, and it has been suggested that cells metabolizing xylose were neither in a completely repressed nor in a derepressed state (Salusjärvi et al. 2008). The transcriptional difference in *S. cerevisiae* growing anaerobically in either glucose or xylose was subsequently analyzed (Matsushika et al. 2014; Runquist et al. 2009), indicating xylose was recognized as a non-fermentable carbon source and induced the expression of stress-responsive genes. More recently, the specific regulatory response of *S. cerevisiae* to xylose was quantified at a range of cultivation times in anaerobic glucose-xylose mixed medium (Alff-Tuomala et al. 2016), and xylose was observed to delay the glucose-dependent repression of specific genes in the mixed culture.

To the best of our knowledge, the transcription and regulatory responses induced by xylose have been analyzed either in single sugar or mixed sugar cultures. As the transcriptional profile is dependent on different host strain background (Feng and Zhao 2013a), a systematic transcriptional analysis of a particular *S. cerevisiae* strain under different fermentation conditions, including in media with a single sugar and mixed sugars, would be useful to identify the genetic factors responsible for the discrepancy in sugar source utilization efficiency. Industrial *S. cerevisiae* strains generally have a superior ethanol production efficiency and inhibitor tolerance compared to laboratory strains. In this study, we therefore carried out batch fermentations of KF7M-16, an XR- and XDH-expressing industrial flocculating strain, in both single sugar medium (either glucose or xylose) and mixed sugar medium (including the glucose fermentation phase and the xylose fermentation phase), and analyzed the global transcriptomes based on microarrays to identify the molecular response to different fermentation states. It has been reported that cellular growth rate has great influence on transcriptional regulation (Regenberg et al. 2006), we therefore adopt high-density inoculations to attenuate the possible impact of different growth rates on transcriptome analysis. This is the first study investigating transcriptional difference of an industrial *S. cerevisiae* strain in both single sugar media and mixed sugar medium.

## Materials and methods

### Strains and batch fermentation conditions

The recombinant xylose-utilizing *S. cerevisiae* used in this study was KF7M-16 derived from KF-7 (Kida et al. 1992). Two integrative plasmids were transformed into KF-7: pIUX1X2XK (contains *XYL1,XYL2* from *Scheffersomyces stipitis* and *XKS1* from *S. cerevisiae*) and pIWBGL1 (contains *BGL1* from *Aspergillus aculeatus*) (Li et al. 2015).

The single and mixed sugar media used in the batch fermentation were as follows: 6 % YPD (20 g/L peptone, 10 g/L yeast extract, and 60 g/L glucose), 4 % YPX (20 g/L peptone, 10 g/L yeast extract, and 40 g/L xylose), and 10 % YPDX (20 g/L peptone, 10 g/L yeast extract, 60 g/L glucose, and 40 g/L xylose).

Batch fermentations were conducted in 300-ml cotton-plugged shake-flasks containing 100 ml medium, at a stirring speed of 200 rpm controlled by a HS-6DN magnetic stirrer (As One, Japan). The temperature was maintained at 35 °C in a thermostatic water bath.

Working cultures of yeasts were obtained following an activation period at 30 °C for 24 h on a 2 % YPD plate (20 g/L peptone, 10 g/L yeast extract, 20 g/L glucose with 2 % agar) and then pre-cultivating the yeast cells at 30 °C for 16 h in 5 % YPD (20 g/L peptone, 10 g/L yeast extract, and 50 g/L glucose). The harvested cells were inoculated into batch fermentation at 5 g-dry cell weight/L (g-DCW/L).

For the pre-experiment, the culture was sampled every 2 h in the fast fermenting stage to monitor cell growth and metabolites concentration. The experiments for microarray analysis were conducted in biological duplicate, and samples were collected at the time points indicated in the results section.

### RNA extraction

After cell collection, the total RNA was extracted using the Takara Yeast RNAiso Kit according to the manufacturer’s protocol. RNA quality and concentration were measured by agarose gel electrophoresis and NanoDrop 2000/2000C (Thermo Scientific, USA).

### Microarray analysis

Microarray analysis was performed using the 7G Affymetrix GeneChip^®^ Yeast Genome 2.0 Array (CapitalBio Tec., Beijing). The isolated total RNA was cleaned up with RNeasy Kit (Qiagen, Germany). 100 ng of total RNA was used for cDNA synthesis and produce biotin-tagged cRNA with GeneChip IVT Labeling kit (Affymetrix). 15 µg fragmented cRNA, together with contol oligo B2 and eukaryotic hybridization controls, was hybridized to each GeneChip array at 45 °C for 16 h (Affymetrix GeneChipHybridization Oven 640) according to manufacturer’s instructions. After hybridization, the GeneChip arrays were washed, stained with streptavidin phycoerythrinonan (SAPE) with Affymetrix Fluidics Station 450, and then scanned with the Affymetrix GeneChip Scanner 3000 7G. The data extraction and analysis were carried out using Affymetrix GeneChip Command Console Software. The microarray data can be accessed through GEO accession through GSE80748. To identify altered gene expression, the averages of biological duplicates were compared, and fold changes in gene expression ≥2 were considered significant. The gene annotation information was based on the *Saccharomyces* genome database. The pathway terms were enriched using the KEGG orthology based annotation system (KOBAS).

### Quantitative real-time PCR

The cDNA was reverse-transcribed from total RNA using the Takara PrimeScript™ RT reagent Kit with gDNA Eraser (Perfect Real Time). qPCR analysis was performed using the Takara SYBR^®^ Premix Ex Taq™ II (Tli RNaseH Plus) following manufacture’s manual. *ACT1* was used as the normalization standard. The primers used are listed in Additional file 1: Table S1.

### Analytical methods for substrate and products

Metabolite analysis was conducted as previously described (Tang et al. 2006). Glucose and xylose in the fermentation medium was determined by a HPLC equipped with a fluorescence detector (RF-10A_XL_). Ethanol was measured by GC with a FID detector and 2-propanol was used as the internal standard. Xylitol was assayed by HPLC equipped with an Aminex HPX-87H column (300 × 7.8 mm) (Bio-Rad, USA) and a refractive index detector.

## Results

### Experimental design and batch fermentation

**Fig. 1:**
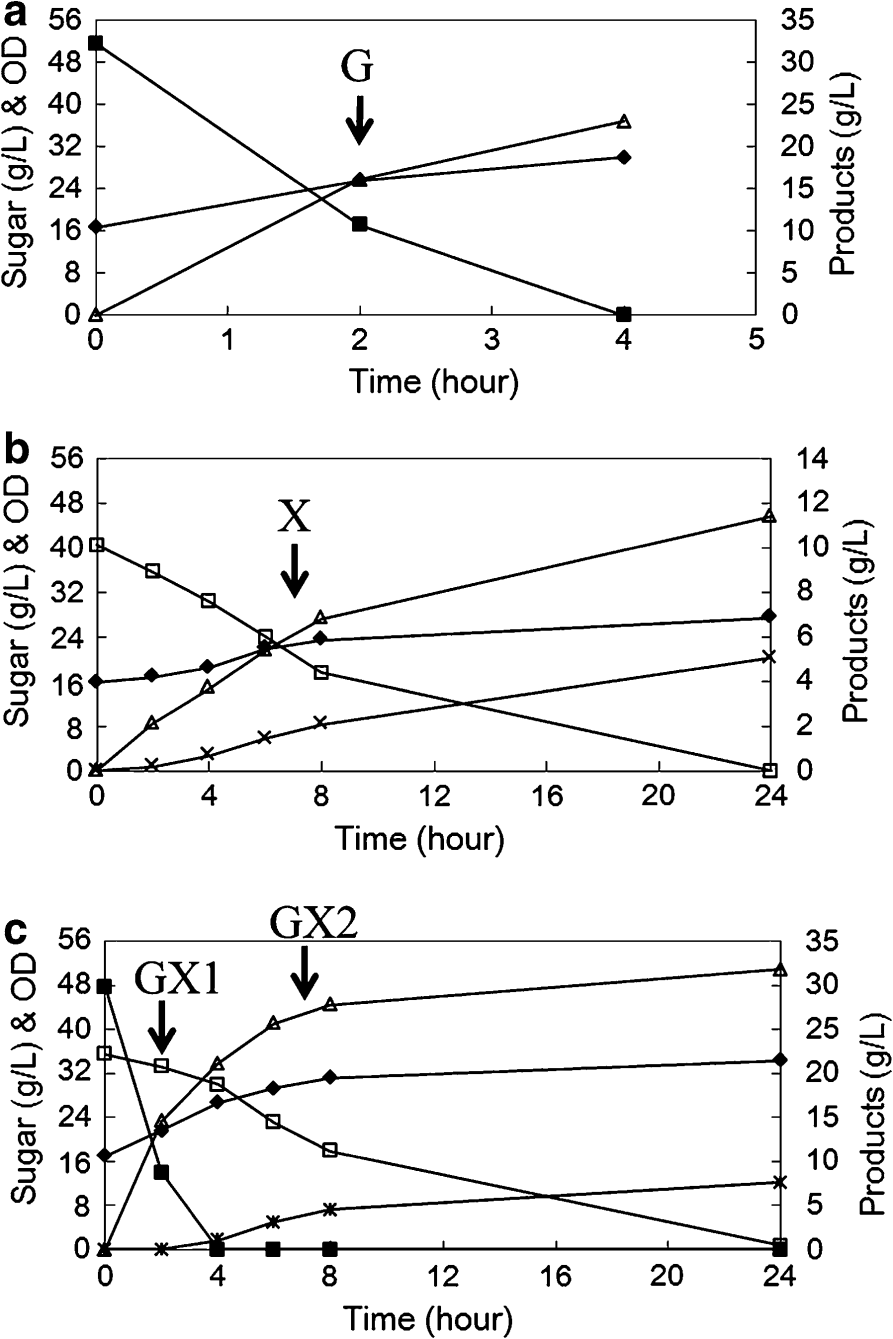
Time-dependent fermentation profile of KF7M-16 in **a** YPD medium containing 60 g/L glucose (6 % YPD), **b** YPX medium containing 40 g/L xylose (4 % YPX), and **c** YPDX medium containing 60 g/L glucose and 40 g/L xylose (10 %YPDX). *Black diamond* OD_660_; *black square* glucose; *empty square* xylose; *empty up*-*pointing triangle* ethanol; and *error marks* xylitol. The *arrows* (denoted by G, X, GX1, and GX2) indicate the times at which samples were taken for transcription analysis

Batch fermentations were carried out in YP medium containing 60 g/L glucose (6 % YPD) or 40 g/L xylose (4 % YPX) as single sugar medium, as well as in YP medium containing 60 g/L glucose and 40 g/L xylose as mixed sugar medium (10 % YPDX). To eliminate the effect of different growth rates between glucose and xylose utilization on the transcriptional profile, a relatively high inoculum density of 5 g-DCW/L was chosen. A pre-experiment was conducted to grasp the fermentation profile of KF7M-16 (Fig. 1). Glucose was depleted in the first 4 h of fermentation, both in 6 % YPD and 10 % YPDX. The utilization rate of xylose was approximately a magnitude slower than that of glucose (2.88 g/L/h in 4 % YPX in the first 8 h, 17.27 g/L/h in 6 % YPD in the first 2 h and 16.80 g/L/h in 10 % YPDX in the first 2 h), and after 24 h of metabolism, KF7 M-16 almost consumed all of the xylose in both the 4 % YPX and 10 % YPDX. It is worth noting that the presence of glucose repressed the utilization of xylose, as in 10 % YPDX the xylose consumption rate was 1.38 g/L/h in the first 4 h and 3.03 g/L/h in 4–8 h. Therefore the fermentation profile in 10 % YPDX can be divided into the glucose fermentation phase and the xylose fermentation phase.

For transcription analysis, experiments were conducted in duplicate. Samples of glucose-grown cells were harvested at 2 h while samples of xylose-grown cells were harvested at 7 h. The samples taken at 2 h in 6 % YPD were denoted as G, and the residual glucose was 16.4 ± 1.2 g/L, with 16.4 ± 0.3 g/L ethanol produced at a yield of 0.46 ± 0.01 g/g at that time point. The samples taken at 7 h in 4 % YPX were denoted as X, and at that time point the residual xylose was 20.4 ± 0.1 g/L, with 6.2 g/L ethanol and 1.7 ± 0.1 g/L xylitol produced at yields of 0.35 ± 0.1 and 0.10 ± 0.003 g/g respectively. The samples taken at 2 h in 10 % YPDX (during glucose fermentation stage) were denoted as GX1, and during this first 2 h of fermentation, 37.1 ± 5.1 g/L glucose and 2.3 ± 1.3 g/L xylose were consumed, producing 17.2 ± 1.8 g/L ethanol at a yield of 0.44 ± 0.02 g/g. The samples taken at 7 h in 10 % YPDX (during xylose fermentation stage) were denoted as GX2, and the glucose had been depleted for at least 3 h by then, and the residual xylose was 19.5 ± 1.0 g/L, with 27.8 ± 1.8 g/L ethanol as well as 4.6 ± 0.3 g/L xylitol produced at yields of 0.38 and 0.26 ± 0.04 g/g respectively. It’s notable that the ethanol yield from xylose was 24 % lower than that from glucose, and that the xylitol yield from xylose was 160 % higher in the mixed sugar medium than in the single sugar medium.

### Overview of transcriptional differences across four conditions

**Fig. 2:**
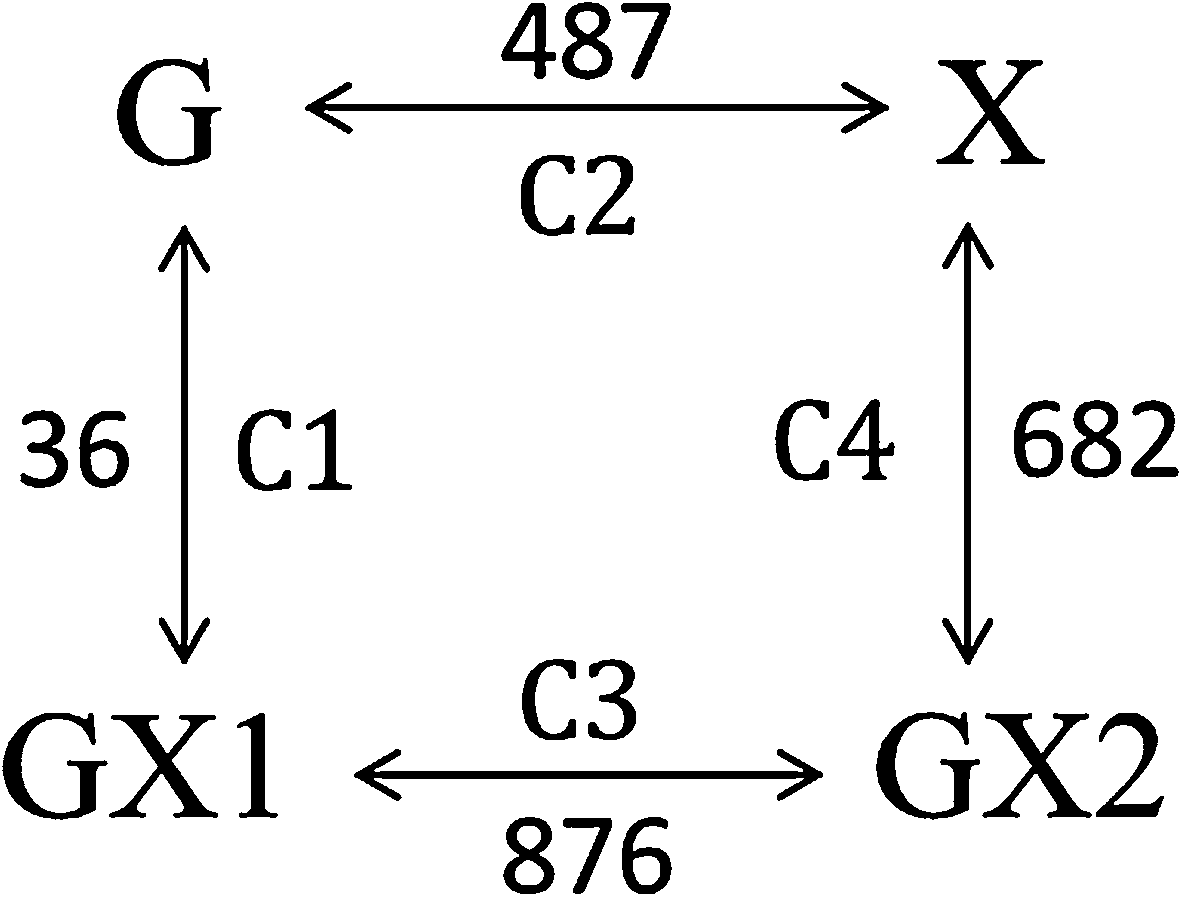
Overview of differently expressed gene numbers between different fermentation conditions. *G* glucose fermentation state in the glucose alone medium; *X* xylose fermentation state in the xylose alone medium; *GX1* glucose fermentation phase in the mixed sugar; *GX2* xylose fermentation phase in the mixed sugar. C1–C4 indicate the specific pairwise comparisons. Averages of biological duplicates were compared. The *numbers* indicate differently expressed genes in each comparison

To obtain the general information of transcriptional responses to the different sugar sources, the microarray data was organized into four relevant pairwise comparisons: GX1 vs. G (comparison 1 = C1); X vs. G (comparison 2 = C2); GX2 vs. GX1 (comparison 3 = C3); and GX2 vs. X (comparison 4 = C4). The averages of biological duplicates were compared. The numbers of genes with significantly changed expression levels (fold change ≥2) between the corresponding conditions are shown in Fig. 2. Differentially expressed genes can be identified and enriched for pathway terms using the KEGG Orthology Based Annotation System (KOBAS).

#### GX1 vs. G

Only 36 genes stood out as being differently expressed in C1 (Additional file 1: Table S2), with 16 upregulated and 20 downregulated genes. This suggested that the transcriptional profile of cells during glucose fermentation phase in the mixed sugar medium was nearly identical to those growing in glucose alone medium. Since xylose is not a natural carbon source for *S. cerevisiae*, recombinant strains have a stronger preference for glucose rather than xylose. KF7M-16 utilized glucose first in the mixed sugar medium. Although a meager amount of xylose was consumed in the first 2 h of fermentation, the added xylose seemed powerless to affect the glucose dominated transcriptional profile.

#### X vs. G

To reveal the molecular basis for the fermentation ability discrepancy between glucose and xylose, the transcriptional difference in *S. cerevisiae* growing in either the glucose alone or the xylose alone medium has long been analyzed, both aerobically and anaerobically. In our study with the low aeration condition, a total of 487 genes showed significantly different expression levels (fold change ≥2) in C2, with 281 upregulated and 206 downregulated genes (Additional file 1: Table S3). The significantly enriched pathway terms (*p* value < 0.1) are listed in Table 1.

**Table 1:** Enriched pathways of the differently expressed genes

Comparison	Term	Database	ID	*p* value
C2	Biosynthesis of secondary metabolites	KEGG PATHWAY	sce01110	2.21E−05
	Carbon metabolism	KEGG PATHWAY	sce01200	2.46E−04
	Histidine biosynthesis	PANTHER	P02747	5.18E−04
	Glyoxylate and dicarboxylate metabolism	KEGG PATHWAY	sce00630	6.25E−04
	Biosynthesis of amino acids	KEGG PATHWAY	sce01230	1.09E−03
	Metabolic pathways	KEGG PATHWAY	sce01100	2.96E−03
	Sulfur metabolism	KEGG PATHWAY	sce00920	3.70E−03
	Citrate cycle (TCA cycle)	KEGG PATHWAY	sce00020	5.86E−03
C3	Biosynthesis of secondary metabolites	KEGG PATHWAY	sce01110	4.19E−03
	Heme biosynthesis	PANTHER	P02746	9.02E−03
	Histidine metabolism	KEGG PATHWAY	sce00340	1.20E−02
	Tryptophan metabolism	KEGG PATHWAY	sce00380	1.42E−02
	Porphyrin and chlorophyll metabolism	KEGG PATHWAY	sce00860	1.42E−02
C4	Sulfur metabolism	KEGG PATHWAY	sce00920	7.11E−04
	superpathway of sulfur amino acid biosynthesis	BioCyc	PWY-821	1.44E−03
	Arginine biosynthesis	PANTHER	P02728	1.58E−03
	Sulfate reduction I (assimilatory)	BioCyc	SO4ASSIM-PWY	9.14E−03

http://bioinfo.capitalbio.com/mas3/

Secondary metabolites are organic compounds that are not directly involved in the normal growth or reproduction of the cells, and often have a role in stress defense. A secondary metabolite forms frequently near or in the stationary phase of growth. The KEGG term of “Biosynthesis of secondary metabolites” (ID: sce01110) involves a number of pathways, including carbon metabolism and biosynthesis of amino acids.

The expressional changes in carbon metabolism directly reflect the different metabolic states in different sugar sources. Genes involved in the citrate and glyoxylate cycles, which are repressible by glucose (Gancedo 1998), were upregulated in the xylose alone medium, indicating that xylose lacked the carbon catabolite repression capability. Genes involved in the central carbon metabolism across the four conditions will be further discussed later.

The expression levels of the genes involved in sulfur metabolism were downregulated in C2. The detailed transcriptional changes will be further discussed in C4.

#### GX2 vs. GX1

The transcription profiles in the mixed sugar medium were compared between the xylose fermentation stage and the glucose fermentation stage. A total of 499 and 377 genes were upregulated and downregulated respectively in C3 (Additional file 1: Table S4). The number of differently expressed genes in C3 was 80 % more than in C2, although both comparisons were carried out between glucose fermentation state and xylose fermentation state. The transcriptional shift induced by different sugar source utilization was more complex in the mixed sugar medium, mainly due to the prolonged effect of glucose repression and the stress induced by the accumulated ethanol. The enriched pathway terms (*p* < 0.02) are listed in Table 1.

Similarly to C2, the KEGG term “biosynthesis of secondary metabolites” was enriched with the lowest *p* value in C3.

Heme is a cofactor consisting of a ferrous ion contained in the center of a large heterocyclic organic ring called a porphyrin. Heme is the component of cytochrome which is primarily responsible for the generation of ATP via electron transport. Although the expression level of genes encoding respirative enzymes increased during the xylose fermentation stage relative to the glucose fermentation stage in the mixed sugar medium (Table 4), several genes involved in the heme biosynthesis from uroporphyrinogen (*HEM12*, *HEM13*, *HEM15*) showed a decreased expression level. Whereas the genes involved in the siroheme biosynthesis from uroporphyrinogen (*MET1*, *MET8*) showed an increased expression level.

It has been previously suggested that the increased expression level of the genes related to tryptophan biosynthesis might confer ethanol stress tolerance to yeast cells (Hirasawa et al. 2007). In our study, the expression level of the genes related to tryptophan degradation (*BNA1, BNA4, BNA7, ARO8*) decreased when the transcriptional profile of xylose fermentation stage in the mixed sugar medium was compared to that of the glucose fermentation stage, corroborating the involvement of tryptophan in response to ethanol stress.

Genes involved in histidine biosynthesis (*HIS1*, *HIS7*, *HIS2*), which were previously reported to be significantly downregulated in the presence of ethanol (Li et al. 2010), also had a decreased expression level in C3.

#### GX2 vs. X

Since the metabolic activity during the xylose fermentation phase in mixed sugar medium was similar to that in xylose alone medium, it is interesting to observe that when the transcription profile of GX2 was compared to that of X, 682 genes were differently expressed, with 375 upregulated and 307 downregulated genes (Table S5, in the supplementary material). Significantly enriched pathway terms (*p* < 0.01) are listed in Table 1.

**Fig. 3:**
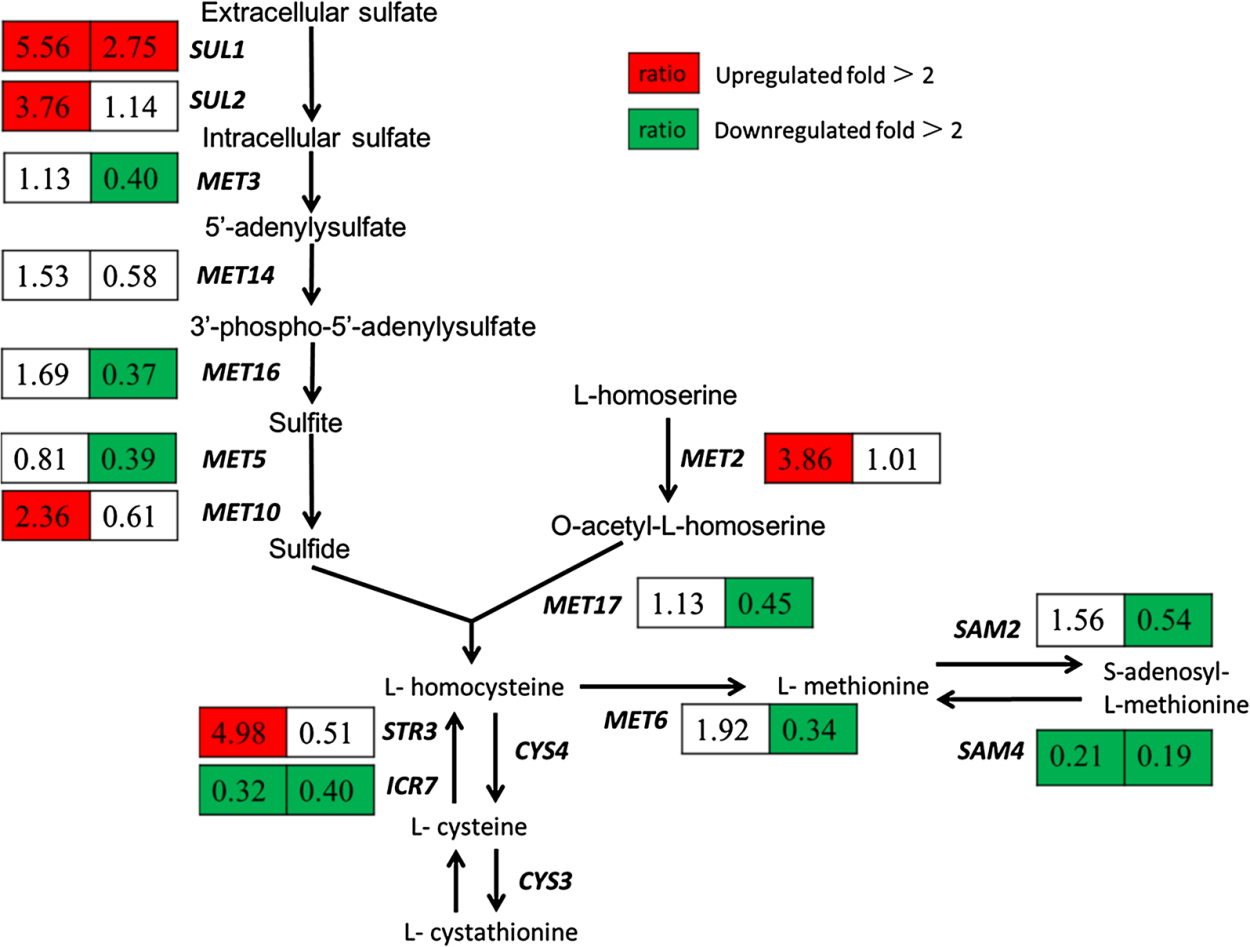
The expression ratio of the genes involved in sulfur amino acid biosynthesis. The fold change of expression is presented for GX2 (*left panel*) and X (*right panel*), both compared to the transcriptional state in the glucose alone medium (G) as a control. Averages of biological duplicates were compared. The *red panel* indicates that the transcription level of gene increased over twofold; the *green panel* indicates the transcription level of gene decreased over twofold

Sulfur amino acid biosynthesis can be divided into three parts: sulfate assimilation, cysteine and methionine biosynthesis, and S-adenosyl-l-methionine (SAM) biosynthesis (Thomas and SurdinKerjan 1997). SAM is probably second only to ATP in the variety of reactions for which it serves as a cofactor, and is also a precursor of glutathione, which is a major cellular anti-oxidant (Caro and Cederbaum 2004). Compared to their expression levels in the glucose alone medium as a control, the expression of genes involved in sulfur amino biosynthesis was higher in GX2 and was lower in X. The comparative expression ratios are presented in Fig. 3. It is reasonable to speculate that the demand for SAM might be higher during the xylose fermentation stage in the mixed sugar medium than in xylose alone medium. Considering that SAM is protective against a variety of toxic oxidative agents, this difference might be due to the metabolites accumulated in the process of glucose fermentation.

The expression of genes involved in arginine biosynthesis from glutamine (*CPA2*, *ARG3*, *ARG1*, and *ARG4*) also increased in C4.

### Carbon source sensing and signaling

**Fig. 4:**
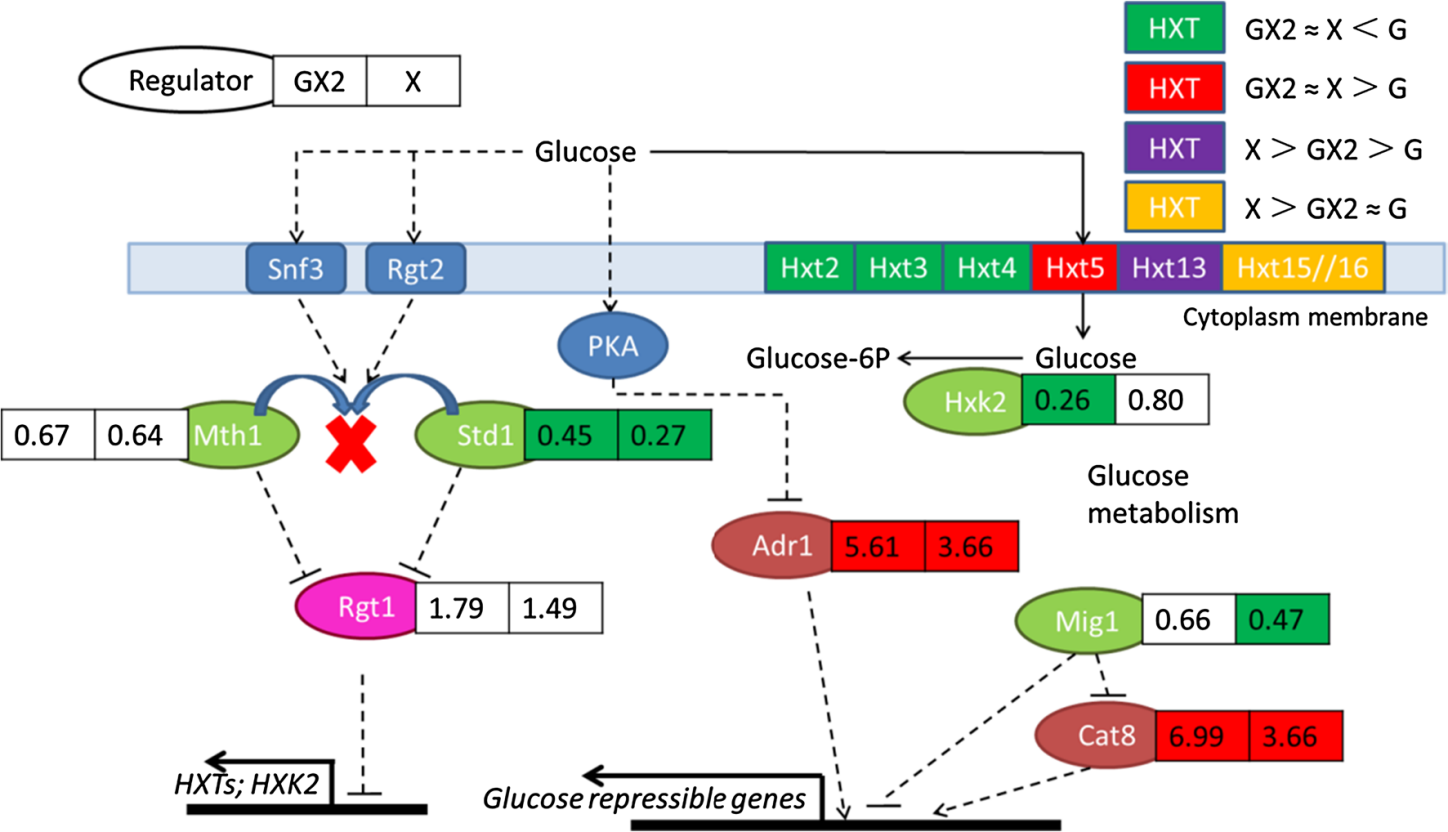
Expression profiles of the hexose transporter genes and comparison of the transcriptional changes in the genes involved in glucose sensing and repression network. The relative expression of hexose transporter genes is indicated with *different colors*. For the regulator genes, the fold change of expression is presented for GX2 (*left panel*) and X (*right panel*), both compared to G as a control. Averages of biological duplicates were compared. The *red panel* indicates the transcription level of gene increased over twofold; the *green panel* indicates the transcription level of gene decreased over twofold

Uptake of xylose by *S. cerevisiae* has been proposed to be mediated unspecifically by its hexose-transport system, which is composed of 18 genes from the *HXT*s family and additional broad substrate sugar transporters (Hamacher et al. 2002). The genes encoding transporters showed different transcriptional patterns on different sugar sources (Fig. 4, Table 2). The transcriptional levels of the high-affinity glucose transporter genes *HXT2* and *HXT4*, as well as the low-affinity glucose transporter gene *HXT3* were downregulated with xylose, in both the xylose alone medium and during the xylose fermentation phase in the mixed sugar medium. On the other hand, the non-fermentable carbon source-inducible transporter genes *HXT5* and *HXT13* were upregulated with xylose, indicating that xylose was not recognized by *S. cerevisiae* as being fermentable. *HXT15* and *HXT16*, repressed by high levels of glucose, were induced nearly 40 times in the xylose alone medium, but not during the xylose fermentation phase in the mixed sugar medium, suggesting that the effect of glucose repression could last after glucose had been depleted. *HXT6//7* were expressed at high levels under all four conditions. Other genes in the HXTs family did not show obvious differences in expression level, and all of them were negligibly expressed, with the exception of *HXT9*. As deduced from mRNA level of different transporters (Table 2), *HXT3* and *HXT4*, together with *HXT6* and *HXT7* might serve as the main transporters in the glucose fermentation state, while *HXT6*, *HXT7* as well as *HXT4* might be the main transporters in the xylose fermentation state.

**Table 2:** The hybridization signal (MAS5.0 signal intensity) of the genes encoding hexose-transport system in different fermentation states

Gene	G	GX1	GX2	X
*HXT1*	25.9 ± 2.9	29.7 ± 11.5	38.8 ± 15.8	24.3 ± 1.9
*HXT2*	700.8 ± 76.9	500.1 ± 236.3	125.4 ± 13.0	114.8 ± 47.5
*HXT3*	3937.3 ± 299.5	3039.1 ± 828.4	643.8 ± 235.8	555.2 ± 106.5
*HXT4*	3542.4 ± 626.0	3868.6 ± 17.7	1389.3 ± 118.5	1136.8 ± 207.0
*HXT5*	34.6 ± 1.2	48.9 ± 30.0	220.1 ± 80.3	142.9 ± 59.3
*HXT6//7* ^a^	1756.3 ± 302.6	1801.2 ± 181.3	2031.2 ± 157.8	1949.0 ± 7.5
*HXT8*	24.9 ± 0.2	23.2 ± 0.7	22.3 ± 0.9	22.6 ± 0.6
*HXT9*	263.5 ± 56.7	256.0 ± 50.2	242.6 ± 9.4	190.3 ± 8.7
*HXT10*	7.9 ± 0.2	7.6 ± 0.2	7.0 ± 0.1	8.0 ± 0.2
*HXT11*	8.1 ± 0.2	5.8 ± 0.8	7.9 ± 1.5	8.4 ± 1.1
*HXT13*	17.0 ± 6.9	13.6 ± 0.6	39.8 ± 1.6	131.3 ± 19.3
*HXT14*	7.9 ± 0.1	7.9 ± 0.3	11.7 ± 0.8	7.7 ± 0.3
*HXT15//16* ^a^	12.0 ± 1.1	13.2 ± 0.9	14.5 ± 0.9	536.4 ± 110.5
*GAL2*	38.6 ± 0.3	50.1 ± 6.9	79.2 ± 5.1	58.3 ± 2.7

Values are given as the average and standard deviation of two biological duplicates

^a^“//” indicates genes detected by the same probe set because of their sequence similarity

It has been previously reported that genes encoding the glucose sensor proteins Snf3p and Rgt2p, which mediate signal for the presence of glucose at low or high concentration respectively, had higher expression levels with xylose (Salusjärvi et al. 2008). In our study, *SNF3* and *RGT2* had stable expression levels across all four conditions. Glucose binds to Snf3 and Rgt2, inducing them to bind to Mth1 and Std1, and finally leading to the degradation of Mth1 and Std1, which are two Rgt1corepressors (Zaman et al. 2008). The expression levels of the corepressor genes *MTH1* and *STD1* decreased with xylose, and that of *RGT1* slightly increased (Fig. 4). Rgt1 is a DNA-binding protein that represses the hexose transporter genes *HXT2*-*4*, as well as hexokinase gene *HXK2*. The expression levels of these genes were correspondingly downregulated. The xylose-dependent upregulation of *RGT1*, irrespective of oxygen availability, has been previously observed (Alff-Tuomala et al. 2016). The expression levels of the transcription factor Adr1, which is negatively regulated by PKA in glucose growing cells (Zaman et al. 2008), were upregulated with xylose. Glucose metabolism activates the transcriptional repressor Mig1 to move into the nucleus and repress its many targets (Kuttykrishnan et al. 2010). The expression level of Mig1 decreased with xylose, while that of Cat8, which is repressed by Mig1, increased with xylose. Transcription factor Cat8 activates gene expression required for gluconeogenesis during growth in the absence of glucose, the transcriptional changes of which will be discussed in the following section.

### Central carbon metabolism

**Fig. 5:**
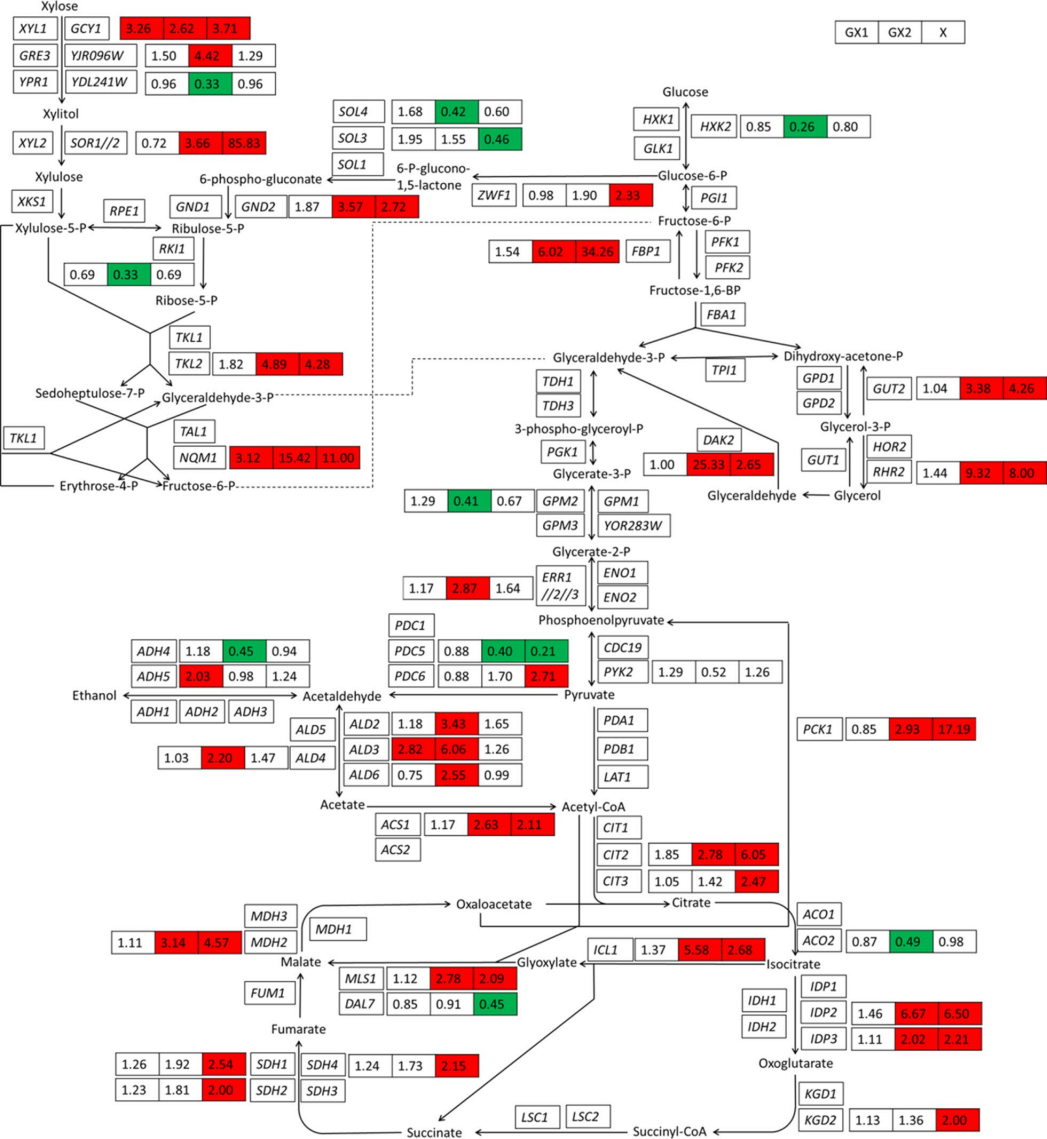
Expression profiles of genes involved in the central carbon metabolism. The fold change of expression is presented for GX1 (*left panel*), GX2 (*middle panel*) and X (*right panel*), compared to G as a control. Averages of biological duplicates were compared. The *red panel* indicates the transcription level of gene increased over twofold; the *green panel* indicates the transcription level of gene decreased over twofold

Xylose was channeled to ethanol via the heterologous xylose assimilating pathway, pentose phosphate pathway, glycolysis pathway and alcohol fermentation pathway. To pinpoint the effect of xylose on the fermentation state, the genes expression in the central carbon metabolism was investigated in greater detail, using the transcriptional state when cultivated in glucose alone medium as a control (Fig. 5).

Since the heterologous *XYL1* and *XYL2* genes from *S. stipitis* were not included in the *S. cerevisiae* genechips, their expression levels were determined by RT-qPCR (Table 3). The heterologous *XYL1* and *XYL2* did not show significant difference across all the tested conditions, but the *S. cerevisiae* genes encoding enzymes with xylose reductase and xylitol dehydrogenase function manifested different expression patterns (Fig. 5). The expression level of *GCY1* increased as long as the medium contained xylose. Another transcription study using mixed sugar medium also indicated expression level of *GCY1* increased even in glucose fermentation phase (Alff-Tuomala et al. 2016). During the xylose fermentation phase in the mixed sugar medium, the expression level of *YJR096W* increased and that of *YDL241W* decreased, while both genes did not express differently in xylose alone medium, compared to glucose alone medium, and both have been previously reported to be poor aldo–keto reductases (Chang et al. 2007). The expression levels of the endogenous genes *SOR1* and *SOR2* increased during xylose fermentation, and to a much stronger extent in the xylose alone medium than in the mixed medium, which might have accounted for the lower observed xylitol yield in the xylose alone medium than in the mixed medium. For *SOR1//2*, the microarray data was validated by RT-qPCR (Table 3). Even though the *SOR1//2* expression level increased in magnitudes in X as compared to glucose fermentation state (G and GX1), the transcript abundance of *SOR1//2* was still lower than *XYL2* under xylose fermentation state.

**Table 3:** Expression level of xylose metabolizing genes by RT-qPCR

Sugar	*XYL1/ACT1*	*XYL2/ACT1*	*SOR1//2/ACT1*
G	1.9 ± 0.25	2.53 ± 0.52	7.35E−5 ± 1.2E−6
X	1.9 ± 0.42	2.36 ± 0.58	1.13E−1 ± 2.1E−2
GX1	2.0 ± 0.22	2.17 ± 0.34	6.84E−5 ± 3.3E−6
GX2	2.19 ± 0.45	1.28 ± 0.28	1.08E−3 ± 5.7E−5

Values are given as the average and standard deviation of two duplicates

The major enzymes in the non-oxidative pentose phosphate pathway, *TAL1* and *TKL1* encoding transaldolase and transketolase (Matsushika et al. 2012), did not express significantly differently across all conditions, but the expression of their minor isoenzyme *NQM1* and *TKL2* was induced during xylose fermentation. The expression level of *NQM1* is usually induced during diauxic shift and *TKL2* is regulated in a Msn2/4p manner. Another non-oxidative PPP gene, *RKI1*, was expressed at a lower level on xylose. In the oxidative pentose phosphate pathway, the expression levels of *ZWF1* encoding glucose-6-phosphate dehydrogenase and *GND2* encoding 6-phosphogluconate dehydrogenase increased on xylose.

In three structural genes encoding enzymes that catalyze the phosphorylation of glucose to glucose 6-phosphate, *HXK2* was repressed with xylose, especially during the xylose fermentation phase in the mixed sugar medium. Hxk2p is known to provide the main sugar-phosphorylating capability during growth on fermentable carbon source, and becomes repressed on non-fermentable carbon source by transcription factor Rgt1 (Palomino et al. 2005).

Most glycolysis genes showed stable expression levels across all four conditions, except for two genes encoding homolog of Gpm1p phosphoglycerate mutase, *GPM2* and *GPM3*, were slightly downregulated on xylose, and these two genes may be non-functional.

In the alcohol fermentation and by-product pathway, two genes encoding the minor isoform of pyruvate decarboxylase, *PDC5* and *PDC6*, respectively, showed decreased and increased expression levels with xylose. *PDC5* is repressed by thiamine, and *PDC6* is induced during sulfur limitation. Yet several genes involved in thiamine biosynthesis (*THI* genes) showed decreased expression levels with xylose. Most *ALD* genes involved in acetate formation showed increased expression level during the xylose fermentation stage in the mixed sugar medium, including cytosolic aldehyde dehydrogenase *ALD2*, *ALD3*, *ALD6*, and mitochondrial aldehyde dehydrogenase *ALD4*. This upregulation was likely to be a response to the higher ethanol level at the sampling time of GX2. The expression level of *ACS1*, encoding a synthetase to form acetyl coenzyme A from acetate, increased with xylose, once again indicating that xylose is sensed as a non-fermentable carbon source by *S. cerevisiae.*

The expression levels of several genes in the tricarboxylic acid cycles were upregulated with xylose. Transcripts of *CIT2* and *CIT3,* encoding enzymes that catalyze the condensation of acetyl coenzyme A and oxaloacetate to form citrate, increased with xylose, especially in the xylose alone medium. The levels of *ACO2*, encoding a putative mitochondrial aconitase and repressed by ethanol, decreased during the xylose fermentation stage in the mixed sugar medium. As for the *IDP* genes that encode isocitrate dehydrogenase, the expression level of *IDP1* encoding mitochondrial isocitrate dehydrogenase did not change, but that of *IDP2* and *IDP3*, encoding NADP^+^ specific cytosolic and peroxisomal isocitrate dehydrogenase respectively, increased with xylose. The *KGD* genes (*KGD1* and *KGD2*) encoding components of the mitochondrial alpha-ketoglutarate dehydrogenase complex, as well as the *SDH* genes (*SDH1, SDH2, SDH3,* and *SDH4*) that encode subunits of succinate dehydrogenase, had slightly increased expression levels with xylose. The expression level of *MDH2*, encoding cytoplasmic malate dehydrogenase, which is also involved in the gluconeogenesis and glyoxylate cycles, increased with xylose. Because 2 carbons lost as CO_2_ in one TCA cycle, the strong activity of the TCA cycle could be the reason for the lowered ethanol yield from xylose. Corroborating with the upregulated TCA cycle pathway, the expression levels of a number of genes encoding respiratory enzymes increased with xylose (Table 4). The expression level of *HAP4*, a transcriptional activator and global regulator of respiratory gene expression, also slightly increased with xylose. As a result, a significant amount of ATP was assumed to be produced in xylose metabolism, indicating high requirements for maintenance energy to utilize xylose by recombinant *S. cerevisiae*.

**Table 4:** The expressional fold changes of the genes responsible for respiratory metabolism and ATP synthesis for GX1, GX2, and X, compared to G as a control (averages of biological duplicates were compared)

Genes	GX1	GX2	X	Description
*HAP4*	1.11	1.55	2.13	Transcriptional activator of respiratory genes
*NDE1*	0.82	5.54	5.65	Mitochondrial external NADH dehydrogenase
*NDE2*	1.29	2.92	3.39	
*NDI1*	1.21	2.55	2.27	Ubiquinone oxidoreductase
*QCR2*	1.14	1.99	2.12	Subunits of ubiquinol cytochrome *c* reductase complex
*QCR9*	1.18	2.38	1.67	
*QCR8*	1.10	2.07	1.49	
*QCR10*	1.24	2.06	1.87	
*CYC3*	0.88	1.80	2.01	Cytochrome c heme lyase
*CYC7*	1.18	3.49	1.63	Cytochrome c isoform 2
*COX5A*	0.99	2.56	1.96	Subunits of cytochrome *c* oxidase
*COX7*	1.20	2.78	2.30	
*COX12*	1.00	1.92	2.09	
*COX13*	0.96	2.81	1.96	
*ATP2*	1.05	1.15	1.91	Subunits of ATP synthase
*ATP3*	1.06	1.38	1.63	
*ATP4*	1.15	1.39	1.78	
*ATP5*	1.05	0.93	1.62	
*ATP14*	1.06	1.27	1.62	
*ATP16*	1.10	1.34	1.67	
*ATP18*	1.10	1.20	1.78	

The gluconeogenic gene *FBP1*, encoding fructose-1,6-bisphosphatase, was strongly induced with xylose. The transcription levels of other genes involved in gluconeogenesis, such as *PCK1* encoding phosphoenol-pyruvate carboxykinase and *MDH2*, also increased with xylose. The upregulation ratio was higher in the xylose alone medium than during the xylose fermentation phase in the mixed sugar medium. The gluconeogenesis pathway is repressed by glucose, and our results indicated that this pathway was not fully derepressed after glucose had been depleted. The expression levels of the specific genes in the glyoxylate shunt, *ICL1* and *MLS*1 encoding isocitrate lyase and malate synthase respectively, together with *CIT2* encoding peroxisomal citrate synthase were also induced on xylose.

In the glycerol catabolism pathway, the expression level of the glycerol-producing gene *RHR2* encoding glycerol-1-phosphatase increased with xylose, which is contrary to an earlier finding with anaerobic batch fermentation (Matsushika et al. 2014). The expression levels of glycerol-consuming genes, *GUT2* encoding mitochondrial glycerol-3-phosphate dehydrogenase and *DAK2* encoding dihydroxyacetone kinase, also increased with xylose. *DAK2* was strongly induced during the xylose fermentation stage in the mixed sugar medium, likely due to its involvement in stress adaptation. Similar to *DAK2*, *STL1* encoding the glycerol proton symporter of the plasma membrane was also strongly induced during the xylose fermentation stage in the mixed sugar medium, but not in the xylose alone medium. *STL1* was the gene with the largest upregulation ratio (70.90) in C4, reflecting the different osmotic state between GX2 and X, because *STL1* is documented to be strongly but transiently induced when cells are subjected to osmotic shock.

## Discussion

The transcription profiles of an industrial recombinant *S. cerevisiae* strain was compared between different fermentation states of glucose and xylose both in single sugar and mixed sugar media. The presence of glucose repressed the utilization of xylose, and it is found that the transcriptome of the glucose fermentation phase in the mixed-sugar medium was very similar to that in the glucose alone medium. A recently published gene expression study using mixed sugar medium found that respiratory genes were not fully repressed when xylose was present with abundant glucose (Alff-Tuomala et al. 2016), which was not observed in our study. On the other hand, we found that although the transcriptome highly differed between the xylose metabolizing cells cultured in the xylose alone medium and those in the xylose fermentation phase of the mixed sugar medium, the xylose consumption rate was nearly identical under these two conditions. It appears that *S. cerevisiae* has adequately evolved mechanisms to respond to metabolites accumulated in the process of glucose fermentation.

The main challenge in the recombinant strains expressing *S. stipitis* XR-XDH pathway is redox imbalance, because xylose reductase prefers NADPH, whereas xylitol dehydrogenase strictly utilizes NAD^+^, leading to the accumulation of NADP^+^ and NADH. NADPH is regenerated in *S. cerevisiae* mainly through the oxidative branch of the pentose phosphate pathway and the cytosolic and peroxisomal isocitrate dehydrogenases. We found that the expression levels of *ZWF1* and *GND2* in oxidative PPP, and that of *IDP2* and *IDP3* were upregulated on xylose (Fig. 5), which corroborated with earlier reports either in aerobic or anaerobic conditions (Matsushika et al. 2014; Salusjärvi et al. 2008). It is known that the ratio of NADP^+^/NADPH directly influence the activity of glucose-6-phosphate dehydrogenase, while the dehydrogenation of glucose-6-phosphate catalyzed by *ZWF1* is the rate-limiting reaction in PPP. When one mole of ribulose-5-P is produced from glucose-6-phosphate through oxidative PPP generating two moles of NADPH, one carbon is lost as CO_2_, leading to the poor use of carbon substrate.

Meanwhile, the gluconeogenesis pathway was upregulated to feed glucose-6-phosphate to oxidative PPP, consistent with an earlier report (Runquist et al. 2009). In our study, differences in the expression level were slight for *PYC1* and *PYC2,* which encode cytoplasmic pyruvate carboxylase that converts pyruvate to oxaloacetate in the first step of gluconeogenesis. However, cytoplasmic oxaloacetate can also be produced from acetyl-coA through the glyoxylate cycle, which was upregulated on xylose (Fig. 4). The expression levels of *PCK1* encoding phosphoenolpyruvate carboxykinase that catalyzes the second step of gluconeogenesis were increased, and to a significantly larger extent in the xylose alone medium than in the xylose fermentation phase in the mixed-sugar medium. The generation of phosphoenolpyruvate from oxaloacetate is accompanied by a loss of 1 carbon as CO_2_, and consumption of 1 ATP. The upregulation ratio of *FBP1* was consistent to that of *PCK1*.

As to the regeneration of NAD^+^, since alcoholic fermentation itself is a redox-neutral process (the NADH reduced in ethanol formation accounts for the NADH generated in the glyceraldehyde-3-phosphate dehydrogenase reaction), the accumulated NADH must be otherwise reoxidized. Despite the impermeability of the inner mitochondrial membrane for NADH and NAD^+^, both cytosolic and mitochondrial NADH can be reoxidized by the respiratory chain in *S. cerevisiae*. The cytosolic NAD^+^ can be generated via mitochondrial external NADH hydrogenases (Bakker et al. 2001), encoded by *NDE1* and *NDE2*. Alternatively, cytosolic NADH can be reoxidized by the respiratory chain via the glycerol- 3-phosphate shuttle consisting of cytosolic NADH-linked glycerol-3-phosphate dehydrogenase and mitochondrial FAD linked glycerol-3-phosphate dehydrogenase encoded by *GUT2* that transfers electrons from cytosolic glycerol- 3-phosphate to ubiquinone. The expression level of *NDE1*, *NDE2* and *GUT2* increased significantly with xylose in our study, both in the xylose alone medium and during the xylose fermentation phase in the mixed sugar medium (Table 4 and Fig. 5). The expression levels of many others genes encoding respiratory enzymes such as cytochrome *c* reductase that transfers electrons from ubiquinone to cytochrome *c* and cytochrome *c* oxidase, which catalyzes the oxidation of cytochrome *c* by molecular oxygen, also increased with xylose (Table 4).

It is assumed that xylose is poorly metabolized to ethanol because it does not repress respiration in the same manner as glucose (Jin et al. 2004). *S. cerevisiae* growing in glucose exhibits the Crabtree effect, where respiratory growth can only be achieved with a limited sugar supply and at low specific growth rate. It is possible that the lack of ability to repress respiration by xylose-metabolizing cells is the consequence of a low level of xylose uptake by *S. cerevisiae*. However, it has been previously reported that the overexpression of xylose-transporting protein did not enhance xylose fermentation (Hamacher et al. 2002). It is then possible that the upregulation of respiration is driven by the demand for ATP and NAD^+^. However, the metabolic flux analysis of a xylose isomerase-expressing strain, which does not face the NADH accumulation challenge during xylose assimilation, also indicated that xylose failed to elicit the full carbon catabolite repression response (Wasylenko and Stephanopoulos 2015). As for ATP metabolism, since ADP activates the Snf1 regulator by protecting it against dephosphorylation (Mayer et al. 2011), it is tempting to infer, from the increased Snf1-activated transcription factors Adr1 and Cat8 expression levels as well as the decreased Snf1-deactivated Mig1 expression levels (Fig. 4), that the intracellular ADP level increased in xylose metabolizing cells. Coincidentally, high requirements for maintenance energy by the recombinant xylose metabolizing *S. cerevisiae* was found in a metabolic analysis using XR-XDH strains (Feng and Zhao 2013b). Meanwhile, the TCA cycle and respiratory enzymes were found to be upregulated even under anaerobic xylose fermenting conditions where no ATP can be generated by oxidative phosphorylation due to the absence of oxygen (Matsushika et al. 2014). Taken together, we propose that it is crucial to reduce the maintenance energy of the xylose metabolizing strains to improve xylose utilization. Moreover, the regulatory network for xylose fermentation appeared sub-optimal, as futile induction could occur. The regulatory network could alternatively be refigured by transcription factor engineering or adaptive evolution.

## Additional file


10.1186/s13568-016-0223-y Supplementary material.

## Abbreviations

XR: xylose reductase
XDH: xylitol dehydrogenase
XI: xylose isomerase
YPX: yeast extract peptone xylose
YPDX: yeast extract peptone dextrose xylose
SAM: *S*-adenosylmethionine
TCA: tricarboxylic acid
GEO: gene expression omnibus

## References

[CR1] Alff-Tuomala S, Salusjärvi L, Barth D, Oja M, Penttilä M, Pitkänen JP, Ruohonen L, Jouhten P (2016). Xylose-induced dynamic effects on metabolism and gene expression in engineered *Saccharomyces cerevisiae* in anaerobic glucose-xylose cultures. Appl Microbiol Biotechnol.

[CR2] Bakker BM, Overkamp KM, Maris AJAV, Kötter P, Luttik MAH, Dijken JPV, Pronk JT (2001). Stoichiometry and compartmentation of NADH metabolism in *Saccharomyces cerevisiae*. FEMS Microbiol Rev.

[CR3] Batt CA, Carvallo S, Easson DD, Akedo M, Sinskey AJ (1986). Direct evidence for a xylose metabolic pathway in *Saccharomyces cerevisiae*. Biotechnol Bioeng.

[CR4] Caro AA, Cederbaum AI (2004). Antioxidant properties of S-adenosyl-l-methionine in Fe^2+^-initiated oxidations. Free Radical Biol Med.

[CR5] Chang Q, Griest TA, Harter TM, Petrash JM (2007). Functional studies of aldo-keto reductases in *Saccharomyces cerevisiae*. BBA-Mol Cell Res.

[CR6] Feng X, Zhao H (2013). Investigating host dependence of xylose utilization in recombinant *Saccharomyces cerevisiae* strains using RNA-seq analysis. Biotechnol Biofuels.

[CR7] Feng X, Zhao H (2013). Investigating xylose metabolism in recombinant *Saccharomyces cerevisiae* via ^13^C metabolic flux analysis. Microb Cell Factories.

[CR8] Gancedo JM (1998). Yeast carbon catabolite repression. Microbiol Mol Biol Rev.

[CR9] Hamacher T, Becker J, Gardonyi M, Hahn-Hägerdal B, Boles E (2002). Characterization of the xylose-transporting properties of yeast hexose transporters and their influence on xylose utilization. Microbiology.

[CR10] Hirasawa T, Yoshikawa K, Nakakura Y, Nagahisa K, Furusawa C, Katakura Y, Shimizu H, Shioya S (2007). Identification of target genes conferring ethanol stress tolerance to *Saccharomyces cerevisiae* based on DNA microarray data analysis. J Biotechnol.

[CR11] Jin YS, Laplaza JM, Jeffries TW (2004). *Saccharomyces cerevisiae* engineered for xylose metabolism exhibits a respiratory response. Appl Environ Microbiol.

[CR12] Kida K, Kume K, Morimura S, Sonoda Y (1992). Repeated-batch fermentation process using a thermotolerant flocculating yeast constructed by protoplast fusion. J Ferment Bioeng.

[CR13] Kuttykrishnan S, Sabina J, Langton LL, Johnston M, Brent MR (2010). A quantitative model of glucose signaling in yeast reveals an incoherent feed forward loop leading to a specific, transient pulse of transcription. Proc Natl Acad Sci USA.

[CR14] Li BZ, Cheng JS, Ding MZ, Yuan YJ (2010). Transcriptome analysis of differential responses of diploid and haploid yeast to ethanol stress. J Biotechnol.

[CR15] Li YC, Mitsumasu K, Gou ZX, Gou M, Tang YQ, Li GY, Wu XL, Akamatsu T, Taguchi H, Kida K (2015). Xylose fermentation efficiency and inhibitor tolerance of the recombinant industrial *Saccharomyces cerevisiae* strain NAPX37. Appl Microbiol Biotechnol.

[CR16] Matsushika A, Inoue H, Kodaki T, Sawayama S (2009). Ethanol production from xylose in engineered Saccharomyces cerevisiae strains, current state and perspectives. Appl Microbiol Biotechnol.

[CR17] Matsushika A, Inoue H, Murakami K, Takimura O, Sawayama S (2009). Bioethanol production performance of five recombinant strains of laboratory and industrial xylose-fermenting *Saccharomyces cerevisiae*. Bioresour Technol.

[CR18] Matsushika A, Goshima T, Fujii T, Inoue H, Sawayama S, Yano S (2012). Characterization of non-oxidative transaldolase and transketolase enzymes in the pentose phosphate pathway with regard to xylose utilization by recombinant *Saccharomyces cerevisiae*. Enzyme Microb Technol.

[CR19] Matsushika A, Goshima T, Hoshino T (2014). Transcription analysis of recombinant industrial and laboratory *Saccharomyces cerevisiae* strains reveals the molecular basis for fermentation of glucose and xylose. Microb Cell Factories.

[CR20] Mayer FV, Heath R, Underwood E, Sanders MJ, Carmena D, McCartney RR, Leiper FC, Xiao B, Jing C, Walker PA, Haire LF, Ogrodowicz R, Martin SR, Schmidt MC, Gamblin SJ, Carling D (2011). ADP regulates SNF1, the *Saccharomyces cerevisiae* homolog of AMP-activated protein kinase. Cell Metab.

[CR21] Palomino A, Herrero P, Moreno F (2005). Rgt1, a glucose sensing transcription factor, is required for transcriptional repression of the *HXK2* gene in *Saccharomyces cerevisiae*. Biochem J.

[CR22] Peng B, Shen Y, Li X, Chen X, Hou J, Bao X (2012). Improvement of xylose fermentation in respiratory-deficient xylose-fermenting *Saccharomyces cerevisiae*. Metab Eng.

[CR23] Regenberg B, Grotkjaer T, Winther O, Fausboll A, Akesson M, Bro C, Hansen LK, Brunak S, Nielsen J (2006). Growth-rate regulated genes have profound impact on interpretation of transcriptome profiling in *Saccharomyces cerevisiae*. Genome Biol.

[CR24] Runquist D, Hahn-Hägerdal B, Bettiga M (2009). Increased expression of the oxidative pentose phosphate pathway and gluconeogenesis in anaerobically growing xylose-utilizing *Saccharomyces cerevisiae*. Microb Cell Factories.

[CR25] Salusjärvi L, Pitkänen JP, Aristidou A, Ruohonen L, Penttilä M (2006). Transcription analysis of recombinant *Saccharomyces cerevisiae* reveals novel responses to xylose. Appl Biochem Biotechnol.

[CR26] Salusjärvi L, Kankainen M, Soliymani R, Pitkänen JP, Penttilä M, Ruohonen L (2008). Regulation of xylose metabolism in recombinant *Saccharomyces cerevisiae*. Microb Cell Factories.

[CR27] Tang Y, An M, Liu K, Nagai S, Shigematsu T, Morimura S, Kida K (2006). Ethanol production from acid hydrolysate of wood biomass using the flocculating yeast *Saccharomyces cerevisiae* strain KF-7. Process Biochem.

[CR28] Thomas D, SurdinKerjan Y (1997). Metabolism of sulfur amino acids in *Saccharomyces cerevisiae*. Microbiol Mol Biol Rev.

[CR29] Wasylenko TM, Stephanopoulos G (2015). Metabolomic and ^13^C-metabolic flux analysis of a xylose-consuming *Saccharomyces cerevisiae* strain expressing xylose isomerase. Biotechnol Bioeng.

[CR30] Zaman S, Lippman SI, Zhao X, Broach JR (2008). How *Saccharomyces* responds to nutrients. Annu Rev Genet.

