# Supplementary material for: Transcriptomes of a xylose-utilizing industrial flocculating Saccharomyces cerevisiae strain cultured in media containing different sugar sources
Source: AMB Express. 2016 Aug 2;6:51. doi: 10.1186/s13568-016-0223-y (PMC4970999; doi:10.1186/s13568-016-0223-y)
Supplement: Supplementary file 1 — 10.1186/s13568-016-0223-y Supplementary material. [file 13568_2016_223_MOESM1_ESM.pdf]

Supplementary Material

Submitted to: AMB Express

Title：

Transcriptomes of a xylose-utilizing industrial flocculating *Saccharomyces cerevisiae* strain cultured in media containing different sugar sources

Author: Wei-Yi Zeng[1], Yue-Qin Tang[1]*, Min Gou[1], Zi-Yuan Xia[1], Kenji Kida[1]

Affiliation: [1]College of Architecture and Environment, Sichuan University, No. 24, South Section 1, First Ring Road, Chengdu, Sichuan 610065, China

*Corresponding author

Tel. (fax): +86 28 85990936; Postal address: No. 24, South Section 1, First Ring Road, Chengdu, Sichuan 610065, China ; E-mail address: tangyq@scu.edu.cn

E-mail address:

Wei-Yi Zeng: 846372516@qq.com;

Min Gou: gouminscu@163.com;

Zi-Yuan Xia : xiaziyuan2014@hotmail.com;

Kenji Kida: kida@gpo.kumamoto-u.ac.jp.

Table S1. Primers used in RT-qPCR

Primer	Sequence 5′→3′
RT-XRs	TAT TGC TGT CAC CGC TTA C
RT-XRa	GGA GAC TTA CCG TGC TTA G
RT-XDHs	GCT ACT CCT AAC TCC AAG
RT-XDHa	AGA CAA TGG CTC AAC AAG
RT-SORs	TTCCTTGTGAAATTGCCAGA
RT-SORa	CTGTAGCTTGTTGTCGAATACATC
RT-ACTs	ATGCAAACCGCTGCTCAA
RT-ACTa	AGTTTGGTCAATACCGGCAGA

Table S2. Genes with significantly different transcription level in comparison 1

Gene Symbol	Ratio	Gene Function
POT1	2.27	3-ketoacyl-CoA thiolase with broad chain length specificity
ALD3	2.82	Cytoplasmic aldehyde dehydrogenase
CTT1	2.35	Cytosolic catalase T
YLR152C	2.17	Putative protein of unknown function
GCY1	3.26	Glycerol dehydrogenase
YNL195C	2.37	Protein of unknown function
DDR2	2.73	Multi-stress response protein
TDA6	2.14	Putative protein of unknown function
NQM1	3.12	Transaldolase of unknown function
YNR071C	2.60	Putative aldose 1-epimerase
SPG1	2.80	Protein required for high temperature survival during stationary phase
STL1	2.28	Glycerol proton symporter of the plasma membrane
DOG1	2.51	2-deoxyglucose-6-phosphate phosphatase
RTN2	2.99	Reticulon protein
ADH5	2.03	Alcohol dehydrogenase isoenzyme V
YJL133C-A	2.13	Putative protein of unknown function
YLR346C	0.41	Putative protein of unknown function found in mitochondria;
GIT1	0.39	Plasma membrane permease availability
PHO84	0.42	High-affinity inorganic phosphate (Pi) transporter
SNG1	0.45	Protein involved in resistance to nitrosoguanidine and 6-azauracil
ZPS1	0.44	Putative GPI-anchored protein
YLR460C	0.14	Member of the quinone oxidoreductase family
AAD6	0.38	Putative aryl-alcohol dehydrogenase
RRP12	0.47	Protein required for export of the ribosomal subunits
OYE3	0.30	Conserved NADPH oxidoreductase containing flavin mononucleotide
SPL2	0.15	Protein with similarity to cyclin-dependent kinase inhibitors
FCY22	0.39	Putative purine-cytosine permease
MHT1	0.39	S-methylmethionine-homocysteine methyltransferase
SNQ2	0.48	Plasma membrane ATP-binding cassette (ABC) transporter
PHO11 /// PHO12	0.23	One of three repressible acid phosphatases
DHR2	0.46	Predominantly nucleolar DEAH-box ATP-dependent RNA helicase
DUR1,2	0.49	Urea amidolyase
SDA1	0.48	Protein required for actin organization and passage through Start
AAH1	0.49	Adenine deaminase (adenine aminohydrolase)
PHO5	0.06	Repressible acid phosphatase
PHM6	0.18	Protein of unknown function

Table S3. Genes with significantly different transcription level in comparison 2

Gene Symbol	Ratio	Gene function
SOR1 /// SOR2	85.83	Sorbitol dehydrogenase
MAL12 /// MAL32	71.87	Maltase (alpha-D-glucosidase)
YNR071C	54.14	Putative aldose 1-epimerase
DSF1 /// YNR073C	45.06	Putative mannitol dehydrogenasedehydrogenase
HXT15 /// HXT16	44.56	Protein of unknown function with similarity to hexose transporters
FBP1	34.26	Fructose-1,6-bisphosphatase
FDH1	19.36	NAD(+)-dependent formate dehydrogenase
SFC1	19.23	Mitochondrial succinate-fumarate transporter
TAT1	17.89	Amino acid transporter for valine, leucine, isoleucine, and tyrosine
PCK1	17.19	Phosphoenolpyruvate carboxykinase
PRM5	13.36	Pheromone-regulated protein
HXT13	12.01	Hexose transporter
PIR3	11.56	O-glycosylated covalently-bound cell wall protein
FMP48	11.49	Putative protein of unknown function
NQM1	11.00	Transaldolase of unknown function
DDR2	10.35	Multi-stress response protein
FAT3	9.72	Protein required for fatty acid uptake
SPG1	9.12	Protein required for high temperature survival during stationary phase
YPS3	8.12	Aspartic protease
SHH3	8.00	Putative mitochondrial inner membrane protein of unknown function
AGX1	7.81	Alanine:glyoxylate aminotransferase (AGT)
HXT13	7.72	Hexose transporter
HXT13 /// HXT17	7.47	Hexose transporter
SUT1	7.36	Transcription factor of the Zn(II)2Cys6 family
HEF3	7.25	Translational elongation factor EF-3
CSR2	7.24	Nuclear ubiquitin protein ligase binding protein
FLO11	7.15	GPI-anchored cell surface glycoprotein (flocculin)
YMR206W	7.08	Putative protein of unknown function
IDP2	6.50	Cytosolic NADP-specific isocitrate dehydrogenase
HMX1	6.38	ER localized heme oxygenase
JEN1	6.37	Monocarboxylate/proton symporter of the plasma membrane
MAL31	6.34	Maltose permease
PRM10	6.25	Pheromone-regulated protein
CIT2	6.05	Citrate synthase
GDH3	5.97	NADP(+)-dependent glutamate dehydrogenase
CRC1	5.93	Mitochondrial inner membrane carnitine transporter
BAG7	5.83	Rho GTPase activating protein (RhoGAP)
ADR1	5.74	Carbon source-responsive zinc-finger transcription factor
NDE1	5.65	Mitochondrial external NADH dehydrogenase

MBR1	5.59	Protein involved in mitochondrial functions and stress response
MOH1	5.58	Protein of unknown function
SNO1	5.51	Protein of unconfirmed function
YIG1	5.45	Protein that interacts with glycerol 3-phosphatase
YHR033W	5.04	Putative protein of unknown function
CTA1	5.01	Catalase
YPK2	4.99	Protein kinase similar to serine/threonine protein kinase Ypk1p
YOL047C	4.98	Protein Involved in spore wall assembly
YNL195C	4.76	Protein of unknown function
HPA2	4.74	Tetrameric histone acetyltransferase
SPG4	4.71	Protein required for high temperature survival during stationary phase
FET3	4.69	Ferro-O2-oxidoreductase
PUT4	4.68	Proline permease
YPS6	4.66	Putative GPI-anchored aspartic protease
BAP2	4.63	High-affinity leucine permease
LYS20	4.62	Homocitrate synthase isozyme
ACH1	4.61	Protein with CoA transferase activity
MDH2	4.57	Cytoplasmic malate dehydrogenase
PRY2	4.54	Sterol binding protein involved in the export of acetylated sterols
SRL3	4.48	GTB motif (G1/S transcription factor binding) containing protein
SUC2	4.44	Invertase
RGI2	4.41	Protein of unknown function
TKL2	4.28	Transketolase
GUT2	4.26	Mitochondrial glycerol-3-phosphate dehydrogenase
NCA3	4.24	Protein involved in mitochondrion organization
SNZ1	4.22	Protein involved in vitamin B6 biosynthesis
ODC1	4.19	Mitochondrial inner membrane transporter
HXT5	4.13	Hexose transporter with moderate affinity for glucose
GIC2	4.03	Redundant rho-like GTPase Cdc42p effector
RIM4	3.95	Putative RNA-binding protein
YGR109W-A /// YGR109W-B /// YIL082W-A	3.86	Retrotransposon TYA Gag gene co-transcribed with TYB Pol
SPS1	3.82	Putative protein serine/threonine kinase
YJR149W	3.81	Putative protein of unknown function
AFR1	3.72	Protein required for pheromone-induced projection (shmoo) formation
GCY1	3.71	Glycerol dehydrogenase
SRT1	3.68	Cis-prenyltransferase
FIT3	3.67	Mannoprotein that is incorporated into the cell wall
CAT8	3.66	Zinc cluster transcriptional activator
SOK2	3.65	Nuclear protein that negatively regulates pseudohyphal

		differentiation
USV1	3.61	Putative transcription factor containing a C2H2 zinc finger
AAC1	3.60	Mitochondrial inner membrane ADP/ATP translocator
BDH1	3.57	NAD-dependent (R,R)-butanediol dehydrogenase
HSP30	3.53	Negative regulator of the H(+)-ATPase Pma1p
KNH1	3.51	Protein with similarity to Kre9p
SPO20	3.51	Meiosis-specific subunit of the t-SNARE complex
KDX1	3.51	Protein kinase
TOS8	3.51	Homeodomain-containing protein and putative transcription factor
MMP1	3.46	High-affinity S-methylmethionine permease
ATO2	3.40	"Putative transmembrane protein involved in export of ammonia
NDE2	3.39	Mitochondrial external NADH dehydrogenase
PDH1	3.37	Mitochondrial protein that participates in respiration
YKL107W	3.36	Putative short-chain dehydrogenase/reductase
PUT1	3.35	Proline oxidase
YBR056W-A	3.29	Protein of unknown function
URA8	3.26	Minor CTP synthase isozyme (see also URA7)
URA10	3.23	Minor orotate phosphoribosyltransferase (OPRTase) isozyme
PCL1	3.22	Cyclin
YIL024C	3.20	Putative protein of unknown function
CSM4	3.19	Protein required for accurate chromosome segregation during meiosis
ATO3	3.15	Plasma membrane protein, putative ammonium transporter
HSP32 /// HSP33 /// SNO4	3.13	Possible chaperone and cysteine protease
ICL2	3.13	2-methylisocitrate lyase of the mitochondrial matrix
PDR5	3.11	Plasma membrane ATP-binding cassette (ABC) transporter
LEE1	3.10	Zinc-finger protein of unknown function
SSA3	3.09	ATPase involved in protein folding and the response to stress
YCR007C	3.07	Putative integral membrane protein
SPO1	3.06	Meiosis-specific prospore protein
YJL107C	3.05	Putative protein of unknown function
RCK1	3.05	Protein kinase involved in the response to oxidative stress
HBT1	3.04	Shmoo tip protein
CAT2	3.03	Carnitine acetyl-CoA transferase
YOR338W	3.02	Putative protein of unknown function
RRT6	3.02	Putative protein of unknown function
YGR153W	3.00	Putative protein of unknown function
DIT1	2.98	Sporulation-specific enzyme required for spore wall maturation
YMR114C	2.98	Protein of unknown function
CWP1	2.97	Cell wall mannoprotein that localizes to birth scars of daughter cells
XBP1	2.95	Transcriptional repressor; binds to promoter sequences of the cyclin genes, CYS3, and SMF2

YNL277W-A	2.94	Putative protein of unknown function
RGM1	2.94	Putative zinc finger DNA binding transcription factor
CIN5	2.94	Basic leucine zipper transcription factor of the yAP-1 family
YLR031W	2.92	Putative protein of unknown function
YFL052W	2.91	Putative zinc cluster protein that contains a DNA binding domain
SLT2	2.87	Serine/threonine MAP kinase
FLC2	2.86	Putative FAD transporter
POG1	2.82	Nuclear chromatin-associated protein of unknown function
CRH1	2.82	Chitin transglycosylase
NCE103	2.80	Carbonic anhydrase
YIR035C	2.78	Putative cytoplasmic short-chain dehydrogenase/reductase
SCW10	2.77	Cell wall protein with similarity to glucanases
SUL1	2.75	High affinity sulfate permease of the SulP anion transporter family
YIL055C	2.75	Putative protein of unknown function
PLB3	2.74	Phospholipase B (lysophospholipase) involved in lipid metabolism
GSC2	2.74	Catalytic subunit of 1,3-beta-glucan synthase
YLR152C	2.73	Putative protein of unknown function
GND2	2.72	6-phosphogluconate dehydrogenase (decarboxylating)
YHB1	2.72	Nitric oxide oxidoreductase
CTR3	2.71	High-affinity copper transporter of the plasma membrane
PDC6	2.71	Minor isoform of pyruvate decarboxylase
IMA2 /// IMA3 /// IMA4	2.69	Alpha-glucosidase
ICL1	2.68	Isocitrate lyase
CLD1	2.66	Mitochondrial cardiolipin-specific phospholipase
UIP4	2.63	Protein that interacts with Ulp1p; a Ubl (ubiquitin-like protein)-specific protease for Smt3p protein conjugates
SGA1	2.62	Intracellular sporulation-specific glucoamylase
GAC1	2.62	Regulatory subunit for Glc7p type-1 protein phosphatase (PP1)
FMP16	2.61	Protein of unknown function
PTR2	2.60	Integral membrane peptide transporter
MID2	2.60	O-glycosylated plasma membrane protein
GIS3	2.59	Protein of unknown function
PCA1	2.59	Cadmium transporting P-type ATPase
YJL133C-A	2.58	Putative protein of unknown function
RIM8	2.56	Protein involved in proteolytic activation of Rim101p
BTN2	2.56	v-SNARE binding protein
SDH1	2.55	Flavoprotein subunit of succinate dehydrogenase
YLR307C-A	2.54	Putative protein of unknown function
YJL136W-A	2.53	Putative protein of unknown function
NGL3	2.52	3'-5' exonuclease specific for poly-A RNAs
YLR312C	2.52	Putative protein of unknown function
YMR315W-A	2.51	Putative protein of unknown function

MRK1	2.51	Glycogen synthase kinase 3 (GSK-3) homolog
YCR108C	2.51	Putative protein of unknown function
SSK22	2.51	MAP kinase kinase
MEP1	2.50	Ammonium permease
YFL054C	2.49	Putative channel-like protein
POX1	2.49	Fatty-acyl coenzyme A oxidase
YGR146C-A	2.48	Putative protein of unknown function
ADY2	2.47	Acetate transporter required for normal sporulation
PET10	2.47	Protein of unknown function that localizes to lipid particles
KNS1	2.47	Protein kinase involved in negative regulation of PolIII transcription
CIT3	2.47	Dual specificity mitochondrial citrate and methylcitrate synthase
FBP26	2.46	Fructose-2,6-bisphosphatase
RLM1	2.45	MADS-box transcription factor
GAT1	2.45	Transcriptional activator of nitrogen catabolite repression genes
RTA1	2.45	Protein involved in 7-aminocholesterol resistance
ARA2	2.44	NAD-dependent arabinose dehydrogenase
FMP46	2.44	Putative redox protein containing a thioredoxin fold
CRS5	2.43	Copper-binding metallothionein
YBL111C /// YEL077C /// YFL066C /// YHL050C /// YHR218W /// YIL177C /// YJL225C /// YLL066C /// YLL067C /// YML133C /// YPR204W /// YRF1-1 /// YRF1-2 /// YRF1-3 /// YRF1-4 /// YRF1-5 /// YRF1-6 /// YRF1-7 /// YRF1-8	2.42	Putative Y' element ATP-dependent helicase
YMR124W	2.42	Protein involved in septin-ER tethering
YOR394C-A	2.40	Protein of unknown function
HAL1	2.40	Cytoplasmic protein involved in halotolerance
RTN2	2.39	Reticulon protein
YDR034W-B	2.38	Predicted tail-anchored plasma membrane protein
SMC4	2.36	Subunit of the condensin complex
GAL3	2.35	Transcriptional regulator; involved in activation of the GAL genes in response to galactose
JID1	2.35	Probable Hsp40p co-chaperone
DIA1	2.35	Protein of unknown function
SPS19	2.34	Peroxisomal 2,4-dienoyl-CoA reductase

OYE3	2.34	Conserved NADPH oxidoreductase containing flavin mononucleotide (FMN)
NIS1	2.34	Protein localized in the bud neck at G2/M phase
ZWF1	2.33	Glucose-6-phosphate dehydrogenase (G6PD)
OM45	2.32	Mitochondrial outer membrane protein of unknown function
COX7	2.30	Subunit VII of cytochrome c oxidase (Complex IV)
PUF3	2.29	Protein of the mitochondrial outer surface
YPT53	2.28	Stress-induced Rab family GTPase
YBR071W	2.27	Protein of unknown function found in the cytoplasm and bud neck
NDI1	2.27	NADH:ubiquinone oxidoreductase
PTP2	2.27	Nuclear phosphotyrosine-specific phosphatase involved in osmosensing
YLR030W	2.27	Putative protein of unknown function
REG2	2.27	Regulatory subunit of the Glc7p type-1 protein phosphatase
SKM1	2.27	Member of the PAK family of serine/threonine protein kinases
YPL067C	2.27	Putative protein of unknown function
YJL077W-B	2.26	Putative protein of unknown function
CHS1	2.26	Chitin synthase I
ECM16	2.26	Essential DEAH-box ATP-dependent RNA helicase specific to U3 snoRNP
TRX3	2.25	Mitochondrial thioredoxin
NRG1	2.23	Transcriptional repressor; recruits the Cyc8p-Tup1p complex to promoters
REC102	2.23	Protein involved in early stages of meiotic recombination
ADY3	2.23	"Protein required for spore wall formation
CLN1	2.23	G1 cyclin involved in regulation of the cell cycle
YPL272C	2.22	Putative protein of unknown function
YML007C-A	2.21	Putative protein of unknown function
IDP3	2.21	Peroxisomal NADP-dependent isocitrate dehydrogenase
CTR1	2.21	High-affinity copper transporter of the plasma membrane
MIR1	2.21	Mitochondrial phosphate carrier
YLL066W-B	2.20	Putative protein of unknown function
VHS1	2.20	Cytoplasmic serine/threonine protein kinase
CBP4	2.20	Mitochondrial protein required for assembly of cytochrome bc1 complex
MPH1	2.18	3'-5' DNA helicase involved in error-free bypass of DNA lesions
CRG1	2.18	S-AdoMet-dependent methyltransferase involved in lipid homeostasis
AGP2	2.17	Plasma membrane regulator of polyamine and carnitine transport
CIR2	2.17	Putative ortholog of human ETF-dH
SPI1	2.17	GPI-anchored cell wall protein involved in weak acid resistance
MTL1	2.17	Putative plasma membrane sensor
MPC54	2.17	Component of the meiotic outer plaque

AVT3	2.17	Vacuolar transporter
PRB1	2.16	Vacuolar proteinase B with H3 N-terminal endopeptidase activity
RAD54	2.16	DNA-dependent ATPase that stimulates strand exchange
YHL008C	2.16	Putative protein of unknown function; may be involved in the uptake of chloride ions
SDH4	2.15	Membrane anchor subunit of succinate dehydrogenase
CMK2	2.14	Calmodulin-dependent protein kinase
HAP4	2.14	Transcription factor
RAD51	2.13	Strand exchange protein
SUE1	2.13	Protein required for degradation of unstable forms of cytochrome c
IZH3	2.13	Membrane protein involved in zinc ion homeostasis
YDR018C	2.13	robable membrane protein with three predicted transmembrane domains
PAU7	2.13	Member of the seripauperin multigene family
GPA1	2.12	Subunit of the G protein involved in pheromone response
QCR2	2.12	Subunit 2 of ubiquinol cytochrome-c reductase (Complex III)
MEF2	2.12	Mitochondrial elongation factor involved in translational elongation
YKL071W	2.12	Putative protein of unknown function
CYC1	2.11	Cytochrome c, isoform 1
ACS1	2.11	Acetyl-coA synthetase isoform
YMR001C-A	2.11	Putative protein of unknown function
YBL111C /// YFL065C /// YHL049C /// YHR218W /// YIL177C /// YJL225C /// YLL066C /// YLL067C /// YML133C /// YPR203W /// YRF1-1 /// YRF1-2 /// YRF1-3 /// YRF1-5 /// YRF1-6 /// YRF1-7 /// YRF1-8	2.11	Putative Y' element ATP-dependent helicase
GIP2	2.10	Putative regulatory subunit of protein phosphatase Glc7p
ATG8	2.10	Component of autophagosomes and Cvt vesicles
MLS1	2.09	Malate synthase
HAT1	2.09	Catalytic subunit of the Hat1p-Hat2p histone acetyltransferase complex
SAC7	2.09	GTPase activating protein (GAP) for Rho1p
YIR014W	2.09	Putative protein of unknown function
COX12	2.09	Subunit VIb of cytochrome c oxidase
YBR241C	2.08	Putative transporter, member of the sugar porter family
SSP2	2.07	Sporulation specific protein that localizes to the spore wal

OPY2	2.07	Integral membrane protein that acts as a membrane anchor for Ste50p
SIM1	2.07	Protein of the SUN family (Sim1p, Uth1p, Nca3p, Sun4p)
YER190C-B /// YFL068W	2.07	Putative protein of unknown function
GRE1	2.06	Hydrophilin essential in desiccation-rehydration process
APJ1	2.06	Chaperone with a role in SUMO-mediated protein degradation
NFS1	2.06	Cysteine desulfurase
ESF1	2.06	Nucleolar protein involved in pre-rRNA processing
YNG1	2.05	Subunit of the NuA3 histone acetyltransferase complex
YCL012C	2.05	Putative protein of unknown function
DIA3	2.05	Protein of unknown function
HIF1	2.05	Non-essential component of the HAT-B histone acetyltransferase complex
YME2	2.05	Integral inner mitochondrial membrane protein
LCL1	2.04	Putative protein of unknown function
OSW2	2.04	Protein of unknown function reputedly involved in spore wall assembly
YDL129W	2.04	Protein of unknown function
PSY3	2.04	Component of the Shu complex, which promotes error-free DNA repair
YIR018C-A	2.04	Putative protein of unknown function
HCA4	2.04	DEAD box RNA helicase
PRY1	2.03	Sterol binding protein involved in the export of acetylated sterols
YNL034W	2.03	Putative protein of unknown function
SFL1	2.02	Transcriptional repressor and activator
YFL067W	2.02	Protein of unknown function
DBP6	2.01	Essential protein involved in ribosome biogenesis
CYC3	2.01	Cytochrome c heme lyase (holocytochrome c synthase)
CYB2	2.01	Cytochrome b2 (L-lactate cytochrome-c oxidoreductase)
KGD1	2.00	Subunit of the mitochondrial alpha-ketoglutarate dehydrogenase complex
TRE1	2.00	Transferrin receptor-like protein
YKR045C	2.00	Putative protein of unknown function
GPI11	0.50	ER membrane protein involved in a late step of GPI anchor assembly
NSI1	0.50	RNA polymerase I termination factor
SEC63	0.50	Essential subunit of Sec63 complex
SSA2	0.50	ATP-binding protein
SCS3	0.49	Protein required for inositol prototrophy
TDA4	0.49	Putative protein of unknown function
RUD3	0.49	Golgi matrix protein
YCH1	0.49	Phosphatase with sequence similarity to Cdc25p

ORT1	0.49	Ornithine transporter of the mitochondrial inner membrane
TRS130	0.49	Component of transport protein particle (TRAPP) complex II
AFI1	0.49	Arf3p polarization-specific docking factor
YDL085C-A	0.49	Putative protein of unknown functio
YHC3	0.49	Protein required for the ATP-dependent transport of arginine
PFK27	0.49	6-phosphofructo-2-kinase
TMN2	0.49	Protein with a role in cellular adhesion and filamentous growth
PSF3	0.49	Subunit of the GINS complex (Sld5p, Psf1p, Psf2p, Psf3p)
YPL199C	0.49	Putative protein of unknown function
ARO7	0.49	Chorismate mutase
ATF2	0.49	Alcohol acetyltransferas
BNA4	0.49	Kynurenine 3-mono oxygenase
MOT1	0.49	Essential protein involved in regulation of transcription
PER1	0.48	Protein of the endoplasmic reticulum
EXG2	0.48	Exo-1,3-beta-glucanase
PMI40	0.48	Mannose-6-phosphate isomerase
YBR200W-A	0.48	Putative protein of unknown function
DAD3	0.48	Essential subunit of the Dam1 complex (aka DASH complex)
VBA4	0.48	Protein of unknown function
YOR390W /// YPL279C	0.48	Protein involved in fluoride export
APC4	0.48	Subunit of the Anaphase-Promoting Complex/Cyclosome (APC/C)
PFF1	0.48	Multi-spanning vacuolar membrane protease
RFA3	0.48	Subunit of heterotrimeric Replication Protein A (RPA)
YBR285W	0.48	Putative protein of unknown function
YPL041C	0.48	Protein of unknown function involved in maintenance of telomere length
AAT1	0.48	Mitochondrial aspartate aminotransferase
FAT1	0.48	Very long chain fatty acyl-CoA synthetase and fatty acid transporter
VTC4	0.48	Vacuolar membrane polyphosphate polymeras
YMR130W	0.48	Putative protein of unknown function
APA1	0.48	AP4A phosphorylase;
THI11 /// THI12 /// THI13 /// THI5	0.48	Protein involved in synthesis of the thiamine precursor HMP;
POL30	0.48	Proliferating cell nuclear antigen (PCNA)
THP2	0.48	Subunit of the THO and TREX complexes
GAS3	0.48	Putative 1,3-beta-glucanosyltransferase
MIG1	0.47	Transcription factor involved in glucose repression
PBP2	0.47	RNA binding protein
UTP22	0.47	Component of the small-subunit processome
VTC2	0.47	Subunit of vacuolar transporter chaperone (VTC) complex
YLL053C	0.47	Putative protein
ROY1	0.47	GTPase inhibitor with similarity to F-box proteins

HIS3	0.47	Imidazoleglycerol-phosphate dehydratase
PER33	0.47	Protein that localizes to the endoplasmic reticulum
YHL042W	0.47	Putative protein of unknown function
RTC3	0.47	Protein of unknown function involved in RNA metabolism
LEU2	0.47	Beta-isopropylmalate dehydrogenase (IMDH)
THI6	0.47	Thiamine-phosphate diphosphorylase and hydroxyethylthiazole kinase
HIS7	0.47	Imidazole glycerol phosphate synthase
THI7	0.47	Plasma membrane transporter responsible for the uptake of thiamine
SEC2	0.46	Guanyl-nucleotide exchange factor for the small G-protein Sec4p
YHR140W	0.46	Putative integral membrane protein of unknown function
MSH2	0.46	Protein that binds to DNA mismatches
PMT3	0.46	Protein O-mannosyltransferase
ADE17	0.46	Enzyme of 'de novo' purine biosynthesis
YDR179W-A	0.46	Putative protein of unknown function
YEL073C	0.46	Putative protein of unknown function
SOL4	0.46	6-phosphogluconolactonase
MED11	0.46	Subunit of the RNA polymerase II mediator complex
RPS9A	0.46	Protein component of the small (40S) ribosomal subunit
HTA2	0.46	Histone H2A
DAL7	0.46	Malate synthase
GSH2	0.45	Glutathione synthetase
YHL044W	0.45	Putative integral membrane protein
RPL18A /// RPL18B	0.45	Ribosomal 60S subunit protein L18B
YCL021W-A	0.45	Putative protein of unknown function
ERO1	0.45	Thiol oxidase required for oxidative protein folding in the ER
ASN1	0.45	Asparagine synthetase
MET17	0.45	O-acetyl homoserine-O-acetyl serine sulfhydrylase
HIS5	0.45	Histidinol-phosphate aminotransferase
SNZ2 /// SNZ3	0.45	Member of a stationary phase-induced gene family
LDB17	0.45	Protein involved in the regulation of endocytosis
WRS1	0.45	Cytoplasmic tryptophanyl-tRNA synthetase
CGI121	0.44	Component of the EKC/KEOPS complex
JEM1	0.44	DnaJ-like chaperone required for nuclear membrane fusion during mating
TYS1	0.44	Cytoplasmic tyrosyl-tRNA synthetase
SPC19	0.44	Essential subunit of the Dam1 complex (aka DASH complex)
EUG1	0.44	Protein disulfide isomerase of the endoplasmic reticulum lumen
PMU1	0.44	Putative phosphomutase
CTS2	0.44	Putative chitinase
SCJ1	0.44	One of several homologs of bacterial chaperone DnaJ
MAK3	0.44	Catalytic subunit of the NatC type N-terminal acetyltransferase
COS9	0.43	Protein of unknown function

UBC12	0.43	Enzyme that mediates the conjugation of Rub1p
ASP1	0.43	Cytosolic L-asparaginase
ERP2	0.43	Member of the p24 family involved in ER to Golgi transport
BIO4	0.43	Dethiobiotin synthetase
ZPS1	0.43	Putative GPI-anchored protein
SHE3	0.43	Protein adaptor between Myo4p and the She2p-mRNA complex
SWD3	0.43	Essential subunit of the COMPASS (Set1C) complex
YNR061C	0.43	Protein of unknown function
SRB2	0.43	Subunit of the RNA polymerase II mediator complex
GPM3	0.42	Homolog of Gpm1p phosphoglycerate mutase
MTD1	0.42	NAD-dependent 5,10-methylenetetrahydrafolate dehydrogenase
LHS1	0.42	Molecular chaperone of the endoplasmic reticulum lumen
YNL320W	0.42	Putative protein of unknown function
ALG12	0.42	Alpha-1,6-mannosyltransferase localized to the ER
FLD1	0.42	Seipin protein
SER33	0.42	3-phosphoglycerate dehydrogenase
KAP114	0.42	Karyopherin
DER1	0.41	ER membrane protein that promotes export of misfolded polypeptides
ERJ5	0.41	Type I membrane protein with a J domain
THI3	0.41	Regulatory protein that binds Pdc2p and Thi2p transcription factors
YPL279C	0.41	Protein involved in fluoride export
CTF18	0.41	Subunit of a complex with Ctf8p
LAA1	0.40	AP-1 accessory protein
HCH1	0.40	Heat shock protein regulator
IRC7	0.40	Beta-lyase involved in the production of thiols
EFR3	0.40	Protein required for Stt4-containing PI kinase complex localization
MET3	0.40	ATP sulfurylase
DUT1	0.40	deoxyuridine triphosphate diphosphatase (dUTPase
ADD66	0.39	Protein involved in 20S proteasome assembly
PHO84	0.39	High-affinity inorganic phosphate (Pi) transporter
RHO2	0.39	Non-essential small GTPase of the Rho/Rac family of Ras-like proteins
ECM29	0.39	Scaffold protein
GAL7	0.39	Galactose-1-phosphate uridyl transferase
MET5	0.39	Sulfite reductase beta subunit
NRK1	0.39	Nicotinamide riboside kinase
WWM1	0.38	WW domain containing protein of unknown function
YOL107W	0.38	Putative protein of unknown function
AQY2	0.38	Water channel that mediates water transport across cell membranes
HOM3	0.38	Aspartate kinase (L-aspartate 4-P-transferase)
LTE1	0.38	Protein similar to GDP/GTP exchange factors
ARO4	0.38	3-deoxy-D-arabino-heptulosonate-7-phosphate (DAHP) synthase

INM1	0.38	Inositol monophosphatase
THI2	0.38	Transcriptional activator of thiamine biosynthetic genes
YJL213W	0.38	Protein of unknown function that may interact with ribosomes
HTB2	0.37	Histone H2B
KAP122	0.37	Karyopherin beta
MET16	0.37	3'-phosphoadenylsulfate reductase
MPD1	0.37	Member of the protein disulfide isomerase (PDI) family
ARG7	0.37	Mitochondrial ornithine acetyltransferase
YMR209C	0.36	Putative S-adenosylmethionine-dependent methyltransferase
TRP4	0.36	Anthranilate phosphoribosyl transferase
HRI1	0.36	Protein of unknown function that interacts with Sec72p and Hrr25p
BNA3	0.35	Kynurenine aminotransferase
PHS1	0.35	Essential 3-hydroxyacyl-CoA dehydratase of the ER membrane
MRS2	0.35	Mitochondrial inner membrane Mg(2+) channel
AAD14	0.35	Putative aryl-alcohol dehydrogenase
MET6	0.34	Cobalamin-independent methionine synthase
ARO9	0.34	Aromatic aminotransferase II
YDR124W	0.34	Putative protein of unknown function
GDH1	0.34	NADP(+)-dependent glutamate dehydrogenase
KEG1	0.33	Integral membrane protein of the E
ANB1	0.33	Translation elongation factor eIF-5A
VTC3	0.33	Subunit of vacuolar transporter chaperone (VTC) complex
YKR075C	0.33	Protein of unknown function
HIS4	0.33	Multifunctional enzyme containing phosphoribosyl-ATP pyrophosphatase
MET22	0.33	Bisphosphate-3'-nucleotidase
SSU72	0.33	Phosphatase and transcription/RNA-processing factor
SSU1	0.33	Plasma membrane sulfite pump involved in sulfite metabolism
HXT4	0.32	High-affinity glucose transporter
ARG3	0.32	Ornithine carbamoyltransferase
PET18	0.32	Protein of unknown function
HEM13	0.32	Coproporphyrinogen III oxidase
ADE4	0.32	Phosphoribosylpyrophosphate amidotransferase (PRPPAT)
TVP15	0.31	Integral membrane protein
PHM6	0.31	Protein of unknown function
YPL264C	0.30	Putative membrane protein of unknown function
YNL024C	0.30	Putative methyltransferase
YOL155W-A	0.30	Putative protein of unknown function
MCD4	0.30	Protein involved in GPI anchor synthesis
PHO11 /// PHO12	0.30	One of three repressible acid phosphatases
BNA1	0.30	3-hydroxyanthranilic acid dioxygenase
HUG1	0.30	Protein involved in the Mec1p-mediated checkpoint pathway
EPT1	0.29	sn-1,2-diacylglycerol ethanolamine- and cholinephosphotranferase

YLR460C	0.29	Member of the quinone oxidoreductase family
POF1	0.28	Nicotinamide mononucleotide-specific adenylyltransferase (NMNAT)
SIL1	0.28	Nucleotide exchange factor for the ER lumenal Hsp70 chaperone Kar2p
SAM4 /// YMR321C	0.28	Putative protein of unknown function
SMF3	0.28	Putative divalent metal ion transporter involved in iron homeostasis
YNL234W	0.27	Protein of unknown function with similarity to globins
STD1	0.27	Protein involved in control of glucose-regulated gene expression
HIS1	0.27	ATP phosphoribosyltransferase
LPP1	0.27	Lipid phosphate phosphatase
THI80	0.26	Thiamine pyrophosphokinase
TAD2	0.26	Subunit of tRNA-specific adenosine-34 deaminase
CPT1	0.26	Cholinephosphotransferase
SNO2 /// SNO3	0.25	Protein of unknown function
ARI1	0.25	NADPH-dependent aldehyde reductase
TIR3	0.24	Cell wall mannoprotein
ARO10	0.24	Phenylpyruvate decarboxylase
AAC3	0.24	Mitochondrial inner membrane ADP/ATP translocator
GWT1	0.23	Protein involved in the inositol acylation of GlcN-PI
THI21	0.23	Hydroxymethylpyrimidine (HMP) and HMP-phosphate kinase
ARG4	0.22	Argininosuccinate lyase
MIG2	0.21	Zinc finger transcriptional repressor
PDC5	0.21	Minor isoform of pyruvate decarboxylase
THI74	0.20	Mitochondrial transporter repressible by thiamine
SAM4	0.19	S-adenosylmethionine-homocysteine methyltransferase
BUD16	0.19	Putative pyridoxal kinas
SPL2	0.18	Protein with similarity to cyclin-dependent kinase inhibitors
HXT2	0.16	High-affinity glucose transporter of the major facilitator superfamily
HXT3	0.14	Low affinity glucose transporter of the major facilitator superfamily
YDR541C	0.12	Putative dihydrokaempferol 4-reductase
GIT1	0.10	Plasma membrane permease
FCY22	0.10	Putative purine-cytosine permease
THI22	0.10	Protein with similarity to hydroxymethylpyrimidine phosphate kinases
DAN1	0.10	Cell wall mannoprotein
THI72	0.05	Transporter of thiamine or related compound

Table S4. Genes with significantly different transcription level in comparison 3

gene symbol	ratio	gene function
DSF1 /// YNR073C	48.86	Putative mannitol dehydrogenase
DAK2	25.21	Dihydroxyacetone kinase; involved in stress adaptation
STL1	22.56	Glycerol proton symporter of the plasma membrane
YNR071C	19.32	Putative aldose 1-epimerase
MMP1	17.87	High-affinity S-methylmethionine permease
SPG1	15.75	Protein required for high temperature survival during stationary phase
PUT4	14.51	Proline permease
PHO89	11.52	Plasma membrane Na+/Pi cotransporter
SPG4	11.40	Protein required for high temperature survival during stationary phase
PUT1	10.60	Proline oxidase
PRM10	10.21	Pheromone-regulated protein
OYE3	8.99	Conserved NADPH oxidoreductase containing flavin mononucleotide (FMN)
RGI2	8.90	Protein of unknown function
YKL107W	8.25	Putative short-chain dehydrogenase/reductase
PIR3	7.89	O-glycosylated covalently-bound cell wall protein
YCT1	7.87	High-affinity cysteine-specific transporter
FMP48	7.84	Putative protein of unknown function
HSP32 /// HSP33 /// SNO4	7.84	Possible chaperone and cysteine protease
PRM5	7.54	Pheromone-regulated protein
CTA1	7.32	Catalase A
HXT13 /// HXT17	7.19	Hexose transporter
TOP3	6.93	DNA Topoisomerase II
SPO16	6.92	Meiosis-specific protein involved in synaptonemal complex assembly
BAG7	6.89	Rho GTPase activating protein (RhoGAP)
YHB1	6.83	Nitric oxide oxidoreductase
NDE1	6.75	Mitochondrial external NADH dehydrogenase
HEF3	6.74	Translational elongation factor EF-3
SFC1	6.66	Mitochondrial succinate-fumarate transporter
SSA3	6.55	ATPase involved in protein folding and the response to stress
RAD59	6.51	Protein involved DNA double-strand break repair
SHH3	6.49	Putative mitochondrial inner membrane protein of unknown function
YIG1	6.48	Protein that interacts with glycerol 3-phosphatase
RAD54	6.13	DNA-dependent ATPase that stimulates strand exchange
YLR307C-A	6.07	Putative protein of unknown function
INO1	6.03	Inositol-3-phosphate synthase

HSP30	6.02	Negative regulator of the H(+)-ATPase Pma1p
AAD6	6.02	Putative aryl-alcohol dehydrogenase
MET28	5.99	bZIP transcriptional activator in the Cbf1p-Met4p-Met28p complex
MUP3	5.92	Low affinity methionine permease
HPA2	5.88	Tetrameric histone acetyltransferase
CAT8	5.81	Zinc cluster transcriptional activator
FAT3	5.71	Protein required for fatty acid uptake
HMX1	5.65	ER localized heme oxygenase
FMP16	5.63	Protein of unknown function
YNR064C	5.33	Epoxide hydrolase
YGR153W	5.23	Putative protein of unknown function
YOL047C	5.14	Protein Involved in spore wall assembly
SOR1 /// SOR2	5.11	Sorbitol dehydrogenase
UGX2	5.10	Protein of unknown function
GRE1	5.07	Hydrophilin essential in desiccation-rehydration process
YHR033W	5.06	Putative protein of unknown function
OSW1	5.02	Protein involved in sporulation
NQM1	4.94	Transaldolase of unknown function
MHT1	4.92	S-methylmethionine-homocysteine methyltransferase
YJR005C-A	4.89	Putative protein of unknown function
CIN5	4.77	Basic leucine zipper (bZIP) transcription factor of the yAP-1 family
ADR1	4.68	Carbon source-responsive zinc-finger transcription factor
IDP2	4.65	Cytosolic NADP-specific isocitrate dehydrogenase
CSR2	4.64	Nuclear ubiquitin protein ligase binding protein
YDR034W-B	4.61	Predicted tail-anchored plasma membrane protein
DDR2	4.56	Multi-stress response protein
STR3	4.56	Peroxisomal cystathionine beta-lyase
HXT5	4.50	Hexose transporter with moderate affinity for glucose
YIR035C	4.49	Putative cytoplasmic short-chain dehydrogenase
HAL1	4.35	Cytoplasmic protein involved in halotolerance
SPS19	4.33	Peroxisomal 2,4-dienoyl-CoA reductase
YML131W	4.25	Protein of unknown function
YGL138C	4.23	Putative protein of unknown function
YJL107C	4.19	Putative protein of unknown function
ATG11	4.16	Adapter protein for pexophagy and the Cvt targeting pathway
MET32	4.14	Zinc-finger DNA-binding transcription factor
AAD10	4.10	Putative aryl-alcohol dehydrogenas
ICL1	4.07	Isocitrate lyase
YNL195C	4.04	Protein of unknown function
SUL1	4.02	High affinity sulfate permease
GAT1	4.02	Transcriptional activator of nitrogen catabolite repression gene
SIP18	4.01	Phospholipid-binding hydrophilin
GRE2	3.96	3-methylbutanal reductase and NADPH-dependent methylglyoxal

		reductase
JEN1	3.96	Monocarboxylate/proton symporter of the plasma membrane
BAP2	3.96	High-affinity leucine permease
GAC1	3.92	Regulatory subunit for Glc7p type-1 protein phosphatase (PP1)
FBP1	3.91	Fructose-1,6-bisphosphatase
YKL050C	3.88	Protein of unknown function
NCE103	3.83	Carbonic anhydrase
YIL024C	3.80	Putative protein of unknown function
PRR2	3.79	Serine/threonine protein kinase
DDI1	3.75	DNA damage-inducible v-SNARE binding protein
RCK1	3.74	Protein kinase involved in the response to oxidative stressl
SUL2	3.74	High affinity sulfate permease
SKM1	3.73	Member of the PAK family of serine/threonine protein kinases
YFL054C	3.73	Putative channel-like protein
YPR078C	3.72	Putative protein of unknown function
MAL12 /// MAL32	3.67	Maltase (alpha-D-glucosidase
CRC1	3.66	Mitochondrial inner membrane carnitine transporter
YPS6	3.62	Putative GPI-anchored aspartic protease
ATG8	3.62	Component of autophagosomes and Cvt vesicles
CHS1	3.62	Chitin synthase I
FAP1	3.61	Protein that binds to Fpr1
MXR1	3.57	Methionine-S-sulfoxide reductase
SPO20	3.57	Meiosis-specific subunit of the t-SNARE complex
YLR312C	3.56	Putative protein of unknown function
ECM13	3.54	Non-essential protein of unknown function
DON1	3.53	Meiosis-specific component of the spindle pole body
MAL31	3.53	Maltose permease
AAD16 /// AAD4	3.52	Putative aryl-alcohol dehydrogenase
ADY2	3.51	Acetate transporter required for normal sporulation
APJ1	3.50	Chaperone with a role in SUMO-mediated protein degradation
RTP1	3.50	Protein required for the nuclear import and biogenesis of RNA pol II
PES4	3.48	Poly(A) binding protein
MAM1	3.47	Monopolin
RFA2	3.45	Subunit of heterotrimeric Replication Protein A
PDR5	3.44	Plasma membrane ATP-binding cassette transporter
LYS20	3.44	Homocitrate synthase isozyme
PCK1	3.43	Phosphoenolpyruvate carboxykinase
TOD6	3.43	PAC motif binding protein involved in rRNA and ribosome biogenesis
ALD6	3.43	Cytosolic aldehyde dehydrogenase
MET10	3.41	Subunit alpha of assimilatory sulfite reductase
GSM1	3.41	Putative zinc cluster protein of unknown function
CWP1	3.38	Cell wall mannoprotein that localizes to birth scars of daughter cell

AFR1	3.38	Protein required for pheromone-induced projection formation
YKL068W-A	3.38	Putative protein of unknown function
GDH3	3.36	NADP(+)-dependent glutamate dehydrogenase
BTN2	3.36	v-SNARE binding protein
FIT1	3.35	Mannoprotein that is incorporated into the cell wall
GSH1	3.33	Gamma glutamylcysteine synthetase
LSB6	3.33	Type II phosphatidylinositol 4-kinase
YJL144W	3.32	Cytoplasmic hydrophilin essential in desiccation-rehydration process
XBP1	3.32	Transcriptional repressor
MSH6	3.32	Protein required for mismatch repair in mitosis and meiosi
LEE1	3.31	Zinc-finger protein of unknown function
POT1	3.27	3-ketoacyl-CoA thiolase with broad chain length specificit
MND1	3.27	Protein required for recombination and meiotic nuclear division
MID2	3.27	O-glycosylated plasma membrane protei
GIC2	3.27	Redundant rho-like GTPase Cdc42p effector
GIS3	3.27	Protein of unknown function
TAT1	3.26	Amino acid transporter for valine, leucine, isoleucine, and tyrosine
PNS1	3.26	Protein of unknown function
SPG5	3.26	Protein required for proteasome assembly during quiescence
RDH54	3.24	DNA-dependent ATPas
GUT2	3.24	Mitochondrial glycerol-3-phosphate dehydrogenase
RAD51	3.23	Strand exchange protein
DIM1	3.23	Essential 18S rRNA dimethylase
ESC8	3.20	Protein involved in telomeric and mating-type locus silencing
YLR108C	3.17	Protein of unknown functio
FMP45	3.16	Integral membrane protein localized to mitochondria
MET2	3.15	L-homoserine-O-acetyltransferase
SUE1	3.15	Protein required for degradation of unstable forms of cytochrome c
SLT2	3.14	Serine/threonine MAP kinase
MET8	3.13	Bifunctional dehydrogenase and ferrochelatase
YMR034C	3.12	Putative transporter
BSC5	3.11	Protein of unknown function
MTL1	3.10	Putative plasma membrane sensor
CCC2	3.10	Cu(+2)-transporting P-type ATPase
ATG1	3.09	Protein serine/threonine kinase
RIM4	3.09	Putative RNA-binding protein
ACH1	3.07	Protein with CoA transferase activity
CHA1	3.06	Catabolic L-serine (L-threonine) deaminase
YDR262W	3.05	Putative protein of unknown function
DRE2	3.04	Component of the cytosolic Fe-S protein assembly machinery
YHR138C	3.01	Protein of unknown function
PTP2	3.01	Nuclear phosphotyrosine-specific phosphatase involved in

		osmosensing
SHC1	3.01	Sporulation-specific activator of Chs3p (chitin synthase III)
AVO2	3.01	Component of a complex containing the Tor2p kinase and other proteins
TRX3	3.00	Mitochondrial thioredoxin
ATO2	3.00	Putative transmembrane protein involved in export of ammonia
PDR10	2.99	ATP-binding cassette transporter
CIA2	2.98	Component of cytosolic iron-sulfur protein assembly machinery
CYC7	2.97	"Cytochrome c isoform 2
SRL3	2.95	GTB motif (G1/S transcription factor binding) containing protein
SSK22	2.95	MAP kinase kinase kinase of HOG1 mitogen-activated signaling pathway
YJR096W	2.94	Xylose and arabinose reductase
COX13	2.94	Subunit VIa of cytochrome c oxidase
YPS3	2.94	Aspartic protease
FLC2	2.93	Putative FAD transporter
HXT13	2.93	Hexose transporter
NOP7	2.93	Component of several different pre-ribosomal particle
MOH1	2.92	Protein of unknown function
CLD1	2.92	Mitochondrial cardiolipin-specific phospholipase
SMP1	2.91	MADS-box transcription factor involved in osmotic stress response
MET30	2.90	F-box protein containing five copies of the WD40 motif
ALD2	2.90	Cytoplasmic aldehyde dehydrogenase
OPY2	2.89	Integral membrane protein that acts as a membrane anchor for Ste50p
YMR114C	2.89	Protein of unknown function
BUD27	2.89	Unconventional prefoldin protein involved in translation initiation
SEN34	2.89	Subunit of the tRNA splicing endonuclease
YOR152C	2.88	Putative protein of unknown function
YDR042C	2.88	Putative protein of unknown function
GSC2	2.88	Catalytic subunit of 1,3-beta-glucan synthase
MET1	2.88	S-adenosyl-L-methionine uroporphyrinogen III transmethylase
MDH2	2.84	Cytoplasmic malate dehydrogenase
RSF1	2.81	Protein required for respiratory growth
YGR066C	2.80	Putative protein of unknown function
SUT1	2.80	Transcription factor of the Zn(II)2Cys6 family
ZDS2	2.79	Protein with a role in regulating Swe1p-dependent polarized growt
HMLALPHA2 /// HMRA2 /// MATALPHA2	2.78	Silenced copy of ALPHA2 at HML
UPS2	2.78	Mitochondrial intermembrane space protein
ATG3	2.78	E2-like enzyme
YME2	2.78	Integral inner mitochondrial membrane protein

CRS5	2.78	Copper-binding metallothionein
YFL052W	2.76	Putative zinc cluster protein that contains a DNA binding domain
MET4	2.76	Leucine-zipper transcriptional activator
CRG1	2.75	S-AdoMet-dependent methyltransferase involved in lipid homeostasis
RFA1	2.75	Subunit of heterotrimeric Replication Protein A
YPL119C-A	2.75	Putative protein of unknown function
PHM7	2.74	Protein of unknown functio
RIM8	2.74	Protein involved in proteolytic activation of Rim101p
FKS3	2.73	Protein involved in spore wall assembly
MHO1	2.73	Protein of unknown function
SSL2	2.73	Component of RNA polymerase transcription factor TFIIH holoenzyme
PET10	2.73	Protein of unknown function that localizes to lipid particles
ROG3	2.72	alpha-arrestin family membe
PHO8	2.71	Repressible vacuolar alkaline phosphatase
IFM1	2.70	Mitochondrial translation initiation factor 2
RAD27	2.70	5' to 3' exonuclease
PHO81	2.70	Cyclin-dependent kinase inhibitor
KNS1	2.69	Protein kinase involved in negative regulation of PolIII transcription
TDA7	2.68	Cell cycle-regulated gene of unknown function
TKL2	2.68	Transketolase
UBC5	2.68	Ubiquitin-conjugating enzyme
PIB1	2.68	RING-type ubiquitin ligase of the endosomal and vacuolar membranes
YDL114W	2.67	Putative short-chain dehydrogenase/reductase
FBP26	2.67	Fructose-2,6-bisphosphatase
MUP1	2.67	High affinity methionine permeas
YOR338W	2.67	Putative protein of unknown function
KEL2	2.66	Protein that negatively regulates mitotic exit
YMR206W	2.66	Putative protein of unknown function
YGR035C	2.66	Putative protein of unknown function
CRH1	2.66	Chitin transglycosylase
HAL9	2.65	Putative transcription factor containing a zinc finger
TMA10	2.64	"Protein of unknown function that associates with ribosomes
YGR067C	2.63	Putative protein of unknown function
KHA1	2.62	Putative K+/H+ antiporter
LRP1	2.62	Nuclear exosome-associated nucleic acid binding protein
MIC17	2.61	Mitochondrial intermembrane space protein
SLK19	2.61	Kinetochore-associated protein
YNL194C	2.60	Integral membrane protein
BCP1	2.60	Essential protein involved in nuclear export of Mss4p
PDH1	2.59	Mitochondrial protein that participates in respiration

RAD5	2.59	DNA helicase/Ubiquitin ligase
PEX18	2.59	Peroxin
ATG32	2.58	Mitochondrial outer membrane protein required to initiate mitophagy
COX5A	2.58	Subunit Va of cytochrome c oxidase
YPL260W	2.57	Putative substrate of cAMP-dependent protein kinase (PKA)
YBR056W-A	2.57	Protein of unknown function
YEL1	2.55	Guanine nucleotide exchange factor specific for Arf3p
PRB1	2.55	Vacuolar proteinase B (yscB) with H3 N-terminal endopeptidase activity
RRT12	2.55	Probable subtilisin-family protease
YNL165W	2.55	Putative protein of unknown function
SMC1	2.54	Subunit of the multiprotein cohesin complex
YOL036W	2.54	Protein of unknown function
SGA1	2.54	Intracellular sporulation-specific glucoamylase
FDH1	2.54	NAD(+)-dependent formate dehydrogenase
TIS11	2.54	mRNA-binding protein expressed during iron starvation
RAD16	2.51	Nucleotide excision repair (NER) protein
YOR214C	2.50	Putative spore wall protein
CSM4	2.50	Protein required for accurate chromosome segregation during meiosis
RLM1	2.49	MADS-box transcription factor
SRT1	2.49	Cis-prenyltransferase
LEU1	2.48	Isopropylmalate isomerase
MLS1	2.48	Malate synthase
RPN4	2.47	Transcription factor that stimulates expression of proteasome genes
RAD28	2.47	Protein involved in DNA repair
ERR1 /// ERR2 /// ERR3	2.46	Enolase, a phosphopyruvate hydratase
OAZ1	2.46	Regulator of ornithine decarboxylase Spe1p
URA10	2.46	Minor orotate phosphoribosyltransferase (OPRTase) isozyme
UBC8	2.46	Ubiquitin-conjugating enzyme that regulates gluconeogenesis
YKL133C	2.45	Putative protein of unknown function
RPH1	2.45	JmjC domain-containing histone demethylase
TAF1	2.45	TFIID subunit
DIT2	2.45	N-formyltyrosine oxidase
SOK2	2.44	Nuclear protein that negatively regulates pseudohyphal differentiation
ISF1	2.44	Serine-rich, hydrophilic protein
RAT1	2.44	Nuclear 5' to 3' single-stranded RNA exonuclease
NAR1	2.43	Subunit of the cytosolic iron-sulfur (FeS) protein assembly machinery
ASF2	2.43	Anti-silencing protein

NAB2	2.43	Nuclear polyadenylated RNA-binding protein
RRT6	2.43	Putative protein of unknown function
MTG2	2.43	Putative GTPase
HSP31	2.42	Methylglyoxalase that converts methylglyoxal to D-lactate
PEX11	2.42	Peroxisomal protein required for medium-chain fatty acid oxidation
AVT3	2.42	Vacuolar transporter
BSC4	2.42	Protein of unknown function
BXI1	2.42	Protein involved in apoptosis
ELP4	2.41	Subunit of hexameric RecA-like ATPase Elp456 Elongator subcomplex
NSE1	2.41	Component of the SMC5-SMC6 complex
MAG1	2.41	3-methyl-adenine DNA glycosylase
DFG5	2.40	Putative mannosidase
MIH1	2.40	Protein tyrosine phosphatase involved in cell cycle control
HBT1	2.40	Shmoo tip protein
GPN2	2.38	Putative GTPase with a role in biogenesis of RNA pol II and polIII
NMA111	2.38	Serine protease and general molecular chaperone
FAA2	2.38	Medium chain fatty acyl-CoA synthetase
CAT2	2.37	Carnitine acetyl-CoA transferase
BDH2	2.37	Putative medium-chain alcohol dehydrogenase
YOR032W-A	2.37	Protein of unknown function
TOK1	2.36	Outward-rectifier potassium channel of the plasma membrane;
MBR1	2.36	Protein involved in mitochondrial functions and stress respons
ECO1	2.36	Acetyltransferase
PSK2	2.35	PAS-domain containing serine/threonine protein kinas
ECM38	2.35	Gamma-glutamyltranspeptidase
PEX5	2.35	Peroxisomal membrane signal receptor for peroxisomal matrix proteins
LTV1	2.35	Component of the GSE complex
YNR068C	2.34	Putative protein of unknown function
SPC105	2.34	Subunit of a kinetochore-microtubule binding complex
YLR031W	2.34	Putative protein of unknown function
DBP1	2.33	Putative ATP-dependent RNA helicase of the DEAD-box protein family
AIM46	2.32	Putative protein of unknown function
COX7	2.32	Subunit VII of cytochrome c oxidase (Complex IV)
MET13	2.31	Major isozyme of methylenetetrahydrofolate reductase
YAP1	2.31	Basic leucine zipper (bZIP) transcription factor
YJL206C	2.31	Putative protein of unknown function
YGR079W	2.31	Putative protein of unknown function
DMA2	2.30	Ubiquitin-protein ligase (E3)
FLO11	2.30	GPI-anchored cell surface glycoprotein (flocculin)
NOP16	2.29	Constituent of 66S pre-ribosomal particles

UBR1	2.29	E3 ubiquitin ligase (N-recognin)
PET20	2.28	Mitochondrial protein
NOP6	2.28	rRNA-binding protein required for 40S ribosomal subunit biogenesis
NDE2	2.27	Mitochondrial external NADH dehydrogenase
JHD1	2.27	JmjC domain family histone demethylase specific for H3-K36
GIP2	2.27	Putative regulatory subunit of protein phosphatase Glc7
OM45	2.26	Mitochondrial outer membrane protein of unknown function
PCL7	2.26	Pho85p cyclin of the Pho80p subfamil
GLG2	2.26	Glycogenin glucosyltransferase
SPI1	2.26	GPI-anchored cell wall protein involved in weak acid resistance
TOS8	2.26	Homeodomain-containing protein and putative transcription factor
ACS1	2.25	Acetyl-coA synthetase isoform
RAD53	2.25	DNA damage response protein kinase;
RDS3	2.25	Component of the SF3b subcomplex of the U2 snRNP
STE11	2.25	Signal transducing MEK kinase
SWC5	2.25	Component of the SWR1 complex
COA2	2.25	Cytochrome oxidase assembly factor
YDR239C	2.24	Protein of unknown function
ISU1	2.24	Conserved protein of the mitochondrial matrix
NGL3	2.24	3'-5' exonuclease specific for poly-A RNAs
GAP1	2.24	General amino acid permease
GFD1	2.24	Coiled-coiled protein of unknown function
CUP2	2.24	Copper-binding transcription factor
STE7	2.24	Signal transducing MAP kinase kinase
NSE4	2.23	Component of the SMC5-SMC6 complex
ISR1	2.23	Predicted protein kinase
YBR085C-A	2.23	Protein of unknown function
MET14	2.23	Adenylylsulfate kinase
SMC6	2.23	Component of the SMC5-SMC6 complex
MPH1	2.22	3'-5' DNA helicase involved in error-free bypass of DNA lesions
YPR036W-A	2.22	Protein of unknown function
UFD4	2.22	Ubiquitin-protein ligase (E3)
YLR012C	2.22	Putative protein of unknown function
IME2	2.21	Serine/threonine protein kinase involved in activation of meiosis
GIN4	2.21	Protein kinase involved in bud growth and assembly of the septin ring
YLL056C	2.21	Putative protein of unknown function
PEX3	2.21	Peroxisomal membrane protein (PMP)
SFM1	2.21	SPOUT methyltransferase
ANS1	2.21	Putative GPI protein
HBN1	2.20	Protein of unknown function
YGR250C	2.20	Putative RNA binding protein

CDC7	2.20	DDK (Dbf4-dependent kinase) catalytic subunit
FMP10	2.20	Putative protein of unknown function
CSN9	2.20	Subunit of the Cop9 signalosome
YMR279C	2.19	Putative boron transporter involved in boron efflux and resistance
ICL2	2.19	2-methylisocitrate lyase of the mitochondrial matrix
ENT1	2.19	Epsin-like protein involved in endocytosis and actin patch assembly
GRX6	2.18	Cis-golgi localized monothiol glutaredoxin
SMY1	2.18	Kinesin-like myosin passenger-protein
MGR3	2.18	Subunit of the mitochondrial (mt) i-AAA protease supercomplex
YGL010W	2.18	Putative protein of unknown function
YPK2	2.18	Protein kinase similar to serine/threonine protein kinase Ypk1p
ACM1	2.18	Pseudosubstrate inhibitor of the APC/C
RRP15	2.18	Nucleolar protein
CRP1	2.17	Protein that binds to cruciform DNA structures
PSH1	2.17	E3 ubiquitin ligase targeting centromere-binding protein Cse4p
JID1	2.17	Probable Hsp40p co-chaperone
ENA1 /// ENA2 /// ENA5	2.17	Protein with similarity to P-type ATPase sodium pumps
SSP2	2.17	Sporulation specific protein that localizes to the spore wall
CYB2	2.17	Cytochrome b2 (L-lactate cytochrome-c oxidoreductase)
TYW1	2.16	Iron-sulfer protein required for synthesis of Wybutosine modified tRNA
RAD52	2.16	Protein that stimulates strand exchange
MET16	2.15	3'-phosphoadenylsulfate reductase
SAF1	2.15	F-Box protein involved in proteasome-dependent degradation of Aah1p
YDL183C	2.15	Protein that may form an active mitochondrial KHE system
ALD3	2.15	Cytoplasmic aldehyde dehydrogenase
MEF2	2.15	Mitochondrial elongation factor involved in translational elongation
AIM33	2.14	Putative protein of unknown function
YGL015C	2.14	Putative protein of unknown function
YMR085W	2.14	Putative protein of unknown function
FMP21	2.14	Protein required for assembly of succinate dehydrogenase
NAT4	2.14	N alpha-acetyl-transferase
SLM1	2.14	Phosphoinositide PI4,5P(2) binding protein
CWC24	2.14	General splicing factor
ALD4	2.13	Mitochondrial aldehyde dehydrogenase
ISA1	2.13	Protein required for maturation of mitochondrial [4Fe-4S] proteins
YOR389W /// YPL278C	2.13	Putative protein of unknown function
MRPS17	2.13	Mitochondrial ribosomal protein of the small subunit
STP2	2.13	Transcription factor
HIF1	2.13	Non-essential component of the HAT-B histone acetyltransferase

		complex
NBP35	2.13	Essential cytoplasmic iron-sulfur cluster binding protein
PEX4	2.13	Peroxisomal ubiquitin conjugating enzyme
FMO1	2.12	Flavin-containing monooxygenase
ATP22	2.12	Specific translational activator for the mitochondrial ATP6 mRNA
YBR071W	2.12	Protein of unknown function found in the cytoplasm and bud neck
CDC31	2.12	Calcium-binding component of the spindle pole body (SPB) half-bridge
YDR249C	2.12	Putative protein of unknown function
DAT1	2.11	DNA binding protein that recognizes oligo(dA).oligo(dT) tracts
NDI1	2.11	NADH:ubiquinone oxidoreductase
RMA1	2.11	Putative dihydrofolate synthetase;
YNR014W	2.11	Putative protein of unknown function
SLF1	2.11	RNA binding protein that associates with polysomes
HSP78	2.11	Oligomeric mitochondrial matrix chaperone
SNG1	2.11	Protein involved in resistance to nitrosoguanidine and 6-azauracil
YOR161C-C	2.11	Protein of unknown function
AVT4	2.11	Vacuolar transporter
IES2	2.10	Protein that associates with the INO80 chromatin remodeling complex
RPF1	2.10	Protein involved in assembly and export of the large ribosomal subunit
UBX6	2.10	UBX (ubiquitin regulatory X) domain-containing protein
GTS1	2.10	Protein involved in Arf3p regulation and in transcription regulation
PEX13	2.10	Peroxisomal importomer complex component
YER053C-A	2.10	Protein of unknown function
DDI2 /// DDI3	2.09	Protein of unknown function
UIP4	2.09	Protein that interacts with Ulp1p
FMP46	2.09	Putative redox protein containing a thioredoxin fold
YPC1	2.09	Alkaline ceramidase
IKS1	2.09	Protein kinase of unknown cellular role
RNA14	2.09	Component of the cleavage and polyadenylation factor I (CF I)
MIF2	2.09	Protein required for structural integrity of elongating spindles
XPT1	2.09	Xanthine-guanine phosphoribosyl transferase
LST7	2.09	Protein possibly involved in a post-Golgi secretory pathway
MET6	2.09	Cobalamin-independent methionine synthase
PIN3	2.09	Negative regulator of actin nucleation-promoting factor activit
ATG9	2.09	Transmembrane protein involved in forming Cvt and autophagic vesicles
DHR2	2.08	Predominantly nucleolar DEAH-box ATP-dependent RNA helicase
RRN3	2.08	Protein required for transcription of rDNA by RNA polymerase I
DIA3	2.08	Protein of unknown function
MCH1	2.08	Protein with similarity to mammalian monocarboxylate permeases

TEP1	2.08	PTEN homolog with no demonstrated inositol lipid phosphatase activity
PHM8	2.08	Lysophosphatidic acid (LPA) phosphatase, nucleotidase
CUL3	2.08	Ubiquitin-protein ligas
DUN1	2.08	Cell-cycle checkpoint serine-threonine kinase
SPC29	2.08	Inner plaque spindle pole body (SPB) component
AIM32	2.07	Putative protein of unknown function
RTC2	2.07	Putative vacuolar membrane transporter for cationic amino acids
RAD7	2.07	Nucleotide excision repair (NER) protein
YDR182W-A	2.07	Putative protein of unknown function
JJJ1	2.07	Co-chaperone that stimulates the ATPase activity of Ssa1p
SPS1	2.07	Putative protein serine/threonine kinase
YNG2	2.07	Subunit of NuA4, an essential histone acetyltransferase complex
SMC3	2.06	Subunit of the multiprotein cohesin complex
PAU5 /// PAU7	2.06	Member of the seripauperin multigene family
RBG2	2.06	Protein with a role in translation
VBA2	2.06	Permease of basic amino acids in the vacuolar membrane
PSP1	2.06	Asn and gln rich protein of unknown function
TIP41	2.06	Protein that interacts with Tap42p
PEP12	2.05	Target membrane receptor (t-SNARE
CYC3	2.05	Cytochrome c heme lyase (holocytochrome c synthase)
FUN19	2.05	Non-essential protein of unknown function
REI1	2.05	Cytoplasmic pre-60S factor
STP1	2.04	Transcription factor
YOR019W	2.04	Protein of unknown function
YMR124W	2.04	Protein involved in septin-ER tethering
REC102	2.04	Protein involved in early stages of meiotic recombination
NFI1	2.04	SUMO E3 ligase
CYT1	2.04	Cytochrome c1
ATG4	2.04	Conserved cysteine protease required for autophagy
PRM6	2.03	Potassium transporter that mediates K+ influx
BSD2	2.03	Heavy metal ion homeostasis protein
VHS1	2.03	Cytoplasmic serine/threonine protein kinase
SSH4	2.03	Specificity factor required for Rsp5p-dependent ubiquitination
YER085C	2.03	Putative protein of unknown function
URA8	2.03	Minor CTP synthase isozyme (see also URA7)
YDR246W-A	2.03	Putative protein of unknown function
JNM1	2.03	Component of the yeast dynactin complex
ATH1	2.03	Acid trehalase required for utilization of extracellular trehalose
BMS1	2.03	GTPase required for ribosomal subunit synthesis and rRNA processing
RAD14	2.02	Protein that recognizes and binds damaged DNA during NER
FMP40	2.02	Putative protein of unknown function

RRG9	2.02	Protein of unknown function
SIR1	2.02	Protein involved in silencing at mating-type loci HML and HMR
SRC1	2.02	Inner nuclear membrane protein
COX23	2.02	Protein that functions in mitochondrial copper homeostasi
UFE1	2.02	t-SNARE protein required for retrograde vesicular traffic
HSV2	2.02	Phosphatidylinositol 3,5-bisphosphate-binding protein
QCR9	2.02	Subunit 9 of ubiquinol cytochrome-c reductase (Complex III
DBP7	2.02	Putative ATP-dependent RNA helicase of the DEAD-box family
NRD1	2.01	RNA-binding subunit of Nrd1 complex
YDR222W	2.01	Protein of unknown function
PUT2	2.01	Delta-1-pyrroline-5-carboxylate dehydrogenase
YOR389W /// YPL278C	2.01	Putative protein of unknown function
YPL107W	2.01	Putative protein of unknown function
DAP1	2.01	Heme-binding protein
IME4	2.01	mRNA N6-adenosine methyltransferase required for entry into meiosis;
RSM18	2.01	Mitochondrial ribosomal protein of the small subunit
SPG3	2.01	Protein required for high temperature survival during stationary phase
CPA1	2.01	Small subunit of carbamoyl phosphate synthetase
PCS60	2.01	Oxalyl-CoA synthetase
SNQ2	2.01	Plasma membrane ATP-binding cassette (ABC) transporter
YNL295W	2.00	Putative protein of unknown function
SKP2	2.00	F-box protein of unknown function
YFL041W-A	2.00	Putative protein of unknown function
RMD6	2.00	Protein required for sporulation
SLX5	2.00	Subunit of the Slx5-Slx8 SUMO-targeted ubiquitin ligase complex
HIS2	0.500	Histidinolphosphatase
GCD10	0.500	Subunit of tRNA (1-methyladenosine) methyltransferase
pgk1	0.498	phosphoglycerate kinase Pgk1 (predicted)
OST6	0.498	Subunit of the oligosaccharyltransferase complex of the ER lumen
RPL23A /// RPL23B	0.498	Ribosomal 60S subunit protein L23A
VMA5	0.497	Subunit C of the V1 peripheral membrane domain of V-ATPase
CCR4	0.497	Component of the CCR4-NOT transcriptional complex
RPS9A	0.497	Protein component of the small (40S) ribosomal subunit
USA1	0.495	Scaffold subunit of the Hrd1p ubiquitin ligase
VMA2	0.495	Subunit B of V1 peripheral membrane domain of vacuolar H+-ATPase
VMA13	0.495	Subunit H of the V1 peripheral membrane domain of V-ATPase
EMC5	0.495	Member of conserved ER transmembrane complex
YNL024C	0.494	Putative methyltransferase
TED1	0.494	Conserved phosphoesterase domain-containing protein

RPL22B	0.493	Ribosomal 60S subunit protein L22B
FIR1	0.493	Protein involved in 3' mRNA processing
BUD31	0.493	Component of the SF3b subcomplex of the U2 snRN
OST3	0.493	Gamma subunit of the oligosaccharyltransferase complex of the ER lumen
HCS1	0.493	Hexameric DNA polymerase alpha-associated DNA helicase A
PTC5	0.492	Mitochondrial type 2C protein phosphatase
RET3	0.492	Zeta subunit of the coatomer complex
VTS1	0.492	Flap-structured DNA-binding and RNA-binding protein
LAS21	0.491	Integral plasma membrane protein
BTT1	0.491	Heterotrimeric nascent polypeptide-associated complex beta3 subunit
SAY1	0.490	Sterol deacetylase
SRL1	0.490	Mannoprotein that exhibits a tight association with the cell wall
RKM4	0.489	Ribosomal lysine methyltransferas
FRS1	0.489	Beta subunit of cytoplasmic phenylalanyl-tRNA synthetase
RPL16B	0.489	Ribosomal 60S subunit protein L16B
JEM1	0.489	DnaJ-like chaperone required for nuclear membrane fusion during mating
VAC8	0.489	Phosphorylated and palmitoylated vacuolar membrane protein
VMA8	0.488	Subunit D of the V1 peripheral membrane domain of V-ATPase
RPS1B	0.488	Ribosomal protein 10 (rp10) of the small (40S) subunit
INP54	0.488	Phosphatidylinositol 4,5-bisphosphate 5-phosphatase
NRM1	0.488	Transcriptional co-repressor of MBF-regulated gene expression
PCL9	0.488	Cyclin
FUI1	0.488	High affinity uridine permease,
LEU9	0.487	Alpha-isopropylmalate synthase II (2-isopropylmalate synthase)
GUS1	0.487	Glutamyl-tRNA synthetase
RPS18A /// RPS18B	0.487	Protein component of the small (40S) ribosomal subunit
LDB17	0.486	Protein involved in the regulation of endocytosis
YLR287C	0.486	Putative protein of unknown function
HOF1	0.486	SH3 domain-containing protein required for cytokinesis
GYP8	0.486	GTPase-activating protein for yeast Rab family member
ROT2	0.486	Glucosidase II catalytic subunit
FRS2	0.485	Alpha subunit of cytoplasmic phenylalanyl-tRNA synthetase
ADH5	0.484	Alcohol dehydrogenase isoenzyme V
LIA1	0.484	Deoxyhypusine hydroxylase
PHA2	0.484	Prephenate dehydratase
RRI1	0.484	Catalytic subunit of the COP9 signalosome (CSN) complex
YML079W	0.484	Non-essential protein of unknown functio
STD1	0.484	Protein involved in control of glucose-regulated gene expression
ASP1	0.484	Cytosolic L-asparaginase
PHO91	0.483	Low-affinity vacuolar phosphate transporter

RPL4B	0.483	Ribosomal 60S subunit protein L4B;
RPS10A	0.482	Protein component of the small (40S) ribosomal subunit
RPL3	0.482	Ribosomal 60S subunit protein L3
YBR220C	0.482	Putative protein of unknown function
RKI1	0.481	Ribose-5-phosphate ketol-isomerase
YNR061C	0.481	Protein of unknown function
MDJ2	0.480	Constituent of the mitochondrial import motor
TOS6	0.480	Glycosylphosphatidylinositol-dependent cell wall protein
YHR045W	0.480	Putative protein of unknown function
DOP1	0.480	Golgi-localized
DIB1	0.480	17-kDa component of the U4/U6aU5 tri-snRNP
YPR071W	0.480	Putative membrane protein
BUD8	0.479	Protein involved in bud-site selection
NUP82	0.479	Linker nucleoporin component of the nuclear pore complex
FUN26	0.479	High affinity, broad selectivity, nucleoside/nucleobase transporter
YIL161W	0.478	Putative protein of unknown function
SEC26	0.477	Essential beta-coat protein of the COPI coatomer
DRS2	0.477	Trans-golgi network aminophospholipid translocase (flippase)
YER152C	0.477	Protein with 2-aminoadipate transaminase activity
ERP1	0.477	Member of the p24 family involved in ER to Golgi transport
ERV25	0.476	Member of the p24 family involved in ER to Golgi transport
RPS22B	0.476	Protein component of the small (40S) ribosomal subunit
RPL24B	0.474	Ribosomal 60S subunit protein L24B
CTR2	0.474	Putative low-affinity copper transporter of the vacuolar membrane
VBA4	0.474	Protein of unknown function
ABP140	0.474	AdoMet-dependent tRNA methyltransferase and actin binding protein
SEC59	0.474	Dolichol kinase
AAD14	0.474	Putative aryl-alcohol dehydrogenase
SPS4	0.473	Protein whose expression is induced during sporulation
IMD3	0.473	Inosine monophosphate dehydrogenase
GAA1	0.473	Subunit of the GPI:protein transamidase complex
YOL019W	0.472	Protein of unknown function
TIF4632	0.472	"Translation initiation factor eIF4G
SEC6	0.471	Essential 88kDa subunit of the exocyst complex
DSE4	0.471	Daughter cell-specific secreted protein with similarity to glucanases
WTM1	0.471	Transcriptional modulator
YBR197C	0.470	Protein of unknown function
SHB17	0.470	Sedoheptulose bisphosphatase involved in riboneogenesis
LOS1	0.470	Nuclear pore protein
RPL15A	0.470	Ribosomal 60S subunit protein L15A; binds to 5.8 S rRNA
ICS3	0.470	Protein with a role in processing of secretory proteins
HAM1	0.469	Nucleoside triphosphate pyrophosphohydrolase

PMT6	0.469	Protein O-mannosyltransferase
BIL1	0.469	Protein that binds Bud6p and has a role in actin cable assembly
ADE13	0.469	Adenylosuccinate lyase
NSG1	0.468	Protein involved in regulation of sterol biosynthesis
ILM1	0.466	Protein of unknown function
YGR122W	0.466	Protein that may be involved in pH regulation
LEU2	0.466	Beta-isopropylmalate dehydrogenase (IMDH)
CWH41	0.465	Processing alpha glucosidase I
YMR130W	0.464	Putative protein of unknown function
HMF1	0.464	Member of the p14.5 protein family
RPS8A /// RPS8B	0.463	Protein component of the small (40S) ribosomal subunit
SUM1	0.463	Transcriptional repressor that regulates middle-sporulation genes
SEC7	0.462	Guanine nucleotide exchange factor (GEF) for ADP ribosylation factors
ALG8	0.462	Glucosyl transferase
TCB3	0.461	Cortical ER protein involved in ER-plasma membrane tethering
YCL021W-A	0.460	Putative protein of unknown function
SEC15	0.459	Essential 113 kDa subunit of the exocyst complex
HPA3	0.459	D-Amino acid N-acetyltransferase that detoxifies D-amino acids
FLO1	0.459	Lectin-like protein involved in flocculation
MIG2	0.458	Zinc finger transcriptional repressor
TIR4	0.457	Cell wall mannoprotein
GGA2	0.457	Protein that regulates Arf1p, Arf2p to facilitate Golgi trafficking
GPI15	0.457	Protein involved in the synthesis of GlcNAc-PI
LSM12	0.457	Protein of unknown function that may function in RNA processing
YDL085C-A	0.456	Putative protein of unknown function
YMR262W	0.455	Protein of unknown function
RUD3	0.455	Golgi matrix protein
DUS3	0.454	Dihydrouridine synthase
SIP3	0.454	Transcription cofactor
RPS22A	0.453	Protein component of the small (40S) ribosomal subunit
RPL12A /// RPL12B	0.453	Ribosomal 60S subunit protein L12A
CDC43	0.453	Beta subunit of geranylgeranyltransferase type I
YOX1	0.453	Homeobox transcriptional repressor
MYO1	0.452	Type II myosin heavy chain
MRI1	0.452	5'-methylthioribose-1-phosphate isomerase
RPL34A	0.452	Ribosomal 60S subunit protein L34A
GPM3	0.452	Homolog of Gpm1p phosphoglycerate mutase
HEM12	0.449	Uroporphyrinogen decarboxylase
YPQ1	0.449	Putative vacuolar membrane transporter for cationic amino acids
RPS8A /// RPS8B	0.449	Protein component of the small (40S) ribosomal subunit
ORC2	0.449	Subunit of the origin recognition complex (ORC
CAP2	0.448	Beta subunit of the capping protein heterodimer (Cap1p and Cap2p)

PDC5	0.448	Minor isoform of pyruvate decarboxylase
TAD3	0.448	Subunit of tRNA-specific adenosine-34 deaminase
CKA2	0.448	Alpha' catalytic subunit of casein kinase 2
VMA22	0.448	Protein that is required for vacuolar H+-ATPase (V-ATPase) function
RPC11	0.447	RNA polymerase III subunit C11
THI20	0.447	Trifunctional enzyme of thiamine biosynthesis
RBD2	0.446	Possible rhomboid protease
YDR379C-A	0.445	Mitochondrial protein involved in assembly of succinate dehydrogenase
YDR124W	0.444	Putative protein of unknown function
URA5	0.443	Major orotate phosphoribosyltransferase (OPRTase) isozyme
RPL6B	0.443	Ribosomal 60S subunit protein L6B
SPC2	0.443	Subunit of signal peptidase complex
MRS1	0.442	Splicing protein
SIM1	0.442	Protein of the SUN family
AMN1	0.441	Protein required for daughter cell separation
GIM3	0.441	Subunit of the heterohexameric cochaperone prefoldin complex
PRY3	0.441	Cell wall-associated protein involved in export of acetylated sterols
FMP41	0.441	Putative protein of unknown function
MYO2	0.441	Type V myosin motor involved in actin-based transport of cargos
SRP101	0.440	Signal recognition particle (SRP) receptor alpha subunit
SEC27	0.440	Essential beta'-coat protein of the COPI coatomer
TCD2	0.439	tRNA threonylcarbamoyladenosine dehydratase
GCS1	0.439	ADP-ribosylation factor GTPase activating protein
SCP160	0.438	Essential RNA-binding G protein effector of mating response pathway
PSP2	0.438	Asn rich cytoplasmic protein that contains RGG motifs
RPL8A /// RPL8B	0.436	Ribosomal 60S subunit protein L8B
CDC20	0.436	Activator of anaphase-promoting complex/cyclosome (APC/C)
PUS9	0.435	Mitochondrial tRNA:pseudouridine synthase
SDO1	0.435	Guanine nucleotide exchange factor (GEF) for Ria1p
COX19	0.435	Protein required for cytochrome c oxidase assembly
GDS1	0.433	Protein of unknown function
BER1	0.433	Protein involved in microtubule-related processes
YBR230W-A	0.433	Putative protein of unknown function
LTP1	0.432	Protein phosphotyrosine phosphatase of unknown cellular role
THR4	0.432	Threonine synthase
ERV29	0.432	Protein localized to COPII-coated vesicles
SFB2	0.431	Component of the Sec23p-Sfb2p heterodimer of the COPII vesicle coat;
YLR063W	0.431	Methyltransferase required for m3U2843 methylation of the 25S rRNA

IFA38	0.431	Microsomal beta-keto-reductase
YKR070W	0.430	Putative protein of unknown function
SNO2 /// SNO3	0.430	Protein of unknown function
OSH6	0.430	Member of an oxysterol-binding protein family
BNA4	0.429	Kynurenine 3-mono oxygenase
HOM3	0.429	Aspartate kinase (L-aspartate 4-P-transferase)
ENV10	0.428	Protein proposed to be involved in vacuolar functions
ARO10	0.427	Phenylpyruvate decarboxylase
GFD2	0.427	Protein of unknown function
RPS24A /// RPS24B	0.425	Protein component of the small (40S) ribosomal subunit
GNA1	0.425	Glucosamine-6-phosphate acetyltransferase
CCE1	0.425	Mitochondrial cruciform cutting endonuclease
EXO70	0.424	Subunit of the exocyst complex
YPR063C	0.424	ER-localized protein of unknown function
YHL044W	0.423	Putative integral membrane protein
YIL014C-A	0.423	Putative protein of unknown function
LOT6	0.422	FMN-dependent NAD(P)H:quinone reductase
SIL1	0.422	Nucleotide exchange factor for the ER lumenal Hsp70 chaperone Kar2p
GDT1	0.422	Protein of unknown function involved in calcium homeostasis
TYS1	0.422	Cytoplasmic tyrosyl-tRNA synthetase
IMD2	0.421	Inosine monophosphate dehydrogenase
CDC5	0.421	Polo-like kinase
GDH1	0.420	NADP(+)-dependent glutamate dehydrogenase;
CLN3	0.420	G1 cyclin involved in cell cycle progression
DTD1	0.419	D-Tyr-tRNA(Tyr) deacylase
TMA20	0.419	Protein of unknown function that associates with ribosomes
STT4	0.419	Phosphatidylinositol-4-kinase
NKP2	0.417	Central kinetochore protein and subunit of the Ctf19 complex
RFC1	0.417	Subunit of heteropentameric Replication factor C
MPD2	0.416	Member of the protein disulfide isomerase (PDI) family
RPL8A	0.415	Ribosomal 60S subunit protein L8A
PMT4	0.414	Protein O-mannosyltransferase
RPS7A	0.413	Protein component of the small (40S) ribosomal subunit
YLR118C	0.413	Acyl-protein thioesterase responsible for depalmitoylation of Gpa1p
ARC1	0.412	Protein that binds tRNA and methionyl- and glutamyl-tRNA synthetases
YLR179C	0.410	Protein of unknown function with similarity to Tfs1p
YHL042W	0.410	Putative protein of unknown function
KES1	0.410	One of seven members of the yeast oxysterol binding protein family
LOA1	0.409	Lysophosphatidic acid acyltransferase
NAT1	0.409	Subunit of protein N-terminal acetyltransferase NatA
YGL185C	0.408	Putative protein with sequence similar to hydroxyacid

		dehydrogenases
DIC1	0.408	Mitochondrial dicarboxylate carrier
BNA7	0.407	Formylkynurenine formamidase
LAP2	0.406	Leucyl aminopeptidase yscIV with epoxide hydrolase activity
PRS1	0.406	5-phospho-ribosyl-1(alpha)-pyrophosphate synthetase
SAC1	0.405	Phosphatidylinositol phosphate (PtdInsP) phosphatase
PYK2	0.404	Pyruvate kinase
RRN5	0.403	Protein involved in transcription of rDNA by RNA polymerase I
HTB2	0.403	Histone H2B
NUC1	0.403	Major mitochondrial nuclease
SNL1	0.403	Ribosome-associated protein
YCH1	0.400	Phosphatase with sequence similarity to Cdc25p
THI3	0.398	Regulatory protein that binds Pdc2p and Thi2p transcription factors
ARO8	0.398	Aromatic aminotransferase I
GIT1	0.396	Plasma membrane permease
SUN4	0.395	Cell wall protein related to glucanases
ALK1	0.394	Protein kinase
YEA4	0.393	Uridine diphosphate-N-acetylglucosamine (UDP-GlcNAc) transporter
RRI2	0.392	Subunit of the COP9 signalosome (CSN) complex
PSE1	0.391	Karyopherin/importin that interacts with the nuclear pore complex
PCL6	0.390	Pho85p cyclin of the Pho80p subfamily
RPS0A	0.390	Ribosomal 40S subunit protein S0A
EMC2	0.389	Member of conserved ER transmembrane complex
YHL017W	0.389	Putative protein of unknown function
UBC12	0.389	Enzyme that mediates the conjugation of Rub1p
DIA4	0.388	Probable mitochondrial seryl-tRNA synthetase
NMD5	0.388	Karyopherin
RPL17B	0.383	Ribosomal 60S subunit protein L17B
MCD4	0.379	Protein involved in GPI anchor synthesis
CAT5	0.377	Protein required for ubiquinone (Coenzyme Q) biosynthesis
ADH4	0.376	Alcohol dehydrogenase isoenzyme type IV
LAG1	0.376	Ceramide synthase component
MRF1	0.376	Mitochondrial translation release factor
SEC11	0.376	18kDa catalytic subunit of the Signal Peptidase Complex (SPC)
TYE7	0.375	Serine-rich protein that contains a bHLH DNA binding motif
BFR1	0.375	Component of mRNP complexes associated with polyribosomes
YNL046W	0.373	Putative protein of unknown function
YGL159W	0.373	Putative protein of unknown function
RPL18A /// RPL18B	0.372	Ribosomal 60S subunit protein L18B
YNL042W-B /// YOL013W-A /// YOR072W-B	0.372	Putative protein of unknown function

TVP15	0.371	Integral membrane protein
YPL191C	0.371	Putative protein of unknown function
YMR074C	0.369	Protein with homology to human PDCD5
TNA1	0.368	High affinity nicotinic acid plasma membrane permease
GCN1	0.367	Positive regulator of the Gcn2p kinase activity
TVP23	0.363	Integral membrane protein
DFR1	0.363	Dihydrofolate reductase involved in tetrahydrofolate biosynthesis
SUR4	0.363	Elongase
RPS28B	0.362	Protein component of the small (40S) ribosomal subunit
ERP2	0.362	Member of the p24 family involved in ER to Golgi transport
ASH1	0.361	Component of the Rpd3L histone deacetylase complex
GSH2	0.361	Glutathione synthetase
SAM4 /// YMR321C	0.360	Putative protein of unknown function
ARI1	0.360	NADPH-dependent aldehyde reductase
HXT4	0.359	High-affinity glucose transporter
HIS7	0.359	Imidazole glycerol phosphate synthase
ALG12	0.358	Alpha-1,6-mannosyltransferase localized to the ER
YBL005W-A /// YBL005W-B /// YDR170W-A /// YMR045C /// YMR046C /// YNL284C-A /// YNL284C-B	0.357	Retrotransposon TYA Gag and TYB Pol genes; transcribed/translated as one unit; polyprotein is processed to make a nucleocapsid-like protein (Gag), reverse transcriptase (RT), protease (PR), and integrase (IN); similar to retroviral genes
SEC63	0.357	Essential subunit of Sec63 complex
SNM1	0.357	Ribonuclease MRP complex subunit
TMN2	0.356	Protein with a role in cellular adhesion and filamentous growth
YBR238C	0.356	Mitochondrial membrane protein
GCR1	0.355	Transcriptional activator of genes involved in glycolysis
BUD16	0.355	Putative pyridoxal kinase
BUD4	0.353	Anillin-like protein involved in bud-site selection
SCS3	0.352	Protein required for inositol prototrophy
TRP4	0.352	Anthranilate phosphoribosyl transferase
YKL069W	0.350	Methionine-R-sulfoxide reductase
HEM15	0.347	Ferrochelatase
TAN1	0.347	Putative tRNA acetyltransferase
SEC14	0.346	Phosphatidylinositol/phosphatidylcholine transfer protein
BUD17	0.345	Putative pyridoxal kinase
YDL241W	0.345	Putative protein of unknown function
YNR021W	0.345	Putative protein of unknown function
RPL18A /// RPL18B	0.344	Ribosomal 60S subunit protein L18A
UBA3	0.344	Protein that activates Rub1p (NEDD8) before neddylation
PAC1	0.343	Involved in nuclear migration

THI21	0.343	Hydroxymethylpyrimidine (HMP) and HMP-phosphate kinase
SES1	0.341	Cytosolic seryl-tRNA synthetase
EUG1	0.339	Protein disulfide isomerase of the endoplasmic reticulum lumen
NAT5	0.338	Subunit of protein N-terminal acetyltransferase NatA
BNA1	0.337	3-hydroxyanthranilic acid dioxygenase
SLI1	0.336	N-acetyltransferase
AIM7	0.336	Protein that interacts with Arp2/3 complex
FPR2	0.335	Membrane-bound peptidyl-prolyl cis-trans isomerase (PPIase)
DSE2	0.334	Daughter cell-specific secreted protein with similarity to glucanases
RER2	0.333	Cis-prenyltransferase involved in dolichol synthesis
POF1	0.333	Nicotinamide mononucleotide-specific adenylyltransferase
MPD1	0.332	Member of the protein disulfide isomerase (PDI) family
SPC3	0.331	Subunit of signal peptidase complex
KIN3	0.331	Nonessential serine/threonine protein kinase
DYN1	0.330	Cytoplasmic heavy chain dynein
SRO9	0.329	Cytoplasmic RNA-binding protein
THI80	0.328	Thiamine pyrophosphokinase
TWF1	0.327	Twinfilin
LAA1	0.326	AP-1 accessory protein
YOL057W	0.325	Dipeptidyl-peptidase III
LYS12	0.324	Homo-isocitrate dehydrogenase
HMS2	0.323	Protein with similarity to heat shock transcription factors
YPT31	0.322	Rab family GTPase
RPL13A	0.320	Ribosomal 60S subunit protein L13A
HEM3	0.320	Porphobilinogen deaminase
GPM2	0.319	Homolog of Gpm1p phosphoglycerate mutase
AIM20	0.319	Putative protein of unknown function
CGI121	0.317	Component of the EKC/KEOPS complex
TCD1	0.315	tRNA threonylcarbamoyladenosine dehydratas
FLD1	0.313	Seipin protein
DER1	0.313	ER membrane protein that promotes export of misfolded polypeptides
HXK2	0.310	Hexokinase isoenzyme 2
SEC12	0.310	Guanine nucleotide exchange factor
PHS1	0.308	Essential 3-hydroxyacyl-CoA dehydratase of the ER membrane
HIS1	0.308	ATP phosphoribosyltransferase
PMU1	0.308	Putative phosphomutase
FCY22	0.307	Putative purine-cytosine permease
RRG8	0.302	Putative protein of unknown function
VPS55	0.301	Late endosomal protein involved in late endosome to vacuole transport
LYS9	0.299	Saccharopine dehydrogenase (NADP+, L-glutamate-forming);
IMD4	0.299	Inosine monophosphate dehydrogenase

OAR1	0.299	Mitochondrial 3-oxoacyl-[acyl-carrier-protein] reductase
DPM1	0.298	Dolichol phosphate mannose (Dol-P-Man) synthase of the ER membrane
CLB2	0.295	B-type cyclin involved in cell cycle progression
WRS1	0.293	Cytoplasmic tryptophanyl-tRNA synthetase
GYP6	0.291	GTPase-activating protein (GAP) for yeast Rab family member Ypt6p
TUB3	0.289	Alpha-tubulin
BUD9	0.288	Protein involved in bud-site selection
FEN1	0.287	Fatty acid elongase
PHO3	0.286	Constitutively expressed acid phosphatase
HEM13	0.286	Coproporphyrinogen III oxidase
TIR3	0.283	Cell wall mannoprotein
AIM44	0.283	Protein that regulates Cdc42p and Rho1p
SLD7	0.283	Protein with a role in chromosomal DNA replication
ANB1	0.282	Translation elongation factor eIF-5A
TRM10	0.281	tRNA methyltransferase
GWT1	0.280	Protein involved in the inositol acylation of GlcN-PI
YDR541C	0.279	Putative dihydrokaempferol 4-reductase
AAC3	0.279	Mitochondrial inner membrane ADP/ATP translocator
ARO4	0.279	3-deoxy-D-arabino-heptulosonate-7-phosphate (DAHP) synthase
SWI5	0.273	Transcription factor that recruits Mediator and Swi/Snf complexes
ALF1	0.271	Alpha-tubulin folding protein
SCW11	0.269	Cell wall protein with similarity to glucanases
ARD1	0.268	Subunit of protein N-terminal acetyltransferase Nat
SNZ2 /// SNZ3	0.266	Member of a stationary phase-induced gene family
AYR1	0.266	Bifunctional triacylglycerol lipase and 1-acyl DHAP reductase
EGT2	0.264	Glycosylphosphatidylinositol (GPI)-anchored cell wall endoglucanase
HRI1	0.264	Protein of unknown function that interacts with Sec72p and Hrr25p
RFU1	0.263	Protein that inhibits Doa4p deubiquitinating activity;
THI22	0.258	Protein with similarity to hydroxymethylpyrimidine phosphate kinases
HXT2	0.251	High-affinity glucose transporter of the major facilitator superfamily
SOL3	0.249	6-phosphogluconolactonase
IRC8	0.247	Bud tip localized protein of unknown function
CLB1	0.245	B-type cyclin involved in cell cycle progression
RAX1	0.239	Protein involved in bud site selection during bipolar budding
INM1	0.238	Inositol monophosphatase
SAM4	0.235	S-adenosylmethionine-homocysteine methyltransferase
TDA10	0.230	ATP-binding protein of unknown function
VPS75	0.229	NAP family histone chaperone
YCR015C	0.220	Putative protein of unknown function; YCR015C is not an essential

		gene
HXT3	0.212	Low affinity glucose transporter of the major facilitator superfamily
SFG1	0.204	Nuclear protein putative transcription factor
SRB2	0.197	Subunit of the RNA polymerase II mediator complex
THI72	0.132	Transporter of thiamine or related compound

Table S5. Genes with significantly different transcription level in comparison 4

Gene Symbol	Ratio	Gene Title
STL1	70.90	Glycerol proton symporter of the plasma membrane
STR3	9.76	Peroxisomal cystathionine beta-lyase
DAK2	9.57	Dihydroxyacetone kinase
PNS1	8.42	Protein of unknown function
PHO89	7.96	Plasma membrane Na+/Pi cotransporter
YCT1	7.31	High-affinity cysteine-specific transporter
SPO16	6.40	Meiosis-specific protein involved in synaptonemal complex assembly
DAN1	6.38	Cell wall mannoprotein
OSW1	6.08	Protein involved in sporulation
MET6	5.68	Cobalamin-independent methionine synthase
MUP3	5.47	Low affinity methionine permease
FIT1	5.27	Mannoprotein that is incorporated into the cell wall
RAD59	5.24	Protein involved DNA double-strand break repair
INO1	5.04	Inositol-3-phosphate synthase
YGL138C	4.97	Putative protein of unknown function
MET28	4.89	bZIP transcriptional activator in the Cbf1p-Met4p-Met28p complex
SPG1	4.83	Protein required for high temperature survival during stationary phase
ALD3	4.81	Cytoplasmic aldehyde dehydrogenase
UGX2	4.62	Protein of unknown function
MET16	4.55	3'-phosphoadenylsulfate reductase
SIP18	4.53	Phospholipid-binding hydrophilin
ARG4	4.49	Argininosuccinate lyase
GRE2	4.42	3-methylbutanal reductase and NADPH-dependent methylglyoxal reductase
HST4	4.38	Member of the Sir2 family of NAD(+)-dependent protein deacetylases
AVO2	4.37	Component of a complex containing the Tor2p kinase and other proteins
MAM1	4.00	Monopolin
MXR1	3.97	Methionine-S-sulfoxide reductase
SPG4	3.96	Protein required for high temperature survival during stationary phase
MET10	3.87	Subunit alpha of assimilatory sulfite reductase
YJR005C-A	3.83	Putative protein of unknown function
MET2	3.82	L-homoserine-O-acetyltransferase
POT1	3.77	3-ketoacyl-CoA thiolase with broad chain length specificity
DAL3	3.66	Ureidoglycolate lyase
RTC3	3.66	Protein of unknown function involved in RNA metabolism
ATG1	3.64	Protein serine/threonine kinase

YGR066C	3.62	Putative protein of unknown function
YOR152C	3.60	Putative protein of unknown function
YOR214C	3.60	Putative spore wall protein
IFM1	3.59	Mitochondrial translation initiation factor 2
PHM7	3.59	Protein of unknown function
YPR078C	3.46	Putative protein of unknown function
BNA3	3.46	Kynurenine aminotransferase
FMP16	3.44	Protein of unknown function
YDR182W-A	3.42	Putative protein of unknown function
YJR096W	3.41	Xylose and arabinose reductase
TOP3	3.39	DNA Topoisomerase III
SOL4	3.38	6-phosphogluconolactonase
PRR2	3.38	Serine/threonine protein kinase
ARG3	3.36	Ornithine carbamoyltransferase
HAL9	3.33	Putative transcription factor containing a zinc finger
HSP32 /// HSP33 /// SNO4	3.32	Possible chaperone and cysteine protease
YPL264C	3.32	Putative membrane protein of unknown function
SUL2	3.31	High affinity sulfate permease
MET13	3.30	Major isozyme of methylenetetrahydrofolate reductase
PFK27	3.30	6-phosphofructo-2-kinase
THI74	3.29	Mitochondrial transporter repressible by thiamine
YDL114W	3.28	Putative short-chain dehydrogenase/reductase
MND1	3.25	Protein required for recombination and meiotic nuclear division
ROG3	3.22	alpha-arrestin family member
RFA2	3.21	Subunit of heterotrimeric Replication Protein
YHR140W	3.21	Putative integral membrane protein of unknown function
YDR179W-A	3.21	Putative protein of unknown function
RRD2	3.21	Peptidyl-prolyl cis/trans-isomerase
PHM8	3.20	Lysophosphatidic acid (LPA) phosphatase, nucleotidase
SIR1	3.17	Protein involved in silencing at mating-type loci HML and HMR
RFA1	3.10	Subunit of heterotrimeric Replication Protein A
HSP30	3.09	Negative regulator of the H(+)-ATPase Pma1p
STE7	3.04	Signal transducing MAP kinase kinase
PEX17	3.04	Membrane peroxin of the peroxisomal importomer complex
AAD6	3.03	Putative aryl-alcohol dehydrogenase
FHN1	3.01	Protein of unknown function
CCL1	2.99	Cyclin associated with protein kinase Kin28p
MUP1	2.99	High affinity methionine permease
YDR034W-B	2.98	Predicted tail-anchored plasma membrane protein
PRM10	2.95	Pheromone-regulated protein
YGR053C	2.95	Putative protein of unknown function
YBR285W	2.94	Putative protein of unknown function

FMP23	2.94	Putative protein of unknown function
PUT1	2.93	Proline oxidase
LEU1	2.93	Isopropylmalate isomerase
YNR068C	2.92	Putative protein of unknown function
YPL279C	2.91	Protein involved in fluoride export
RNA14	2.90	Component of the cleavage and polyadenylation factor I
ECO1	2.90	Acetyltransferase
SMC1	2.89	Subunit of the multiprotein cohesin complex
OAZ1	2.89	Regulator of ornithine decarboxylase Spe1p
THP2	2.88	Subunit of the THO and TREX complexes
YJL144W	2.87	Cytoplasmic hydrophilin essential in desiccation-rehydration process
SNX41	2.87	Sorting nexin
IKS1	2.87	Protein kinase of unknown cellular rol
YNL234W	2.87	Protein of unknown function with similarity to globins
SAM2	2.86	S-adenosylmethionine synthetase
MET3	2.86	ATP sulfurylase
YKL107W	2.85	Putative short-chain dehydrogenase/reductase
DON1	2.84	Meiosis-specific component of the spindle pole body
SLK19	2.84	Kinetochore-associated protein
ECM29	2.83	Scaffold protein
SSL2	2.83	Component of RNA polymerase transcription factor TFIIH holoenzyme
KAR3	2.83	Minus-end-directed microtubule motor
GRE1	2.83	Hydrophilin essential in desiccation-rehydration process
PEX3	2.83	Peroxisomal membrane protein
DDI1	2.82	DNA damage-inducible v-SNARE binding protein
YLR312C	2.82	Putative protein of unknown function
YDL206W	2.81	Putative protein of unknown function
ECM13	2.79	Non-essential protein of unknown function
CIA2	2.79	Component of cytosolic iron-sulfur protein assembly (CIA) machinery
SPC29	2.78	Inner plaque spindle pole body (SPB) component
PES4	2.78	Poly(A) binding protein
POL30	2.78	Proliferating cell nuclear antigen
YSC83	2.77	Non-essential mitochondrial protein of unknown function
YPL247C	2.77	Putative protein of unknown function
RQC1	2.75	Component of the ribosome quality control complex
SLZ1	2.74	Sporulation-specific protein with a leucine zipper motif
RTC2	2.73	Putative vacuolar membrane transporter for cationic amino acids
HBN1	2.73	Protein of unknown function
MIF2	2.72	Protein required for structural integrity of elongating spindles
PUT4	2.72	Proline permease

PEX5	2.71	Peroxisomal membrane signal receptor for peroxisomal matrix proteins
AMA1	2.71	Activator of meiotic anaphase promoting complex
ATG11	2.69	Adapter protein for pexophagy and the Cvt targeting pathway
DSS1	2.68	3'-5' exoribonuclease
RSF1	2.68	Protein required for respiratory growth
SPS100	2.67	Protein required for spore wall maturation
MMP1	2.67	High-affinity S-methylmethionine permease
SMK1	2.67	Middle sporulation-specific mitogen-activated protein kinase
MET14	2.66	Adenylylsulfate kinase
RAV2	2.65	Subunit of RAVE complex
YKL133C	2.65	Putative protein of unknown function
CTT1	2.64	Cytosolic catalase T
PDS1	2.64	Securin
SKP2	2.64	F-box protein of unknown function
INP2	2.64	Peroxisome-specific receptor important for peroxisome inheritance
RTP1	2.64	Protein required for the nuclear import and biogenesis of RNA pol II
RRN3	2.64	Protein required for transcription of rDNA by RNA polymerase I
PHO8	2.63	Repressible vacuolar alkaline phosphatase
FUS1	2.62	Membrane protein localized to the shmoo tip
YDR042C	2.62	Putative protein of unknown function; expression is increased in ssu72-ts69 mutant
KEL2	2.62	Protein that negatively regulates mitotic exit
HUG1	2.62	Protein involved in the Mec1p-mediated checkpoint pathway
RAD27	2.62	5' to 3' exonuclease
YOL155W-A	2.60	Putative protein of unknown function
YMR034C	2.60	Putative transporter
YMR279C	2.60	Putative boron transporter involved in boron efflux and resistance
DXO1	2.60	mRNA 5'-end-capping quality-control protein
DGR1	2.59	Protein of unknown function
ALD6	2.58	Cytosolic aldehyde dehydrogenase
RAD24	2.58	Checkpoint protein
YDR249C	2.57	Putative protein of unknown function
MET1	2.57	S-adenosyl-L-methionine uroporphyrinogen III transmethylase
ORT1	2.57	Ornithine transporter of the mitochondrial inner membrane
MET32	2.56	Zinc-finger DNA-binding transcription factor
SAF1	2.56	F-Box protein involved in proteasome-dependent degradation of Aah1p
VHT1	2.55	High-affinity plasma membrane H+-biotin (vitamin H) symporter
YNL165W	2.55	Putative protein of unknown function
THI22	2.54	Protein with similarity to hydroxymethylpyrimidine phosphate kinases
HNM1	2.54	Plasma membrane transporter for choline, ethanolamine, and

		carnitine
CTS2	2.53	Putative chitinase
YLR108C	2.53	Protein of unknown function
EMP47	2.53	Integral membrane component of ER-derived COPII-coated vesicles
PRM2	2.52	Pheromone-regulated protein
AFI1	2.51	Arf3p polarization-specific docking factor
YLR297W	2.51	Protein of unknown function
MET17	2.51	O-acetyl homoserine-O-acetyl serine sulfhydrylase
RGI2	2.51	Protein of unknown function
PDR11	2.50	ATP-binding cassette (ABC) transporter
NPL4	2.49	Substrate-recruiting cofactor of the Cdc48p-Npl4p-Ufd1p segregase
NPR1	2.49	Protein kinase
RAD6	2.49	"Ubiquitin-conjugating enzyme (E2)
TEP1	2.49	PTEN homolog with no demonstrated inositol lipid phosphatase activity
KEG1	2.46	Integral membrane protein of the ER
YKL091C	2.46	Putative phosphatidylinositol/phosphatidylcholine transfer protein
ARG1	2.46	Arginosuccinate synthetase
OPI10	2.46	Protein with a possible role in phospholipid biosynthesis
YER085C	2.44	Putative protein of unknown function
CDC27	2.44	Subunit of the Anaphase-Promoting Complex/Cyclosome
AAD10	2.44	Putative aryl-alcohol dehydrogenase
WAR1	2.43	Homodimeric Zn2Cys6 zinc finger transcription factor
SAM37	2.42	Component of the Sorting and Assembly Machinery (SAM) complex
VBA2	2.42	Permease of basic amino acids in the vacuolar membrane
DRE2	2.42	Component of the cytosolic Fe-S protein assembly (CIA) machinery
SPC105	2.41	Subunit of a kinetochore-microtubule binding complex
MET8	2.40	Bifunctional dehydrogenase and ferrochelatase
CWC24	2.40	General splicing factor
ISU1	2.40	Conserved protein of the mitochondrial matrix
RAD53	2.40	DNA damage response protein kinase
SPG5	2.39	Protein required for proteasome assembly during quiescence
MCM16	2.39	Component of the Ctf19 complex and the COMA subcomplex
RDH54	2.39	DNA-dependent ATPase
PEX18	2.39	Peroxin
DDI2 /// DDI3	2.39	Protein of unknown function
REC104	2.38	Protein involved in early stages of meiotic recombination
SSA4	2.38	Heat shock protein that is highly induced upon stress
RAD52	2.38	Protein that stimulates strand exchange
SCJ1	2.38	One of several homologs of bacterial chaperone DnaJ
BRL1	2.38	Essential nuclear envelope integral membrane protein
YFL041W-A	2.38	Putative protein of unknown function

ARG5,6	2.38	Acetylglutamate kinase and N-acetyl-gamma-glutamyl-phosphate reductase
BRR6	2.37	Essential nuclear envelope integral membrane protein
NEJ1	2.37	Protein involved in regulation of nonhomologous end joining
YDL211C	2.37	Protein of unknown function; green fluorescent protein (GFP)-fusion protein localizes to the vacuole
SPT21	2.37	Protein with a role in transcriptional silencing
YIR035C	2.37	Putative cytoplasmic short-chain dehydrogenase/reductase
SPR28	2.36	Sporulation-specific homolog of the CDC3/10/11/12 family of genes
TSA2	2.36	Stress inducible cytoplasmic thioredoxin peroxidase
SEN34	2.35	Subunit of the tRNA splicing endonuclease
GPN2	2.35	Putative GTPase with a role in biogenesis of RNA pol II and polIII
CCC2	2.35	Cu(+2)-transporting P-type ATPase
RPN1	2.35	Non-ATPase base subunit of the 19S RP of the 26S proteasome
YNL155W	2.34	Protein with a role in the ubiquitin-proteasome pathway
SPS22	2.34	Protein of unknown function
HSP31	2.34	Methylglyoxalase that converts methylglyoxal to D-lactate
YOR052C	2.34	AN1-type zinc finger protein of unknown function
BSD2	2.33	Heavy metal ion homeostasis protein
CDC45	2.33	DNA replication initiation factor
YML131W	2.33	Protein of unknown function
SWR1	2.32	Swi2/Snf2-related ATPase
SPH1	2.31	---
PRM6	2.31	Potassium transporter that mediates K+ influx
UFD4	2.31	Ubiquitin-protein ligase
YPC1	2.30	Alkaline ceramidase
SLM1	2.29	Phosphoinositide PI4,5P(2) binding protein
MGM1	2.29	Mitochondrial GTPase
YHR035W	2.29	Activator of Sar1p GTPase activity
TBS1	2.29	Putative protein of unknown function
PHO2	2.28	Homeobox transcription factor
YDR374C	2.27	Posttranscriptional regulator of phosphate metabolism
YTH1	2.27	Essential RNA-binding component of cleavage and polyadenylation factor
RAD54	2.27	DNA-dependent ATPase that stimulates strand exchange
YGL010W	2.27	Putative protein of unknown function
YKL068W-A	2.26	Putative protein of unknown function
TMA17	2.26	ATPase dedicated chaperone that adapts proteasome assembly to stress
GTS1	2.26	Protein involved in Arf3p regulation and in transcription regulation
YNR064C	2.26	Epoxide hydrolase
ESC8	2.26	Protein involved in telomeric and mating-type locus silencing

SLF1	2.26	RNA binding protein that associates with polysomes
UFE1	2.25	t-SNARE protein required for retrograde vesicular traffic
FMP43	2.25	Highly conserved subunit of mitochondrial pyruvate carrier
YMR210W	2.25	Putative acyltransferase with similarity to Eeb1p and Eht1p
SHC1	2.25	Sporulation-specific activator of Chs3p (chitin synthase III)
CPA2	2.25	Large subunit of carbamoyl phosphate synthetase
SSU72	2.25	Phosphatase and transcription/RNA-processing factor
SSE2	2.24	Member of the heat shock protein 70 (HSP70) family
MGR3	2.24	Subunit of the mitochondrial (mt) i-AAA protease supercomplex
PER33	2.24	Protein that localizes to the endoplasmic reticulum
CDC23	2.23	Subunit of the Anaphase-Promoting Complex/Cyclosome
NAS2	2.23	Proteasome-interacting protein
VID27	2.22	Cytoplasmic protein of unknown function
YSW1	2.22	Protein required for normal prospore membrane formation
NTH2	2.22	Putative neutral trehalase
RAD28	2.21	Protein involved in DNA repair
HIS3	2.21	Imidazoleglycerol-phosphate dehydratase
MET22	2.21	Bisphosphate-3'-nucleotidase
LPP1	2.21	Lipid phosphate phosphatase
YOL024W	2.21	Putative protein of unknown function
HHT1 /// HHT2	2.21	Histone H3
TAD2	2.20	Subunit of tRNA-specific adenosine-34 deaminase
HSP42	2.19	Small heat shock protein (sHSP) with chaperone activity
IPL1	2.19	Aurora kinase of conserved chromosomal passenger complex
FMP45	2.19	Integral membrane protein localized to mitochondria
RPO41	2.19	Mitochondrial RNA polymerase
YPL260W	2.19	Putative substrate of cAMP-dependent protein kinase
MSY1	2.19	Mitochondrial tyrosyl-tRNA synthetase
NFI1	2.18	SUMO E3 ligase
ATG9	2.18	Transmembrane protein involved in forming Cvt and autophagic vesicles
RCK1	2.18	Protein kinase involved in the response to oxidative stress
PPE1	2.18	Protein with carboxyl methyl esterase activity
SMF3	2.17	Putative divalent metal ion transporter involved in iron homeostasis
UGA3	2.17	Transcriptional activator for GABA-dependent induction of GABA genes
HSP104	2.17	Disaggregase
YNL194C	2.17	Integral membrane protein
MAG1	2.16	3-methyl-adenine DNA glycosylase
APJ1	2.16	Chaperone with a role in SUMO-mediated protein degradation
TID3	2.15	Component of the kinetochore-associated Ndc80 complex
MSH6	2.15	Protein required for mismatch repair in mitosis and meiosis
YMR187C	2.15	Putative protein of unknown function; YMR187C is not an essential

		gene
CHO1	2.15	Phosphatidylserine synthase
CYC7	2.15	Cytochrome c isoform 2
GRE3	2.14	Aldose reductase
AMA1	2.14	---
SPO23	2.14	Protein of unknown function
RPN4	2.14	Transcription factor that stimulates expression of proteasome genes
YDR239C	2.13	Protein of unknown function
PET123	2.13	Mitochondrial ribosomal protein of the small subunit
PEX27	2.13	Peripheral peroxisomal membrane protein
DUG3	2.13	Component of glutamine amidotransferase
GIP1	2.12	Meiosis-specific regulatory subunit of the Glc7p protein phosphatase
LHS1	2.12	Molecular chaperone of the endoplasmic reticulum lumen
YJR030C	2.12	Putative protein of unknown function
CPT1	2.12	Cholinephosphotransferase
COX23	2.11	Protein that functions in mitochondrial copper homeostasis
CDC15	2.11	Protein kinase of the Mitotic Exit Network
YLL058W	2.11	Putative protein of unknown function with similarity to Str2p
ATG20	2.10	Sorting nexin family member;
LYS1	2.10	Saccharopine dehydrogenase (NAD+, L-lysine-forming)
YLR012C	2.10	Putative protein of unknown function
SSM4	2.10	Ubiquitin-protein ligase involved in ER-associated protein degradation
YJL047C-A	2.10	Putative protein of unknown function
THI72	2.10	Transporter of thiamine or related compound
MET5	2.10	Sulfite reductase beta subunit
SPO19	2.10	Meiosis-specific prospore protein
MIG2	2.10	Zinc finger transcriptional repressor
MRPS17	2.09	Mitochondrial ribosomal protein of the small subunit
HSP78	2.09	Oligomeric mitochondrial matrix chaperone
SNX4	2.09	Sorting nexin
OPT1	2.09	Proton-coupled oligopeptide transporter of the plasma membrane
FAU1	2.09	5,10-methenyltetrahydrofolate synthetase
HSP82	2.09	Hsp90 chaperone
ERO1	2.09	Thiol oxidase required for oxidative protein folding in the ER
MHO1	2.09	Protein of unknown function
ENT1	2.09	Epsin-like protein involved in endocytosis and actin patch assembly
YFL040W	2.09	Putative transporter
DUT1	2.09	deoxyuridine triphosphate diphosphatase
YPL119C-A	2.08	Putative protein of unknown function
ICL1	2.08	Isocitrate lyase
HIS4	2.08	Multifunctional enzyme containing phosphoribosyl-ATP

		pyrophosphatase
YIG1	2.08	Protein that interacts with glycerol 3-phosphatase
YER053C-A	2.08	Protein of unknown function
NRK1	2.08	Nicotinamide riboside kinase
MCH4	2.08	Protein with similarity to mammalian monocarboxylate permeases
PIB1	2.08	RING-type ubiquitin ligase of the endosomal and vacuolar membranes
SSH4	2.08	Specificity factor required for Rsp5p-dependent ubiquitination
ISA1	2.08	Protein required for maturation of mitochondrial [4Fe-4S] proteins
YLR407W	2.07	Putative protein of unknown function
ALD2	2.07	Cytoplasmic aldehyde dehydrogenase
YEL073C	2.07	Putative protein of unknown function
MIH1	2.07	Protein tyrosine phosphatase involved in cell cycle control
YFR039C	2.07	Protein involved in outer spore wall assembly
BIO5	2.07	Putative transmembrane protein involved in the biotin biosynthesis
YOR019W	2.07	Protein of unknown function
RPI1	2.06	Transcription factor
IME2	2.06	Serine/threonine protein kinase involved in activation of meiosis
CHA1	2.06	Catabolic L-serine (L-threonine) deaminase
EEB1	2.06	Acyl-coenzymeA:ethanol O-acyltransferase
YLR455W	2.06	Nuclear protein of unknown function
ORC5	2.06	Subunit of the origin recognition complex
SPR1	2.05	Sporulation-specific exo-1,3-beta-glucanase
YRO2	2.05	Protein of unknown function with similarity to archaeal rhodopsins
STN1	2.05	Telomere end-binding and capping protein
PET309	2.05	Specific translational activator for the COX1 mRNA
YGR035C	2.05	Putative protein of unknown function
MEP3	2.05	Ammonium permease of high capacity and low affinity
BXI1	2.04	Protein involved in apoptosis
KIC1	2.04	Protein kinase of the PAK/Ste20 family
YNR040W	2.04	Putative protein of unknown function
RRG9	2.04	Protein of unknown function
NSE4	2.03	Component of the SMC5-SMC6 complex
YJL070C	2.03	Putative metallo-dependent hydrolase superfamily protein
THI2	2.03	Transcriptional activator of thiamine biosynthetic genes
TAF1	2.02	TFIID subunit
SUL1	2.02	High affinity sulfate permease of the SulP anion transporter family
CDC9	2.02	DNA ligase found in the nucleus and mitochondria
AAD16 /// AAD4	2.02	Putative aryl-alcohol dehydrogenase
MET4	2.02	Leucine-zipper transcriptional activator
AVT4	2.02	Vacuolar transporter
SUE1	2.02	Protein required for degradation of unstable forms of cytochrome c
RGD2	2.02	GTPase-activating protein (RhoGAP) for Cdc42p and Rho5p

YGL015C	2.01	Putative protein of unknown function
LEA1	2.01	Component of U2 snRNP complex
EPT1	2.01	sn-1,2-diacylglycerol ethanolamine- and cholinephosphotranferase
RTT106	2.01	Histone chaperone
RPN8	2.01	Essential non-ATPase regulatory subunit of the 26S proteasome
MCH1	2.01	Protein with similarity to mammalian monocarboxylate permeases
POB3	2.01	Subunit of the heterodimeric FACT complex
YNL195C	2.01	Protein of unknown function
YCR043C	2.01	Putative protein of unknown function
DIG2	2.01	MAP kinase-responsive inhibitor of the Ste12p transcription factor
DAL7	2.01	Malate synthase
YFL034W	2.01	Putative integral membrane protein that interacts with Rpp0p
SNU23	2.01	Component of the U4/U6.U5 snRNP complex
ECM7	2.01	Putative integral membrane protein with a role in calcium uptake
JSN1	2.00	Member of the Puf family of RNA-binding proteins
AIM33	2.00	Putative protein of unknown function
FMC1	2.00	Mitochondrial matrix protein
YJL163C	2.00	Putative protein of unknown function
MSH3	2.00	Mismatch repair protein
ULS1	0.500	Swi2/Snf2-related translocase
ACO2	0.500	Putative mitochondrial aconitase isozyme
RPE1	0.499	D-ribulose-5-phosphate 3-epimerase
CWH41	0.499	Processing alpha glucosidase I
FAA3	0.499	Long chain fatty acyl-CoA synthetase
YEL076C /// YRF1-1 /// YRF1-2 /// YRF1-3 /// YRF1-4 /// YRF1-5 /// YRF1-6 /// YRF1-7 /// YRF1-8	0.498	Helicase encoded by the Y' element of subtelomeric regions
CCR4	0.498	Component of the CCR4-NOT transcriptional complex
FRS2	0.497	Alpha subunit of cytoplasmic phenylalanyl-tRNA synthetase
ETR1	0.496	2-enoyl thioester reductase
PGU1	0.496	Endo-polygalacturonase
EFM1	0.496	Lysine methyltransferase
YJR112W-A	0.495	Putative protein of unknown function
YMR206W	0.495	Putative protein of unknown function
TRM7	0.495	2'-O-ribose methyltransferase
GRH1	0.494	Acetylated cis-Golgi protein
NEW1	0.493	ATP binding cassette protein
YLL066W-B	0.492	Putative protein of unknown function
RRG8	0.492	Putative protein of unknown function
UTP4	0.491	Subunit of U3-containing 90S preribosome and SSU processome complexes

SPE1	0.491	Ornithine decarboxylase
GCN1	0.491	Positive regulator of the Gcn2p kinase activity
LAP2	0.491	Leucyl aminopeptidase yscIV with epoxide hydrolase activity
DIA4	0.491	Probable mitochondrial seryl-tRNA synthetase
CIS3	0.490	Mannose-containing glycoprotein constituent of the cell wall; member of the PIR (proteins with internal repeats) family
IKI3	0.488	Subunit of Elongator complex
SUC2	0.488	Invertase
NCL1	0.487	S-adenosyl-L-methionine-dependent tRNA
YLR281C	0.487	Putative protein of unknown function
YAR010C ///<br>YBR012W-A ///<br>YBR012W-B ///<br>YDR098C-A ///<br>YDR098C-B ///<br>YDR210C-C ///<br>YDR210C-D ///<br>YDR261C-C ///<br>YDR261C-D ///<br>YDR316W-A ///<br>YDR316W-B ///<br>YDR365W-A ///<br>YDR365W-B ///<br>YER137C-A ///<br>YER138C ///<br>YER159C-A ///<br>YER160C ///<br>YGR027W-A ///<br>YGR027W-B ///<br>YGR038C-A ///<br>YGR038C-B ///<br>YGR161C-C ///<br>YGR161C-D ///<br>YHR214C-B ///<br>YHR214C-C ///<br>YJR026W ///<br>YJR027W ///<br>YJR028W ///<br>YJR029W ///<br>YLR157C-A ///<br>YLR157C-B ///<br>YLR227W-A ///<br>YLR227W-B ///<br>YLR256W-A ///	0.486	Retrotransposon TYA Gag gene co-transcribed with TYB Pol

YML039W /// YML040W /// YML045W /// YML045W-A /// YMR050C /// YMR051C /// YNL054W-A /// YNL054W-B /// YOL103W-A /// YOL103W-B /// YOR142W-A /// YOR142W-B /// YPL257W-A /// YPL257W-B /// YPR137C-A /// YPR137C-B /// YPR158C-C /// YPR158C-D /// YPR158W-A /// YPR158W-B		
NIP1	0.486	eIF3c subunit of the eukaryotic translation initiation factor 3
SHR5	0.486	Palmitoyltransferase subunit
SYC1	0.486	Subunit of the APT subcomplex of cleavage and polyadenylation factor
PWP2	0.486	Conserved 90S pre-ribosomal component
IPI3	0.485	Component of the Rix1 complex and pre-replicative complexes
TOS6	0.485	Glycosylphosphatidylinositol-dependent cell wall protein
URA2	0.485	Bifunctional carbamoylphosphate synthetase/aspartate transcarbamylase
SEC12	0.484	Guanine nucleotide exchange factor
SNZ1	0.484	Protein involved in vitamin B6 biosynthesis
PLB1	0.484	Phospholipase B (lysophospholipase) involved in lipid metabolism
GYP8	0.483	GTPase-activating protein for yeast Rab family members
SLI1	0.483	N-acetyltransferase
PHO87	0.482	Low-affinity inorganic phosphate (Pi) transporter
NOP19	0.481	Ribosome biogenesis factor
YDR132C	0.480	Protein of unknown function
YGL159W	0.480	Putative protein of unknown function
YLR179C	0.479	Protein of unknown function with similarity to Tfs1p
PHO11 /// PHO12	0.477	One of three repressible acid phosphatases
TMA23	0.477	Nucleolar protein implicated in ribosome biogenesis
TWF1	0.477	Twinfilin
YDR341C	0.476	Arginyl-tRNA synthetase

NOP1	0.476	Histone glutamine methyltransferase
YFL067W	0.476	Protein of unknown function
MRS1	0.476	Splicing protein
RKI1	0.476	Ribose-5-phosphate ketol-isomerase
DSE3	0.475	Daughter cell-specific protein
ADH4	0.475	Alcohol dehydrogenase isoenzyme type IV
MMR1	0.475	Phosphorylated protein of the mitochondrial outer membrane
NCA3	0.475	Protein involved in mitochondrion organization
TRM13	0.475	2'-O-methyltransferase
HIP1	0.474	High-affinity histidine permease
RPC34	0.472	RNA polymerase III subunit C34
ECM16	0.472	Essential DEAH-box ATP-dependent RNA helicase specific to U3 snoRNP
TAD3	0.471	Subunit of tRNA-specific adenosine-34 deaminase
NUP82	0.471	Linker nucleoporin component of the nuclear pore complex
EMC2	0.471	Member of conserved ER transmembrane complex
POL5	0.470	DNA Polymerase phi
RPL3	0.468	Ribosomal 60S subunit protein L3
PEP1	0.468	Type I transmembrane sorting receptor for multiple vacuolar hydrolases
YRB30	0.468	RanGTP-binding protein
PXR1	0.468	Essential protein involved in rRNA and snoRNA maturation
NOP9	0.467	Essential subunit of U3-containing 90S preribosome
RPL13A	0.467	Ribosomal 60S subunit protein L13A
RRP12	0.467	Protein required for export of the ribosomal subunits
YPR063C	0.466	ER-localized protein of unknown function
DPS1	0.465	Aspartyl-tRNA synthetase
NRM1	0.465	Transcriptional co-repressor of MBF-regulated gene expression
NSL1	0.465	Essential component of the MIND kinetochore complex
RRI1	0.465	Catalytic subunit of the COP9 signalosome (CSN) complex
VMA22	0.463	Protein that is required for vacuolar H+-ATPase function
BNA7	0.462	Formylkynurenine formamidase
BSC1	0.462	Protein of unconfirmed function
TAT2	0.461	High affinity tryptophan and tyrosine permease
CIT2	0.460	Citrate synthase
DIP2	0.460	Nucleolar protein
SKS1	0.458	Putative serine/threonine protein kinase
UTP21	0.457	Subunit of U3-containing 90S preribosome and SSU processome complexes
GFD2	0.457	Protein of unknown function
MEP1	0.456	Ammonium permease
NCA2	0.456	Protein that regulates expression of Fo-F1 ATP synthase subunits
ASE1	0.456	Mitotic spindle midzone-localized microtubule bundling protein

CHS2	0.454	Chitin synthase II
PRP43	0.453	RNA helicase in the DEAH-box family
EXG1	0.452	Major exo-1,3-beta-glucanase of the cell wall
YOR385W	0.452	Putative protein of unknown function
RPA49	0.452	RNA polymerase I subunit A49
NOP58	0.451	Protein involved in producing mature rRNAs and snoRNAs
MCM10	0.451	Essential chromatin-associated protein
SWM1	0.449	Subunit of the anaphase-promoting complex
YAR066W /// YHR214W	0.449	Putative GPI protein
GZF3	0.449	GATA zinc finger protein
NOC2	0.449	Protein involved in ribosome biogenesis
CSI2	0.448	Protein of unknown function
YNL034W	0.448	Putative protein of unknown function
RFC1	0.448	Subunit of heteropentameric Replication factor C
RSA1	0.448	Protein involved in the assembly of 60S ribosomal subunits
MCM7	0.447	Component of the Mcm2-7 hexameric helicase complex
RGM1	0.447	Putative zinc finger DNA binding transcription factor
YGR109W-A /// YGR109W-B /// YIL082W-A	0.447	Retrotransposon TYA Gag gene co-transcribed with TYB Pol; translated as TYA or TYA-TYB polyprotein; Gag is a nucleocapsid protein that is the structural constituent of virus-like particles
PSE1	0.447	Karyopherin/importin that interacts with the nuclear pore complex
CIN4	0.446	GTP-binding protein involved in beta-tubulin (Tub2p) folding
RPL8A	0.446	"Ribosomal 60S subunit protein L8A
SRP40	0.445	Nucleolar serine-rich protein
DBP3	0.445	RNA-Dependent ATPase
MPC54	0.445	Component of the meiotic outer plaque
FLC1	0.444	Putative FAD transporter
RPG1	0.444	eIF3a subunit of the eukaryotic translation initiation factor 3
HEM3	0.444	Porphobilinogen deaminase
RBD2	0.443	Possible rhomboid protease
GTB1	0.442	Glucosidase II beta subunit
RPS0A	0.441	Ribosomal 40S subunit protein S0A
MRI1	0.441	5'-methylthioribose-1-phosphate isomerase
SNU114	0.441	GTPase component of U5 snRNP involved in mRNA splicing via spliceosome
YPK2	0.441	Protein kinase similar to serine/threonine protein kinase Ypk1p
YPT31	0.441	Rab family GTPase
PRS3	0.439	5-phospho-ribosyl-1(alpha)-pyrophosphate synthetase
YOR342C	0.439	Protein of unknown function
GDT1	0.439	Protein of unknown function involved in calcium homeostasis
MIS1	0.439	Mitochondrial C1-tetrahydrofolate synthase
OCH1	0.438	Mannosyltransferase of the cis-Golgi apparatus

ARD1	0.438	Subunit of protein N-terminal acetyltransferase NatA
URA5	0.438	Major orotate phosphoribosyltransferase (OPRTase) isozyme
ENT4	0.438	Protein of unknown function
INH1	0.437	Protein that inhibits ATP hydrolysis by the F1F0-ATP synthase
UTP14	0.436	Subunit of U3-containing Small Subunit processome complex
YBR230W-A	0.436	Putative protein of unknown function
TGS1	0.435	Trimethyl guanosine synthase
RPL4B	0.434	Ribosomal 60S subunit protein L4B
AAH1	0.434	Adenine deaminase
DIT1	0.433	Sporulation-specific enzyme required for spore wall maturation
SRB2	0.430	Subunit of the RNA polymerase II mediator complex
DPM1	0.429	Dolichol phosphate mannose synthase of the ER membrane
YNR066C	0.427	Putative membrane-localized protein of unknown function
YLR063W	0.427	Methyltransferase required for m3U2843 methylation of the 25S rRNA
NUF2	0.427	Component of the kinetochore-associated Ndc80 complex
WTM1	0.427	Transcriptional modulator
CDC5	0.425	Polo-like kinase
BER1	0.425	Protein involved in microtubule-related processes
NKP2	0.423	Central kinetochore protein and subunit of the Ctf19 complex
YPS3	0.423	Aspartic protease
PUS4	0.423	Pseudouridine synthase
LYS12	0.423	Homo-isocitrate dehydrogenase
YBL112C /// YBL113C /// YEL077C /// YHR219W /// YIL177C /// YJL225C /// YLL066C /// YLL067C /// YML133C /// YPR204W /// YRF1-1 /// YRF1-2 /// YRF1-3 /// YRF1-4 /// YRF1-5 /// YRF1-6 /// YRF1-7 /// YRF1-8	0.422	Putative Y' element ATP-dependent helicase
GCD10	0.421	Subunit of tRNA (1-methyladenosine) methyltransferase with Gcd14p
IMD4	0.421	Inosine monophosphate dehydrogenase
SDA1	0.420	Protein required for actin organization and passage through Start
TCD1	0.420	tRNA threonylcarbamoyladenosine dehydratase
NOP8	0.420	Nucleolar protein required for 60S ribosomal subunit biogenesis
NAT1	0.419	Subunit of protein N-terminal acetyltransferase NatA

SES1	0.419	Cytosolic seryl-tRNA synthetase
SPO73	0.418	Meiosis-specific protein of unknown function
FMP41	0.418	Putative protein of unknown function
PSP2	0.416	Asn rich cytoplasmic protein that contains RGG motifs
GYP6	0.416	GTPase-activating protein (GAP) for yeast Rab family member Ypt6p
FEN1	0.416	Fatty acid elongase
PYK2	0.416	Pyruvate kinase
SEC11	0.412	18kDa catalytic subunit of the Signal Peptidase Complex
RNR2	0.411	Ribonucleotide-diphosphate reductase
MAK16	0.411	Essential nuclear protein
HCA4	0.410	DEAD box RNA helicase
ELP2	0.410	Subunit of Elongator complex
PSR1	0.409	Plasma membrane associated protein phosphatase
SRP101	0.406	Signal recognition particle (SRP) receptor alpha subunit
ROK1	0.404	RNA-dependent ATPase
RRB1	0.404	Nuclear protein involved in early steps of ribosome biogenesis
MOD5	0.403	Delta 2-isopentenyl pyrophosphate
FIT3	0.401	Mannoprotein that is incorporated into the cell wall
GLY1	0.401	Threonine aldolase
RRN5	0.401	Protein involved in transcription of rDNA by RNA polymerase I
PTR2	0.400	Integral membrane peptide transporter
NUP145	0.400	Essential protein with distinct roles in two nuclear pore subcomplexes
PRM7	0.400	Pheromone-regulated protein
POP3	0.396	Subunit of both RNase MRP and nuclear RNase P
YCR108C	0.394	Putative protein of unknown function
TRM10	0.393	tRNA methyltransferas
PEX34	0.392	Protein that regulates peroxisome populations
YPR011C	0.390	Mitochondrial transporter
RAS1	0.389	GTPase involved in G-protein signaling in adenylate cyclase activation
MRF1	0.385	Mitochondrial translation release factor
SPO1	0.384	Meiosis-specific prospore protein
RPA190	0.383	RNA polymerase I largest subunit A190
BUD8	0.383	Protein involved in bud-site selection
LOT5	0.383	Protein of unknown function
ABZ2	0.383	Aminodeoxychorismate lyase
SPS4	0.382	Protein whose expression is induced during sporulation
TYE7	0.381	Serine-rich protein that contains a bHLH DNA binding motif
CTS1	0.380	Endochitinase
CTR3	0.380	High-affinity copper transporter of the plasma membrane
ODC1	0.379	Mitochondrial inner membrane transporter

RRP5	0.376	RNA binding protein involved in synthesis of both 18S and 5.8S rRNAs
CTR1	0.375	High-affinity copper transporter of the plasma membrane
MDJ2	0.374	Constituent of the mitochondrial import motor
KNH1	0.373	Protein with similarity to Kre9p
BUD9	0.373	Protein involved in bud-site selection
BUD17	0.370	Putative pyridoxal kinase
SEC14	0.370	Phosphatidylinositol/phosphatidylcholine transfer protein
NUC1	0.370	Major mitochondrial nuclease
ALK1	0.369	Protein kinase
YNL277W-A	0.366	Putative protein of unknown function
YGL185C	0.365	Putative protein with sequence similar to hydroxyacid dehydrogenases
REX2	0.363	3'-5' RNA exonuclease
YBR197C	0.362	Protein of unknown function
AYR1	0.362	Bifunctional triacylglycerol lipase and 1-acyl DHAP reductase
FLO11	0.361	GPI-anchored cell surface glycoprotein
UBA3	0.361	Protein that activates Rub1p (NEDD8) before neddylation
PRY1	0.360	Sterol binding protein involved in the export of acetylated sterols
PUF6	0.360	Pumilio-homology domain protein
PAC1	0.359	Involved in nuclear migration
PUF3	0.357	Protein of the mitochondrial outer surface
SUT1	0.357	Transcription factor of the Zn(II)2Cys6 family
YNL058C	0.356	Putative protein of unknown function
PPM2	0.356	AdoMet-dependent tRNA methyltransferase
PCL1	0.353	Cyclin, interacts with cyclin-dependent kinase Pho85p
PHO3	0.351	Constitutively expressed acid phosphatase similar to Pho5p
SRL1	0.351	Mannoprotein that exhibits a tight association with the cell wall
BUD31	0.350	Component of the SF3b subcomplex of the U2 snRNP
CLB1	0.348	B-type cyclin involved in cell cycle progression
YDL241W	0.348	Putative protein of unknown function
YNL042W-B /// YOL013W-A /// YOR072W-B	0.345	Putative protein of unknown function
SLD7	0.344	Protein with a role in chromosomal DNA replication
SPB1	0.342	AdoMet-dependent methyltransferase
KIN3	0.340	Nonessential serine/threonine protein kinase
RTA1	0.339	Protein involved in 7-aminocholesterol resistance
YJR129C	0.337	S-adenosylmethionine-dependent methyltransferase
NOG2	0.336	Putative GTPase
NAT5	0.336	Subunit of protein N-terminal acetyltransferase NatA
SVS1	0.336	Cell wall and vacuolar protein
IRC8	0.336	Bud tip localized protein of unknown function

PUS9	0.335	Mitochondrial tRNA:pseudouridine synthase
PRM7	0.334	Pheromone-regulated protein
TDA10	0.334	ATP-binding protein of unknown function
ALF1	0.333	Alpha-tubulin folding protein
IRC24	0.331	Putative benzil reductase
YFR018C	0.330	Putative protein of unknown function
HXK2	0.330	Hexokinase isoenzyme 2
YNL046W	0.329	Putative protein of unknown function
CDC20	0.329	Activator of anaphase-promoting complex/cyclosome
SUN4	0.327	Cell wall protein related to glucanases
YOX1	0.326	Homeobox transcriptional repressor
MDN1	0.326	Huge dynein-related AAA-type ATPase
LYS9	0.322	Saccharopine dehydrogenase
OAR1	0.321	Mitochondrial 3-oxoacyl-[acyl-carrier-protein] reductase
RPA135	0.318	RNA polymerase I second largest subunit A135
ASH1	0.315	Component of the Rpd3L histone deacetylase complex
PUS7	0.310	Pseudouridine synthase
CLN3	0.310	G1 cyclin involved in cell cycle progression
YKR045C	0.309	Putative protein of unknown function; epitope-tagged protein localizes to the cytoplasm
YMR315W-A	0.309	Putative protein of unknown function
HXT13	0.303	Hexose transporter
AIM20	0.303	Putative protein of unknown function
RFU1	0.302	Protein that inhibits Doa4p deubiquitinating activity
SRO9	0.302	Cytoplasmic RNA-binding protein
FET4	0.302	Low-affinity Fe(II) transporter of the plasma membrane
AIM44	0.299	Protein that regulates Cdc42p and Rho1p
EGT2	0.298	Glycosylphosphatidylinositol (GPI)-anchored cell wall endoglucanase
VPS75	0.294	NAP family histone chaperone
PRS1	0.291	5-phospho-ribosyl-1(alpha)-pyrophosphate synthetase
SFC1	0.289	Mitochondrial succinate-fumarate transporter
HPA3	0.288	D-Amino acid N-acetyltransferase that detoxifies D-amino acids
DSE2	0.286	Daughter cell-specific secreted protein with similarity to glucanases
COX19	0.285	Protein required for cytochrome c oxidase assembly
SCW10	0.283	Cell wall protein with similarity to glucanases
YPL067C	0.282	Putative protein of unknown function
SNM1	0.279	Ribonuclease MRP complex subunit
FUI1	0.276	High affinity uridine permease
FET3	0.275	Ferro-O2-oxidoreductase
YER152C	0.271	Protein with 2-aminoadipate transaminase activity
SMC4	0.270	Subunit of the condensin complex
MYO1	0.266	Type II myosin heavy chain

AAC1	0.263	Mitochondrial inner membrane ADP/ATP translocator
PRY3	0.256	Cell wall-associated protein involved in export of acetylated sterols
YOL019W	0.255	Protein of unknown function
YVH1	0.253	Protein phosphatase
BUD4	0.253	Anillin-like protein involved in bud-site selection
TAT1	0.252	Amino acid transporter for valine, leucine, isoleucine, and tyrosine
CLN1	0.248	G1 cyclin involved in regulation of the cell cycle
SWI5	0.244	Transcription factor that recruits Mediator and Swi/Snf complexes
DUS3	0.243	Dihydrouridine synthase
YJR149W	0.238	Putative protein of unknown function;
SCW11	0.234	Cell wall protein with similarity to glucanases
CLB2	0.234	B-type cyclin involved in cell cycle progression
PRY2	0.231	Sterol binding protein involved in the export of acetylated sterols
RAX1	0.231	Protein involved in bud site selection during bipolar budding
HMS2	0.219	Protein with similarity to heat shock transcription factors
YCR015C	0.206	Putative protein of unknown function
DYN1	0.202	Cytoplasmic heavy chain dynein
AGX1	0.183	Alanine:glyoxylate aminotransferase
SNO1	0.179	Protein of unconfirmed function
FBP1	0.176	Fructose-1,6-bisphosphatase
SIM1	0.175	Protein of the SUN family
PCK1	0.171	Phosphoenolpyruvate carboxykinase
HXT13	0.155	Hexose transporter
SFG1	0.151	Nuclear protein putative transcription factor
FDH1	0.130	NAD(+)-dependent formate dehydrogenase
PHO5	0.070	Repressible acid phosphatase
MAL12 /// MAL32	0.046	Maltase
SOR1 /// SOR2	0.043	Sorbitol dehydrogenase
HXT15 /// HXT16	0.027	Protein of unknown function with similarity to hexose transporters
